# Revealing hidden drivers of Lassa fever through a model-informed approach for reproducing and predicting disease dynamics and guiding control strategies

**DOI:** 10.1038/s41598-025-01176-y

**Published:** 2025-09-30

**Authors:** Hemaho B. Taboe, Sergei S. Pilyugin, Calistus N. Ngonghala

**Affiliations:** 1 Department of Mathematics, University of Florida, Gainesville, FL 32611 USA; 2 Laboratoire de Biomathématiques et d’Estimations Forestières, University of Abomey-Calavi, Cotonou, Bénin; 3 Emerging Pathogens Institute, Gainesville, FL 32610 USA; 4 Harvard Radcliffe Institute of Advanced Studies, 10 Garden St., Cambridge, MA 02138 USA; 5 Harvard Medical School, Boston, MA 02115 USA

**Keywords:** Vector-borne disease, Asymptomatic and vertical transmission, Environmental contamination, Competitor species, Lassa fever virus control, Applied mathematics, Computational models, Epidemiology

## Abstract

Lassa fever (LF), caused by the Lassa virus and transmitted primarily by *Mastomys natalensis* rodents, is a severe hemorrhagic disease endemic to West Africa, particularly Nigeria, with significant morbidity and mortality rates. This study develops dynamic models for LF, incorporating crucial but often overlooked factors such as vertical transmission (i.e., transmission from parents to their offsprings) in rodents, surface contamination, and asymptomatic human carriers. The persistence of the disease is shown analytically. Using data from Nigeria to train the models, the impact of various control and mitigation measures is assessed. The results of the study reveal that asymptomatic individuals are key drivers of LF and that including additional LF virus transmission pathways, e.g., vertical transmission and environmental contamination, increases the estimated reproduction number threefold compared to previous studies. Models incorporating rodent dynamics show the highest disease prevalence, highlighting the critical role of rodent control. Specifically, effective interventions using only rodent control measures require maintaining rodent populations below a specific threshold. In addition, a multifaceted approach, combining antiviral treatment, environmental disinfection, and personal protective equipment, significantly enhances disease control, while the introduction of a competitor rodent species can drastically reduce human and rodent infections. Ultimately, the study underscores the need for integrated, multifaceted strategies, including targeting rodents, asymptomatic cases, and comprehensive treatment and disinfection protocols, for effective LF management.

**Supplementary Information:**

The online version contains supplementary material available at 10.1038/s41598-025-01176-y.

## Introduction

Lassa fever (LF) is a significant public health concern in West Africa, particularly in Nigeria where it was first discovered in 1969^1^. It causes acute viral hemorrhagic fever, with high morbidity and mortality, affecting an estimated 100,000 to 300,000 people and causing approximately 5,000 human deaths annually^2^. However, these figures likely underestimate the true extent of the disease, as many cases go unidentified due to individuals not seeking medical care, and the fact that a significant proportion of cases are asymptomatic. Asymptomatic cases of LF constitute a significant proportion (up to $80\%$) of LF infections^3^. Despite the lack of overt symptoms, asymptomatic individuals shed the virus, contributing to LF prevalence within endemic regions by serving as hidden reservoirs for continued transmission. This challenges LF control efforts, highlighting the need to understand asymptomatic infections and their role in transmission to develop effective strategies^4^.

Lassa fever control is also challenging given its diverse transmission pathways, environmental factors, and seasonal patterns, necessitating comprehensive control strategies. It is a zoonotic disease that is caused by a single-stranded RNA virus known to be in the Arenaviridae’s family^1^ and primarily transmitted by *Mastomys natalensis* rodents, the natural host of the virus, leading to direct or indirect human exposure to the virus. Secondary transmission among humans occurs through direct human-to-human contact with bodily fluids, such as blood, saliva, urine, or faeces, of infected individuals, as well as through aerosol transmission, where the virus can be inhaled from contaminated air^3,5,6^. Environmental and/or surface transmission involves LF persistence on contaminated surfaces and exposure through various means, including rodent excreta and contaminated food, as well as contaminated medical equipment used at hospitals^3,5^. While the most significant route of transmission is through rodent-to-human contact, particularly via contaminated surfaces or food, human-to-human transmission can also contribute to outbreaks, albeit less frequently^3,4^. Rodent-to-rodent transmission is crucial for LF circulation within rodent populations, affecting spillover risk to humans^5^. Vertical transmission of LF in rodents has also been reported^5^. This mode of transmission can contribute to the prevalence of LF within the rodent population by establishing new cases among newborns. While vertical transmission of LF has been documented, its precise impact on disease prevalence remains an area of ongoing research^5^. Another potential secondary route of transmission is through sexual contact among humans^5^. Understanding these transmission dynamics is vital for implementing effective prevention and control measures, including rodent control strategies, improved sanitation, infection control practices, and community education, to mitigate LF transmission and reduce disease burden.

Control and mitigation methods for Lassa fever (LF) virus include rodent interventions to reduce the population of *Mastomys natalensis*, the primary reservoir, and environmental sanitation to minimize rodent-human contact^7^. Additionally, promotion of personal protective measures such as hand hygiene, use of protective clothing and gloves, as well as avoidance of contact with potentially contaminated materials is crucial. Given that LF can be transmitted through contact with contaminated surfaces and bodily fluids, protective clothing and gloves act as barriers, preventing direct contact between the virus and the skin. By wearing gloves, mask and protective clothing, individuals reduce the risk of exposure to LF during activities such as cleaning, handling potentially infected materials, or providing medical care to patients with LF. This preventive measure is particularly important in healthcare settings, where the risk of LF transmission is higher due to close contact with infected individuals and contaminated medical equipment. Implementing strict glove-wearing protocols, along with other infection control measures, can effectively reduce the risk of LF transmission and contribute to overall disease control efforts. Early detection and isolation of cases, along with prompt treatment and supportive care are crucial for improving patient outcomes, reducing mortality associated with LF infection, and preventing secondary transmission among humans. Treatment of LF primarily involves supportive care to manage symptoms and complications. Ribavirin, an antiviral medication, is the recommended treatment for confirmed cases, particularly when administered early in the course of the disease^2,3^. Additionally, maintaining hydration, managing fever, and providing supportive measures such as respiratory support, if necessary, are essential components of patient care. While these methods are commonly used and effective in reducing LF transmission, they may incur moderate costs for implementation and sustained efforts. Although, no specific vaccine is currently available for LF, vaccination strategies are being explored, while more advanced methods such as genetic manipulation of the rodent population remain experimental. Cost-effective approaches, such as community-based education and surveillance programs can contribute to sustained LF control and elimination efforts. By integrating cost-effective approaches with mathematical modeling, we can improve LF control and elimination strategies, making interventions more informed and efficient.

Mathematical modeling plays a crucial role in understanding and controlling LF by providing insights into disease transmission dynamics and guiding public health interventions; it provides a valuable tool for predicting disease trends, evaluating control strategies, and informing evidence-based decision-making in the fight against LF. Various mathematical models have been developed to address key questions related to LF, including its epidemiology, transmission routes, and the impact of control measures^5,8–14^. Some of these models focus on assessing the effectiveness of interventions such as vector control in reducing LF transmission^11^, while others investigate the role of environmental factors, such as rainfall, climate and habitat suitability, in shaping LF prevalence and spread^13^. For instance, McKendrick *et al.*^8^ used a mathematical model with a periodic rodent reproduction term to capture seasonal LF infection patterns and recommended intensified control efforts from December to March. Obabiyi and Akindele^12^ used an age-structured mathematical model to emphasize early detection and prompt/effective treatment as the optimal LF control strategy, alongside maintaining hospital hygiene and controlling transmission in animal hosts. Okuonghae *et al.*^15^ explored the impact of a hypothetical vaccine through a mathematical framework, while Ogabi *et al.*^16^ analyzed disease extinction conditions using an SIR model. Onuorah *et al.*^11^ used a sex-structured model to study the effects of condom use and rodent control on LF invasion. Eraikhuemen and Eguasa^17^ proposed a SEIS model for LF transmission dynamics in which rodent growth is modeled with a logistic function. Ibrahim *et al.*^18^ examined LF seasonality using periodic functions and LF data from Nigeria. Many of these models overlook critical aspects of LF transmission including vertical transmission in the rodent population, although studies have confirmed the possibility of this transmission pathway^5,19–21^, LF transmission through environmental contamination, and the contribution of asymptomatically infectious individuals in LF dynamics, highlighting the need for further research in this area. In particular, accounting for these transmission pathways can improve LF modeling accuracy and effectiveness in guiding control strategies.

In this study we develop a model framework for the dynamics of LF that accounts for commonly overlooked features of the disease such as vertical transmission in rodents (i.e., transmission from rodent parents to their offsprings), transmission from contaminated surfaces including food, kitchen utensils, medical equipment, and transmission by asymptomatically infectious humans. The model is used to answer the following questions: 1) What is the impact of incorporating vertical transmission in rodent populations, LF contamination of surfaces, and asymptomatic transmission on the transmission dynamics of LF? 2) How do different control and mitigation measures which include regular disinfection of contaminated surfaces, personal hygiene, protective glove, mask and cloth usage, and limiting the host rodent population (e.g., through rodent killing or through the introduction of a competitor rodent species) influence Lassa Fever dynamics? To address these questions, the model is studied analytically for the existence and stability of equilibria, with the reproduction number computed through the Next Generation Operator approach^22,23^. It is parameterized by fitting to epidemiological data from Nigeria. The model was simulated to understand the long-term behavior of the system and assess the impact of intervention measures on key model outcomes. Additionally, a global sensitivity analysis using the Hypercube Sampling (LHS) approach with Partial Rank Correlation Coefficients (PRCC) and the extended Fourier Amplitude Sensitivity Test (eFAST)^24^ were used to identify key control parameters.

This paper is organized as follows. In Section 2, we formulate the mathematical model of the LF transmission and describe the state variables and the parameters of the said model. In Section 3, we present relevant analytical results regarding the basic qualitative dynamics of the model including the existence of various types of equilibria and their stability. Section 4 is dedicated to the numerical study of the model and it describes the model calibration as well as the uncertainty and sensitivity analyses. In Section 4, we also simulate the effectiveness of proposed control measures. In Section 5, we discuss the main results of our study and describe its caveats and limitations. Section 6 contains the concluding remarks.

## Methods

### The model

**Fig. 1:**
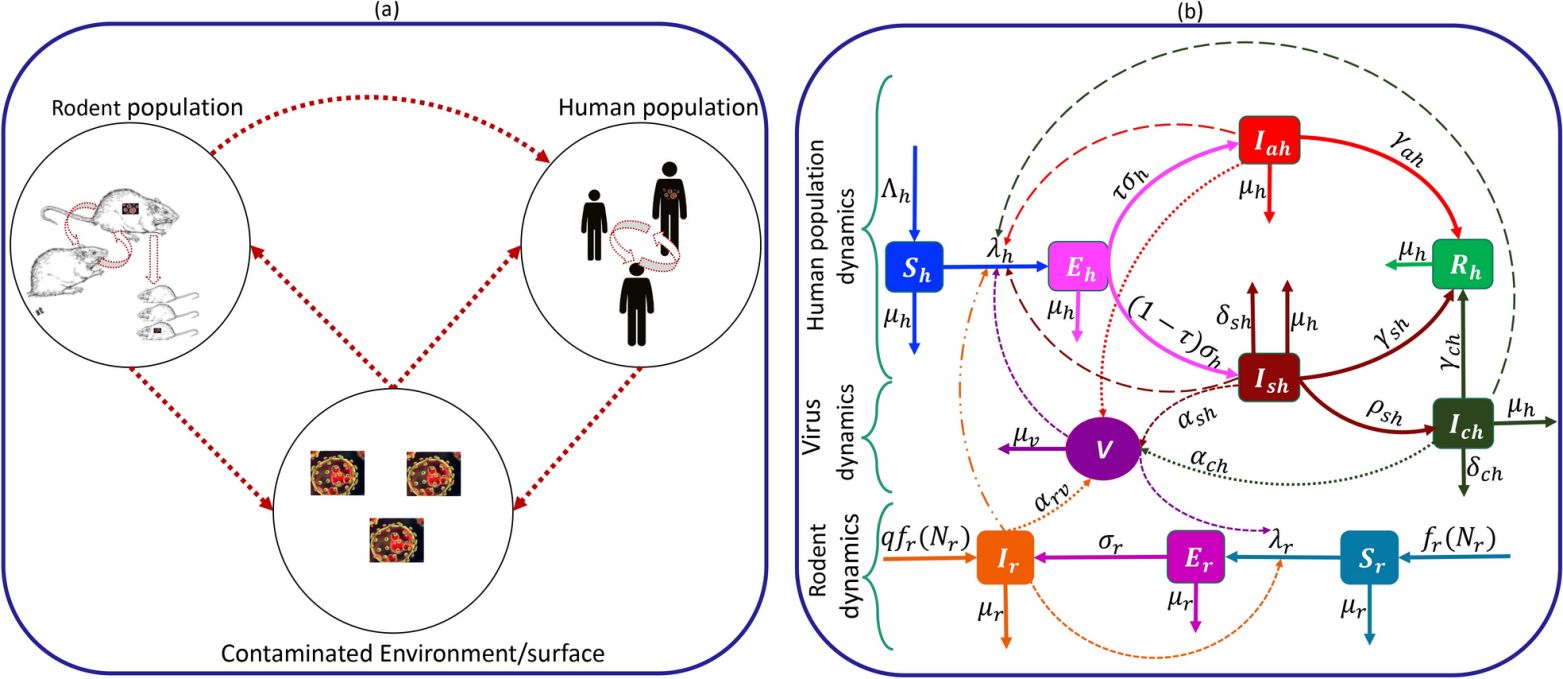
(**a**) Conceptual illustration of Lassa fever virus shedding onto environmental surfaces from humans and rodents, and transmission within human populations, rodent populations, from contaminated surfaces to humans and rodents, and from rodents to humans. (**b**) Schematics of the Lassa Fever Virus (LF) model framework. Variables for human compartments have the subscript *h*, while those for rodent compartments have the subscript *r*. The virus compartment variable is denoted by *V*. Light solid curves show births and deaths, while thick solid curves show transitions due to LF in the human and rodent populations. Interactions between humans, rodents, and the virus in environmental surfaces that lead to infection are denoted by dashed, dashed-dotted, and dotted curves. The force of infection in humans (rodents) is denoted by $\lambda _h$ ($\lambda _r$). The state variables are described in Table 1 and model parameters are described in Tables 2-3.

A compartmental model is developed to analyze LF transmission dynamics, incorporating various pathways such as human-to-human, rodent-to-human, rodent-to-rodent, human-to-environment, and rodent-to-environment transmission. The human population is divided into susceptible ($S_h$), exposed ($E_h$), symptomatic infectious ($I_{sh}$), asymptomatic infectious ($I_{ah}$), confirmed ($I_{ch}$), and recovered ($R_h$) classes, adhering to the Susceptible-Exposed-Infectious-Recovered (SEIR)-type framework. Meanwhile, the rodent population is divided into susceptible ($S_r$), exposed ($E_r$), and symptomatic infectious ($I_r$) rodents, based on the SEI-type framework, considering the non-recovery of rodents from LF. Hence, at any given time (*t*), the total populations for humans and rodents are represented by $N_h(t) = S_h(t) + E_h(t) + I_{ah}(t) + I_{sh}(t) + I_{ch}(t) + R_h(t)$ and $N_r(t) = S_r(t) + E_r(t) + I_r(t)$. The density of virus in the environment, i.e., on contaminated surfaces, material, equipment, etc., is denoted by *V*. For clarity, Tables 1-3 provide definitions of the various variables and parameters used in the model. A visual representation of the model framework is presented in Fig. 1.

Assuming that there is no vertical transmission within the human population, all human births are into the susceptible class at rate $\Lambda _h$ and natural deaths in each of the human classes is at per capita rate $\mu _h$. Susceptible humans contract Lassa fever from symptomatic infectious humans at rate $\beta _{sh}I_{sh}/N_h$, asymptomatic infectious humans at rate $\beta _{ah}I_{ah}/N_h$, and confirmed infectious humans at rate $\beta _{ch}I_{ch}/N_h$, infectious rodents at rate $\beta _{rh}I_r/N_h$, and from contaminated surfaces or equipment at rate $\beta _{vh}V$, where $\beta _{jh}, j \in \{s, a, c, r, v\}$ is the transmission rate from infectious source *j*. Hence, the force of infection for humans is $\lambda _h = \frac{\beta _{sh}I_{sh} + \beta _{ah}I_{ah} + \beta _{ch}I_{ch}}{N_h} + \frac{\beta _{rh}I_r}{N_h} + \beta _{vh}V$ and $\lambda _hS_h$ newly infected susceptible humans progress to the exposed class. At the end of the incubation period ($1/\sigma _h$), a proportion $(1 - \tau )\sigma _h$ (where $0 \le \tau \le 1$) of exposed humans exhibit symptoms of the disease, while the remaining fraction ($\tau \sigma _h$) does not. Symptomatic infectious individuals are identified at rate $\rho _{sh}$, recover from infection at rate $\gamma _{sh}$, or die from LF at rate $\delta _{sh}$. Asymptomatic infectious individuals recover from LF at rate $\gamma _{ah}$, while confirmed cases either recover from LF at rate $\gamma _{ch}$ or die from LF at rate $\delta _{ch}$. Since human immunity to LF is permanent^3,5^, individuals who have recovered remain in the recovered class until they die naturally or from other causes.

Rodent births are modeled with a logistic function, with intrinsic birth rate $\Omega _r$ and carrying capacity *K*, while natural deaths in all rodent classes are at rate, $\mu _r$. Studies, including those in^5^ have shown that vertical transmission of LF in rodent populations is possible. Hence, a portion $f_r\left(N_r\right) [S_r + E_r + (1 - q)I_r]$, where $0 \le q \le 1$, and $f_r(N_r) = \Omega _r\left( 1 - \frac{N_r}{K}\right)$, of all newly born rodents joins the susceptible class, while the remaining portion $qf_r(N_r)I_r$, joins the infectious rodent class. The force of infection for rodents is $\lambda _r = \frac{\beta _{rr I_r}}{N_r} + \beta _{vr}V$ implying $\lambda _rS_r$ newly infected rodents progress to the exposed class. Exposed rodents progress to the infectious rodent class at rate $\sigma _r$. It is assumed that rodents neither die nor recover from LF infection^5^. The virus is shed to the environment (e.g., surfaces or equipment) by symptomatic, asymptomatic, and confirmed infectious humans, as well as infectious rodents, at rates denoted by $\alpha _{jv}$, where $j \in \{s, a, c, r\}$, and decays at rate $\mu _v$. Furthermore, it is assumed that human-to-rodent transmission and sexual transmission are negligible.

**Table 1:** Definitions of the state variables used in the model. Human variables, with subscript *h*, and rodent variables with subscript *r*, are measured in individuals (humans or rodents) at time *t* (in epidemiological weeks), while the environmental virus density is measured in virus per unit surface area at time *t*.

**Variable**	** Definition (Description)**
$S_{h}$	Susceptible humans: Individuals who have not contracted LF
$E_{h}$	Exposed humans: infected individuals, who are not infectious yet
$I_{ah}$	Asymptomatic humans: individuals who do not exhibit clinical symptoms (after the incubation)
$I_{sh}$	Symptomatic infectious humans: individuals who exhibit clinical symptoms
$I_{ch}$	Confirmed or reported cases of LF, including those under treatment
$R_{h}$	Recovered humans: previously infectious individuals, who have recovered
$S_{r}$	Susceptible rodents: rodents that have not contracted LF yet
$E_{r}$	Exposed rodents: rodents that have contracted LF, but are not infectious yet
$I_{r}$	Infectious rodents: infected rodents that can transmit the disease
*V*	Density of the virus in the environment (i.e., on contaminated surfaces, materials, equipment, etc)

Using the model schematics in Fig. 1, together with the model variable and parameter descriptions (Tables 1-3), we obtain the following system of equations for the dynamics of the disease within humans, rodents, and the environment:

2.1 $$\begin{aligned} \dot{S}_{h}= & \Lambda _{h} - \left( \frac{\beta _{ah}I_{ah} + \beta _{sh}I_{sh} + \beta _{ch}I_{ch}}{N_{h}} + {\frac{\beta _{rh} I_{r}}{N_r}}+\beta _{vh}V\right) S_{h} - \mu _{h}S_{h},\nonumber \\ \dot{E}_{h}= & \left( \frac{\beta _{ah}I_{ah}+\beta _{sh}I_{sh} + \beta _{ch}I_{ch}}{N_{h}} + {\frac{\beta _{rh} I_{r}}{N_r}}+\beta _{vh}V\right) S_{h}- (\mu _h + \sigma _h) E_{h},\nonumber \\ \dot{I}_{ah}= & \tau \,\sigma _h E_{h} -(\mu _h + \gamma _{ah})\, I_{ah},\nonumber \\ \dot{I}_{sh}= & (1 - \tau )\,\sigma _h E_{h}- (\mu _h +\gamma _{sh} +\rho _{sh}+\delta _{sh}) I_{sh},\nonumber \\ \dot{I}_{ch}= & \rho _{sh} I_{sh} -(\mu _h + \gamma _{ch} + \delta _{ch}) I_{ch},\nonumber \\ \dot{R}_{h}= & \gamma _{ch}\, I_{ch}+ \gamma _{sh} I_{sh} +\gamma _{ah} I_{ah} - \mu _{h} R_{h}, \nonumber \\ \dot{S}_{r}= & \Omega _r\left( 1 - \frac{N_r}{K}\right) \left[ S_r +E_r+ (1-q) I_{r}\right] - \left( \frac{\beta _{rr}I_{r}}{N_r} + \beta _{vr}V\right) S_r- \mu _{r}S_{r},\nonumber \\ \dot{E}_{r}= & \left( \frac{\beta _{rr}I_{r}}{N_r} + \beta _{vr}V\right) S_r - ( \sigma _r +\mu _{r})E_{r},\nonumber \\ \dot{I}_{r}= & q\Omega _r\left( 1-\frac{N_r}{K}\right) I_{r} +\sigma _r\,E_{r} - \mu _{r} I_{r},\nonumber \\ \dot{V}= & {\alpha _{rv}I_r +\alpha _{av} I_{ah}+ \alpha _{sv} I_{sh}}+\alpha _{cv}I_{ch} - \mu _vV. \end{aligned}$$

For notational convenience, we adopt the following parameter groupings henceforth:

2.2 $$\begin{aligned} & B_{e} = \mu _h + \sigma _h, B_{a} = \mu _h + \gamma _{ah}, B_{s} = \mu _h +\gamma _{sh} +\rho _{sh}+\delta _{sh}, B_{c} = \mu _h \nonumber \\ & +\gamma _{ch}+\delta _{ch}, B_{r} = \sigma _{r} + \mu _r, \tilde{\Omega }_r =\Omega _r-\mu _r, \tilde{K}=K\tilde{\Omega }_r/\Omega _r. \end{aligned}$$

### Analytical methods

#### Feasible and positive invariant region

The feasible and positive invariant region of the model will be derived by identifying conditions that ensure all state variables remain non-negative and bounded over time. Specifically, analysis of the model (2.1) will be performed within the feasible region $\mathbb {D} = \mathbb {D}_h \times \mathbb {D}_r \times \mathbb {D}_v \subseteq \mathbb {R}^{6}_{+} \times \mathbb {R}^{3}_{+} \times \mathbb {R}_{+},~where ~,$

$\mathbb {D}_h = \left\{ (S_h, E_h, I_{ah}, I_{sh}, I_{ch}, R_h) \in \mathbb {R}^{6}_{+}: 0 \le S_h, E_h, I_{ah}, I_{sh}, I_{ch}, R_h \le \frac{\Lambda _h}{\mu _h} \right\}, \mathbb {D}_r = \left\{ (S_r, E_r, I_r) \in \mathbb {R}^{3}_{+}: 0 \le S_r, E_r, I_r \le \tilde{K} \right\} ,$ and $\mathbb {D}_v = \left\{ V \in \mathbb {R}_{+}: 0 \le V \le \frac{\alpha _{rv}\tilde{K}}{\mu _v} +(\alpha _{av} +\alpha _{sv}+\alpha _{cv})\frac{\Lambda _h}{\mu _h\mu _v} \right\}$ (see Section S1 of the Supplementary Information (SI) for details).

#### Subsystems, equilibria, and reproduction numbers

Specific subsystems or subsets of model (2.1) are analyzed prior to examining the full system. Studying these sub-systems is important because it simplifies the analysis, enabling us to isolate and understand the dynamics of key components without the complexity of the full system. Specifically, this approach identifies key parameters, interactions, and subsystems driving model behavior, enables stepwise validation, improves computational efficiency, and guides effective intervention design. Equilibria of the sub-systems and the full model will be obtained by setting the left hand sides to zero and solving the resulting systems of algebraic equations for the state variables. The reproduction numbers will be computed using the next generation operator approach^22,23^, which involves linearizing the sub-systems and the full model around the corresponding disease-free equilibrium (DFE) and evaluating the spectral radius of the resulting next-generation matrix. The local stability of the DFE will be determined by analyzing the eigenvalues of the Jacobian matrix, while global stability will be established using appropriate Lyapunov functions and/or by using the properties of the Jacobian matrix of the infected compartments subsystem of the limiting system. The existence and uniqueness of endemic equilibria will be examined by analyzing their feasibility under model parameter constraints and by analyzing the dynamical behavior (monotonicity & boundedness) of the satelite system (i.e. the susbsystem of infected compartments). The persistence of LF is proved by proving that LF is weakly *F*-persistence (where *F* is a constructed function specific to our models and can be easily adapted to any similar model) and applying Theorem 4.5 in^25^.

#### Incorporating a superior rodent competitor into the full model

To assess how rodent control could potentially lead to Lassa fever virus elimination, we incorporate a hypothetical superior rodent competitor into the full model (2.1), aimed at reducing the population of the primary Lassa fever reservoir *Mastomys natalensis*. We assume this new species does not carry the Lassa fever virus. The competitor species will compete for the same resources, thereby decreasing the population and reproductive success of *Mastomys natalensis*. A similar method focusing on the introduction of a superior competitor has been examined in various studies, such as the approach proposed by Caut *et al*^26^..

The extended system that accounts for the competitor dynamics is obtained by replacing the rodent subsystem of the full model (2.1) with the subsystem (see Section S5 of SI). Since implementing this strategy can offer a sustainable and long-term reduction in Lassa fever incidence, the extended model (i.e., the full model (2.1) with the rodent sub-system replaced by the subsystem S5 in the SI is simulated to assess the impact of introducing a superior competitor on the dynamics of LF. Three scenarios will be explored and compared to the baseline case with no intervention: (i) competition with equal effects, $\zeta _c \text { ( defined as competition coefficient of the competitors) } = \zeta _r$ (competition coefficient of rodents); (ii) competition significantly reducing the population of *Mastomys natalensis* and disease prevalence; and (iii) interventions leading to the elimination of *Mastomys natalensis*. While the first scenario may maintain ecological balance, the second could destabilize it. In the elimination scenario, it is assumed the competitor will take over the ecological role of *Mastomys natalensis*.

### Numerical methods

This section outlines the methods for parameter estimation, uncertainty and sensitivity analysis, as well as the numerical simulation methods used.

#### Parameter estimation

The full model (2.1) has a total of 27 parameters. The numerical values of 12 of these parameters (referred to as fixed parameters) are known. That is, they can be derived directly from empirical studies or estimated from demographic or epidemiological data on LF^3,27–32^. These values were converted to epi-weeks. Detailed descriptions of the derivation or estimation procedure for these 12 parameters are provided in Section 4.1, while the numerical values of the parameters and their sources are summarized in Table 2.

The numerical values of the remaining 15 parameters are determined by fitting Model (2.1) to epidemiological data. Specifically, using the fixed parameter values and confirmed weekly LF case data from Nigeria^30^ spanning the period from December 29, 2019, to December 27, 2020 (and representing the LF epidemic episode of 2019-2020), we estimate these unknown parameters. One motivation for selecting this time-frame is the implementation of stringent COVID-19 measures (including testing), which contributed significantly to the identification of LF cases. The fitting process involves minimizing the sum of the squared differences between the data and the corresponding model-derived weekly cases quantity ($I_{ch}$) using the built-in MATLAB lsqcurvefit which implements the “Trust Region Interior Reflective” optimization algorithm^33^. This function requires the user to input the objective function, some initial parameters, $\Theta _0$ (chosen here based on the investigators experiences and information in literature), the time span of the observed data and a range for each parameter to be estimated. The objective function is defined as $f(\Theta ) = \sum _{i=1}^n \left( I_{ch}(t_i, \Theta ) - D_i \right) ^2$, where $D_i$ denotes the observed data at time $t_i$, $I_{ch}(t_i, \Theta )$ represents the model output based on the set of parameters $\Theta = (\Theta _1, \Theta _2)^T$ with $\Theta _1=(\beta _{ah}, \beta _{ch},\beta _{sh}, \beta _{rh},\beta _{rr},\beta _{vh},\beta _{vr},K, \rho _{sh},q, \alpha _{rv},\alpha _{sv},\alpha _{av},\alpha _{cv}, \mu _v)^T$ is the vector of unknown parameters, $\Theta _2=(\mu _h,\delta _{ch}, \delta _{sh},\mu _r,\tau ,\Lambda _h,\sigma _h,\gamma _{sh},\gamma _{ah},\gamma _{ch},\sigma _r,\Omega _r)^T$ is the vector of the fixed parameters, and $n$ is the number of data points. The model output ($I_{ch}$) is obtained using the inbuilt MATLAB ordinary differential equations solver (“ODE45”)^34^ to solve the system (2.1). The optional inputs are $3\times 10^{-14}$ for ”TolFun (tolerance of the function value)” and $8\times 10^{3}$ for MaxFunEvals. By iteratively refining $\Theta$, the fitting method identifies the parameter values that minimize $f(\Theta )$. Using $I_{ch}$ as a proxy for reported cases ensures alignment between the model’s predictions and observed data, enhancing both the reliability and interpretability of the parameter estimates. This approach provides a robust mechanism for refining parameter values, enabling the model to capture real-world dynamics effectively and offering a solid foundation for deeper analysis and intervention strategy development. The $95\%$ confidence intervals for the estimated parameters are computed using the bootstrapping method (also called bootstrap-percentile method), a robust resampling technique that evaluates the variability and uncertainty of parameter estimates^35,36^. This method is more convenient as we don’t known a specific distribution of each of the unknown parameters. In this approach, the residuals from the original model fit are resampled with replacement to generate multiple bootstrap samples, each having the same size as the original dataset. For each bootstrap sample, the model parameters are re-estimated using the same nonlinear least squares fitting procedure. This process is repeated many times (500 times for this study), producing a distribution of parameter estimates. The $95\%$ confidence intervals (CI) are then derived by identifying the 2.5*th* and 97.5*th* percentiles of the bootstrap distribution for each parameter. To compute the 2.5 th and 97.5 th percentiles of parameter distributions using the bootstrap method, the original dataset is repeatedly resampled with replacement to generate many bootstrap samples. For each sample, the parameter of interest is estimated, producing an empirical distribution. This distribution is then sorted, and the 2.5 th and 97.5 th percentiles are obtained by selecting the values at the corresponding positions to form the bounds of the 95% confidence interval, providing a non-parametric measure of uncertainty around the estimated parameters. This non-parametric method avoids normality assumptions, reliably quantifies uncertainty in parameter estimates, and reflects data variability, ensuring robust confidence intervals that enhance the credibility and predictive reliability of the model. To validate the fit, we followed the approach in^35^, using the fixed and estimated parameters to solve the model system (2.1), calculate residuals, test their random pattern using random test^37^ with the built-in MATLAB function ”runtest”. Additionally, the Root Mean Squared Error (RMSE), where $RMSE=\sqrt{\sum _{i=1}^{n}\frac{((\hat{I}_{ch})_i-D_i)^2}{n}}$, was computed and used as a statistical measure of the goodness of fit test^38^.

#### Uncertainty and sensitivity analysis

Uncertainty and sensitivity analysis are integral for disease models where parameter estimation is involved. These analyses help assess the robustness of model outputs to variations in parameters, aiding in decision-making and policy formulation. Local sensitivity analysis focuses on the impact of individual parameters on specific outputs, suitable when explicit output functions are known. In contrast, global sensitivity analysis evaluates the collective influence of multiple parameters simultaneously, crucial for complex models where outputs depend on numerous interacting factors. In particular, global sensitivity analysis techniques, such as the Latin Hypercube Sampling (LHS) approach based on sampling without replacement, together with Partial Rank Correlation Coefficients (PRCCs) or the extended Fourier Amplitude Sensitivity Test (eFAST), are pivotal for understanding the influence of parameters on model outputs^24^. PRCCs, which require a monotonic relationship between the output and parameter for valid results quantify correlations between parameter variations and output changes. The eFAST method, based on Fourier transformations, assesses parameter impact without the need for monotonic relationships, offering broader applicability in complex models. These techniques provide invaluable insights into parameter sensitivities, aiding in robust model calibration and uncertainty quantification for informed decision-making and effective disease management.

To perform a global uncertainty and sensitivity analysis of the model (2.1) using the LHS-PRCCs technique, each parameter is assumed to follow a uniform distribution and is divided into 5000 equal intervals. Parameter sets are then drawn without replacement to construct an LHS matrix. The model system is solved using these parameter sets, and PRCCs are calculated with the virus density in the environment (*V*) as the response function. These steps are implemented using the MATLAB package developed by Marino *et al.*^24^. The magnitude of the PRCCs is used to assess the correlation between the parameter and the output, where a PRCC of 0 indicates no correlation and a PRCC of 1 represents a strong correlation.

As some parameters may exhibit non-monotonic relationships with the outputs, we cannot conclude whether uncertainty or variability in these parameters will trigger significant uncertainty or variability in the response function. To assess the sensitivity of the response function to these parameters, we resort to the eFAST approach^24^. The Extended Fourier Amplitude Sensitivity Test (eFAST) is a variance-based global uncertainty and sensitivity analysis method that evaluates the influence of input variables on computational model outputs. Extending the traditional FAST, eFAST handles a larger number of inputs and interactions using a periodic sampling approach and Fourier transformation to decompose output variance into partial variances from different model parameters. The process involves model input sampling, where input variables of the model are assigned unique frequencies, and their values are varied sinusoidally over their ranges to generate a comprehensive set of samples; model evaluation, where the model is evaluated for each set of input samples, producing corresponding output values; Fourier transform, where the output values are transformed into the frequency domain using a Fourier transform (i.e., the output signal is decomposed into its constituent frequencies); Variance decomposition, where the total variance of the output is decomposed into contributions from each input variable and their interactions based on the amplitudes of the Fourier coefficients; and sensitivity indices calculation, where indices are calculated for each input variable, representing the proportion of the output variance that can be attributed to variations in that input to identify the most influential variables and their interactions. These first order sensitivity index and a total sensitivity index are metrics used to quantify the influence of input variables on the output of a model.

The first order sensitivity index (*Si*) measures the direct effect of a single input variable on the output, assuming all other variables are fixed. It is calculated by decomposing the variance of the model output attributed solely to the variation in the specific input variable, without considering interactions with other variables. A higher Si value indicates that the input variable has a significant direct influence on the output. The total order sensitivity index (*STi*) measures the overall effect of an input variable on the output, including both its direct effect and all interactions with other variables. It is derived by decomposing the variance of the model output that can be attributed to the input variable, considering both individual effects and higher-order interactions. A higher *STi* value suggests that the input variable, along with its interactions with other variables, plays a crucial role in influencing the output. It should be noted that Si focuses only on the direct impact of an individual variable, while *STi* encompasses the total impact, including interactions with other variables. Additionally, *Si* accounts for the contribution of a variable to output variance in isolation, whereas *STi* accounts for the contribution when considering all possible interactions. Furthermore, *Si* provides insight into the primary effects, whereas *STi* offers a comprehensive view by including interaction effects, making it useful for understanding the full extent of a variable’s influence. Here, eFAST was implemented with 5 search curves (resampling) and 65 samples *per* search curve and 28 input parameters (27 parameters from our model plus the dummy parameter). This parametrization of eFAST yields $5 \times 28 \times 65 = 9,100$ total sample points for this simulation which is enough to provide good results.

#### Numerical simulation methods

After parameterizing the model, it will be simulated using MATLAB’s built-in ode45 solver, an adaptive Runge-Kutta 4 th-5 th order method designed for non-stiff ODEs, to assess scenarios and evaluate dynamic behavior. The solver will numerically integrate the model over a defined time horizon, allowing analysis of time-dependent changes in key variables under varied parameter configurations. Scenarios include adjusting parameters such as transmission rates, recovery rates, and virus decay rates to evaluate the impacts of intervention strategies on model outcomes systematically. Also local sensitivity of the reproduction number to combinations of model parameters will be performed and the results will be presented using heatmaps.

## Analytical results

In this section, we state results related to the analyzes of three sub-systems derived from the model (2.1), along with the full model: 1) a rodent-free sub-system (Eqs. (S2.1) in the SI), 2) a rodent-virus sub-system (Eqs. (S3.1) in the SI), and 3) a human-rodent sub-system (Eqs. (S4.1) in the SI). Unless stated otherwise, all references to equations, tables, and figures in the SI will begin with the letter “S.” Starting with simpler models allows us to grasp key mechanisms, test assumptions, and ensure solid foundational insights before addressing the complexity of the full model. The analysis includes computing reproduction numbers and studying the existence and stability of equilibria, which help assess Lassa fever transmission potential, guide control strategies, and identify conditions for disease persistence or eradication.

### Analytical results of the rodent-free model

The rodent-free model (given by Eqs. (S2.1) in the SI) is obtained by setting all rodent related variables in the model (2.1) to zero, that is, $S_r = E_r = I_r = 0$.

The goal of this analysis is to understand whether the disease can persist when its main vector (the rodent) is removed from the transmission cycle leaving only human-to-human, human-to-environment, and environment-to-human transmission. The disease-free equilibrium of this model is $DFE_{hv} =(S_{h0}, E_{h0}, I_{ah0}, I_{sh0}, I_{ch0}, R_{h0}, V_{0}) =\left( \frac{\Lambda _h }{\mu _h },0,0,0,0,0,0\right)$. We use the next generation operator approach^22,23^ to compute the basic reproduction number of the model. Specifically, the matrices of new infections ($\mathcalligra{\scriptstyle F}$) and transitions ($\mathcalligra{\scriptstyle V}$) are given in (S2) of the SI. The basic reproduction number, i.e., the spectral radius of the matrix $\mathscr{F}\mathscr{V}^{-1}$ is

3.1 $$\begin{aligned} \mathcalligra{\scriptstyle R}_{0hv} = \frac{\mathcalligra{\scriptstyle R}_{0h} + \sqrt{(\mathcalligra{\scriptstyle R}_{0h})^2 + 4 \mathcalligra{\scriptstyle R}_{0v}}}{2}, \end{aligned}$$

$$\text { with, }\mathcalligra{\scriptstyle R}_{0h}=\frac{\sigma _{h}[\tau \beta _{ah} B_{s} B_{c} + (1 - \tau )B_{a}(\beta _{ sh }B_{c} + \beta _{ch} \rho _{sh})]}{ B_{a}B_{c}B_{e}B_{s}} ~ and ~ \mathcalligra{\scriptstyle R}_{0v} = \frac{\beta _{ vh }\Lambda _{h}\sigma _{h}}{\mu _{h}\mu _{v}}\frac{\tau \alpha _{av}B_{c}B_{s} + (1 - \tau )B_{a}(\alpha _{sv}B_{c} + \alpha _{cv}\rho _{sh})}{B_{a}B_{c}B_{e}B_{s}} .$$

The magnitude of the reproduction number determines the stability of the disease-free equilibrium of the rodent-free model (S2.1). Due to Theorem 2 in^39^, if $\mathcalligra{\scriptstyle R}_{0hv} < 1$, the disease-free equilibrium of the rodent-free model is locally asymptotically stable (i.e., $(S_{h}, E_{h}, I_{ah}, I_{sh}, I_{ch}, R_{h}, V) \rightarrow \left( \frac{\Lambda _h }{\mu _h },0,0,0,0,0,0\right)$ as $t\rightarrow \infty$ provided $R_{0hv} < 1$). Conversely, if $R_{0hv}> 1$, the disease-free equilibrium is unstable, indicating the potential for sustained disease transmission. This leads to the result:

#### Theorem 3.1

The disease-free equilibrium $DFE_{hv} =(S_{h0}, E_{h0}, I_{ah0}, I_{sh0}, I_{ch0}, R_{h0}, V_{0}) = \left( \frac{\Lambda _h }{\mu _h },0,0,0,0,0,0\right)$ of the rodent-free model given by Eqs. (S2.1) is locally asymptotically stable whenever $\mathcalligra{\scriptstyle R}_{0hv} < 1$ and unstable when $\mathcalligra{\scriptstyle R}_{0hv}> 1$.

In fact, it can be shown that the disease-free equilibrium ($DFE_{hv}$), of the rodent-free model, Eqs. (S2.1) is globally asymptotically stable whenever $\mathcalligra{\scriptstyle R}_{0hv} < 1$ and unstable when $\mathcalligra{\scriptstyle R}_{0hv}> 1$ (see Section S2 of the SI for the proof and other details). The epidemiological interpretation of this global stability result is that LF will be eliminated if $\mathcalligra{\scriptstyle R}_{0hv} < 1$ and the rodents are absent. Moreover, this result suggests that a backward bifurcation, characterized by the coexistence of stable disease-free and endemic equilibria when the reproduction number is less than one, is not feasible in this model. The next theorem establishes the existence and uniqueness of an endemic equilibrium solution to the rodent-free sub-system (S2.1). Furthermore, it can be verified that the rodent-free sub-system (S2.1) admits a single endemic equilibrium whenever $\mathcalligra{\scriptstyle R}_{0hv}> 1$. See S2 of SI for the proof.

### Analytical results of the rodent-virus sub-system

The rodent-virus sub-system (i.e., the model (2.1) without the human population) described by Eqs. (S3.1) in the SI is obtained by setting all variables related to the human population to zero (i.e., $S_{h} = E_h = I_{ah} = I_{sh} = I_{ch} = R_h = 0$). The disease-free equilibrium of this model is $DFE_{rv} = (S_{r0}, E_{r0}, I_{r0}, V_{0})=(\tilde{K}, 0, 0, 0)$ and the basic reproduction number computed using the Next Generation Operator approach (see Section S1.4 of the SI for details)

3.2 $$\begin{aligned} \mathcalligra{\scriptstyle R}_{0vr} = \frac{\mathcalligra{\scriptstyle R}_{0r} + \sqrt{\mathcalligra{\scriptstyle R}_{0r}^2 + 4 \mathcalligra{\scriptstyle R}_{0v}}}{2}, \end{aligned}$$

where $\displaystyle {\mathcalligra{\scriptstyle R}_{0r} = \frac{B_{r} \mu _{r} q +\beta _{ rr } \sigma _{r}}{ B_{r} \mu _{r}}}$ and $\displaystyle {\mathcalligra{\scriptstyle R}_{0v} = \frac{ \beta _{vr} \alpha _{r} \sigma _{r} \tilde{K}}{B_{r} \mu _{r} \mu _{v} } }$. The reproduction number ($\mathcalligra{\scriptstyle R}_{0vr}$) has been written in a form to emphasize the contributions of rodents ($\mathcalligra{\scriptstyle R}_{0r}$) and environmental contamination by the virus ($\mathcalligra{\scriptstyle R}_{0v}$). As in Section 3.1, it can be verified that the disease-free equilibrium ($DFE_{rv}$) is globally asymptotically stable when $\mathcalligra{\scriptstyle R}_{0vr} < 1$ and that $DFE_{rv}$ is unstable when $\mathcalligra{\scriptstyle R}_{0vr}> 1$ leading to the following result:

#### Theorem 3.2

The disease-free equilibrium $DFE_{rv} = (S_{r0}, E_{r0}, I_{r0}, V_{0})=(\tilde{K}, 0, 0, 0)$, of the rodent-virus sub-system (S3.1) in the SI is globally asymptotically stable when $\mathcalligra{\scriptstyle R}_{0vr} < 1$.

**Proof **The proof follows the steps outlined in Lemma S1.7 and Theorem S1.9. See Section S2 of the SI for details.$\square$ 

As in Section 3.1, it can be confirmed that the rodent-virus subsystem has a unique endemic equilibrium when $\mathcalligra{\scriptstyle R}_{0vr}> 1$.

### Analytical results of the model with no environmental contamination or transmission

The simplified model with no environmental contamination or transmission (i.e., the human-rodent sub-system) is obtained by setting the state variable *V* to zero in the model system (2.1). The disease-free equilibrium of the coupled human-rodent sub-system denoted by $DFE_{hr}$ is: $DFE_{hr} =(S_{h0}, E_{h0}, I_{ah0}, I_{sh0}, I_{ch0}, R_{h0}, S_{r0}, E_{r0}, I_{r0}) =\left( \frac{\Lambda _h }{\mu _h }, 0, 0, 0, 0, 0, \tilde{K}, 0 ,0\right)$. The basic reproduction number of the sub-system is

3.3 $$\begin{aligned} \mathcalligra{\scriptstyle R}_{0hr} = \max (\mathcalligra{\scriptstyle R}_{0h}, \mathcalligra{\scriptstyle R}_{0r}), \end{aligned}$$

where $\mathcalligra{\scriptstyle R}_{0h} = \displaystyle {\frac{\sigma _{h} \left[ \left( 1 - \tau \right) B_{a}\left( \beta _{ sh }B_{c} + \beta _{ ch }\rho _{ sh }\right) + \tau \beta _{ ah }B_{c} B_{s}\right] }{B_{s} B_{e} B_{a} B_{c}}}$ and $\displaystyle {\mathcalligra{\scriptstyle R}_{0r} = \frac{B_{r} \mu _{r} q + \beta _{ rr } \sigma _{r}}{B_{r} \mu _{r}}}$ (see Section S4 of the SI for details). It should be noted that $R_{0h}$ and $R_0^r$ have been written to emphasize the contributions of symptomatic, confirmed, and asymptomatic infectious human transmission, as well as regular and vertical rodent transmission on the reproduction number.

#### Theorem 3.3

The disease-free equilibrium ($DFE_{hr}$) of the model with no environment contamination is globally stable whenever its reproduction number ($\mathcalligra{\scriptstyle R}_{0hr}$) is less than one.

**Proof **See Section S4 of the SI.$\square$ 

### Analytical results of the full model

The disease-free equilibrium of the full model (Eqs. (2.1)) is $DFE =(S_{h0}, E_{h0}, I_{ah0}, I_{sh0}, I_{ch0}, R_{h0}, S_{r0}, E_{r0}, I_{r0}, V_{0}) = (\frac{\Lambda _h }{\mu _h }, 0, 0, 0, 0, 0, \tilde{K}, 0, 0, 0)$. This *DFE* is used along with the next generation operator approach^22^ to compute the basic reproduction number of the model (2.1), i.e., the spectral radius ($\rho (.)$) of the next generation matrix $\mathscr{F}\mathscr{V}^{-1}$. That is, $\mathcalligra{\scriptstyle R}_0 = \rho (\mathscr{F}\mathscr{V}^{-1})$. See Section S1 of the SI for details.

The following result, whose proof is provided in Section S1 of the SI establishes the global stability of the disease-free equilibrium, the existence and uniqueness of an endemic equilibrium to the model system, and the persistence of LF. It should be noted that the concept of persistence means that all the infected compartments remain bounded away from zero with any possible initial condition of the model (2.1).

#### Theorem 3.4

(Full Model results)

1. The DFE of the full model (2.1) is globally asymptotically stable whenever $\mathcalligra{\scriptstyle R}_0<1$.
2. If $\mathcalligra{\scriptstyle R}_0>1$, the full model (2.1) admits a unique endemic equilibrium.
3. If $\mathcalligra{\scriptstyle R}_0>1$, the disease is uniformly strongly persistent.

## Numerical results

### Estimated parameter values

#### Fixed (known) parameter values

Considering Nigeria’s average life expectancy at birth of approximately 54 years^27^ with a range of [51, 72] in 2020 (unless otherwise specified, range refers to the closed interval between the approximated low and upper value informed by epidemiological and demographic data, as well as prior studies), the natural mortality rate can be approximated as $\mu _h = (54.3\times 52)^{-1}=3.542\times 10^{-4}$ per week with range of $[2.6599, 3.7707]\times 10^{-4}$. To estimate the human recruitment rate ($\Lambda _h$), we leverage the model’s solution at the susceptible humans disease-free equilibrium ($\Lambda _h/\mu _h$). It should be note that at this equilibrium, the susceptible human population ($S_h$) aligns with the total population of Nigeria, estimated at approximately 208.2 million^28^. That is $\Lambda _h/\mu _h = 208,248,484$. Therefore, $\Lambda _h = 73753$ humans *per week*. The birth rate ($\Omega _r$) and the natural death rate ($\mu _{r}$) of rodent are extracted from^29^ and converted to the appropriate units. That is, $\Omega _r = 0.14815$ *per week* and $\mu _{r} = 0.04645$ *per week*. Since the human incubation period for LF ranges from 6 to 21 days^30^, we consider an average incubation period of $\frac{6 + 21}{2} = 13.5$ days. There, $\sigma _h = \frac{7}{13.5} \approx 0.5185$ *per week*, with a range of $\left[ \frac{7}{21} \approx 0.3333, \frac{7}{6} \approx 1.1667\right]$ *per* *week*. Based on research by Walker and colleagues^31^, *Mastomys natalensis* can start shedding the virus after 7 days from the moment they are infected. Using this information, we have $\sigma _r=7/7=1$ *per week*, with a range of [0.58, 2.00] *per week*. Additionally, the treatment duration for confirmed cases spans approximately 10 days, with therapy ranging from 8 to 14 days according to^30,32^. Consequently, the recovery rate for confirmed human cases is $\gamma _{ch} = \frac{7}{10} = 0.70$ *per week* with a range of $[\frac{7}{14} = 0.5000, \frac{7}{8} = 0.8750])$ *per week*. We assume that the recovery rates for symptomatic and asymptomatic infectious humans (i.e., ($\gamma _{sh}$) and $\gamma _{ah}$, respectively) are the same as the recovery rate for confirmed human cases. Furthermore, according to reports from the World Health Organization^3^ and the Nigerian Centre for Disease Control^30^, the overall case fatality rate (CFR) for LF vary between $0\%$ and $50\%$ per year, with an average of $20.8\%$ in 2020. The probability of mortality due to LF ($\delta _{ch}$) can be represented as $\delta _{ch}/(\mu _h+\gamma _{ch}+\delta _{ch})$. By substituting the known values of $\mu _h$ and $\gamma _{ch}$ into this formula and equating the resulting expression to the average CFR, we estimate the death rate among confirmed (hospitalized) cases as $\delta _{ch} = 0.1828$ *per week*. The death rate of symptomatic infectious humans is halved compared to hospitalized individuals, reflecting the majority of fatalities occurring within hospital settings. The values of these known (fixed) parameters are summarized in Table 2.

**Table 2:** Fixed (known) parameter values of the model (2.1). The index *h*, *r*, *ch*, *ah*, and *sh* is used to denote human, rodent, confirmed human case, asymptomatic infectious human, and symptomatic infectious human, respectively. With the exception of the incubation period and the disease-induced death rate derived from LF epidemiological data, all other parameters of the model were estimated using demographic data from Nigeria and data for rodents. The notations $w^{-1}$, $hw^{-1}$, and Ref. denote *per week*, human * per week*, and Reference, respectively.

**Parameter**	**Description(unit)**	** Value**	**Range**	**Ref.**
$\mu _{h}$	Natural death rate of humans ($w^{-1}$)	$35.42\times 10^{-5}$	$[26.60, \text 53.12]\times 10^{-5}$	^27^
$\delta _{ch}$	Human disease-induced mortality rate ($w^{-1}$)	$18.28\times 10^{-2}$	$[0.00, \text 70.04]\times 10^{-2}$	Cal$^*$
${\delta _{sh}}$	Human disease-induced mortality rate ($w^{-1}$)	$60.90\times 10^{-3}$	$[0.00, \text 35.02]\times 10^{-2}$	Cal$^*$
${\mu _{r}}$	Natural death rate of rodents ($w^{-1}$)	$46.45\times 10^{-3}$	$[23.22, \text 69.68] \times 10^{-3}$	^29^
$\tau$	Proportion of asymptomatic humans (unitless)	$80.00\times 10^{-2}$	$[70.00, \text 90.00]\times 10^{-2}$	^3^
$\Lambda _{h}$	Human recruitment rate ($hw^{-1}$)	73, 753	$[55,636.0, \text 78,545.0]$	Cal$^*$
${\sigma _h}$	Reciprocal of LF incubation period (w$^{-1}$)	$5.19\times 10^{-1}$	$[3.33, \text 11.67]\times 10^{-1}$	^30^
${\gamma _{sh}}$	Reciprocal of average $I_{sh}$ infectious period (w$^{-1}$)	$7.00\times 10^{-1}$	$[5.00,\text 8.75]\times 10^{-1}$	^30,32^
${\gamma _{ah}}$	Reciprocal of average $I_{ah}$ infectious period (w$^{-1}$)	$7.00\times 10^{-1}$	$[5.00,\text 8.75]\times 10^{-1}$	^30,32^
${\gamma _{ch}}$	Reciprocal of average $I_{ch}$ infectious period (w$^{-1}$)	$7.00\times 10^{-1}$	$[5.00,\text 8.75]\times 10^{-1}$	^30,32^
${\sigma _r}$	Reciprocal of incubation period for rodents (w$^{-1}$)	$1.00\times 10^{0}$	$[0.58, \text 2.00]\times 10^{0}$	^31^
${\Omega _{r}}$	Intrinsic growth rate of rodents (w$^{-1}$)	$14.82 \times 10^{-2}$	$[7.41, \text 22.23]\times 10^{-2}$	^29^

Calc$^{*}$: Calculated and described in the text above.

#### Unknown parameter values estimated by fitting the model to data

**Fig. 2:**
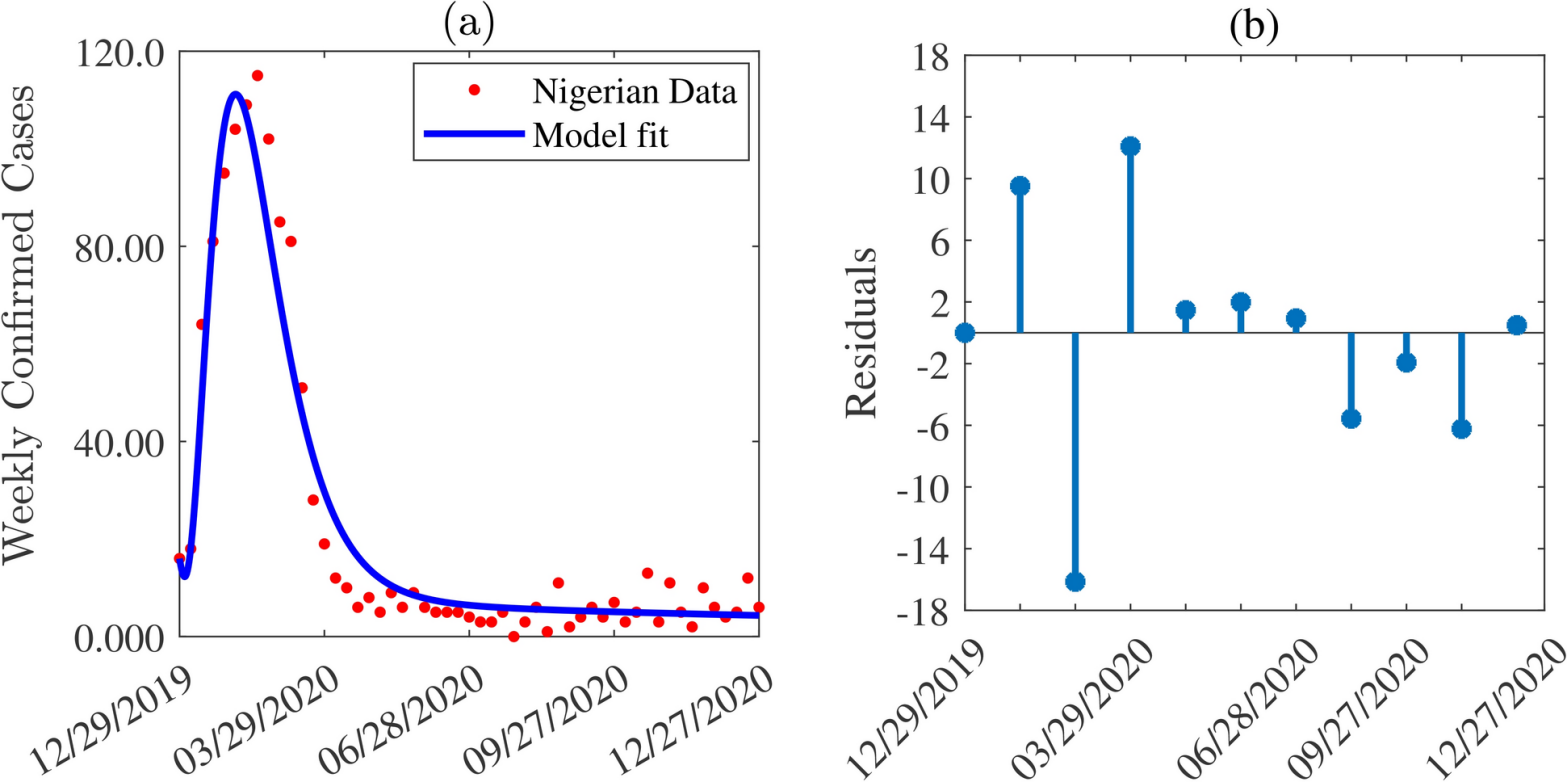
Full Model 2.1 fitting to data and validation. (**a**) Model fitting to observed weekly Lassa fever virus data (red dots) for the epidemic episode from December 29, 2019 to December 27, 2020. (**b**) Residuals (different between the observed data and the model fit) in function of time but displayed only at every 4 time points for convenience. The random behavior observed in the residuals confirmed the validity of the fit. Furthermore, the RMSE of 7.54 which is relatively small validated the model’s fit efficacy.

Table 3 presents the estimated parameters with their $95\%$ confidence intervals, while Figure 2(a) illustrates the data (red circles), the fitted model (blue curve). The residuals trend depicted in Figure 2 (b) with a random pattern confirmed (since p-value = 0.1608 > 5%) by random test^37^ run using MATLAB (2021a) function ”runtest”, justified that our fit is good^35^. An in-depth analysis of the estimated parameters reveals that asymptomatic infectious individuals play a crucial role in transmitting Lassa Fever virus. Specifically, they account for approximately $98.39\%$ of human-to-human transmissions and $\approx 37\%$ of the overall transmission is attributed to asymptomatic cases. The first percentage is calculated as the ratio of the asymptomatic transmission rate ($\beta _{ah}$) to the total human transmission rates ($\beta _{ah} + \beta _{ch} + {\beta _{sh}}$), while the second represents the fraction of the asymptomatic transmission rate relative to the sum of all transmission rates in Table 3. Notably, rodent-to-rodent and asymptomatic transmissions together account for over $94\%$ of the total transmission. Additionally, asymptomatic infectious individuals shed viruses to the environment at a rate that is $\approx 0.9$ times the shedding rate of symptomatic individuals and $\approx 10$ times the shedding rate of confirmed individuals (comparing the shedding rate, $\alpha _{av}$ with the shedding rates $\alpha _{sv}$ and $\alpha _{cv}$ in Table 3). Furthermore, the reproduction number obtained using the estimated and fixed parameter values is below unity (specifically, $\mathcalligra{\scriptstyle R}_0 = 0.5$) for the rodent-free model indicating that the disease can be contained without the rodent transmission pathway. However, with rodent transmission, the reproduction number can surge to as high as 6, exacerbating the spread of the disease.

**Table 3:** Estimated parameters and $95\%$ confidence intervals (CIs), where LB and UB denote the lower and upper bounds. The root mean square error (RMSE) of the fit is 7.54 which is relatively small demonstrating the validity of estimated parameters. The notations $v(rw)^{-1}$, $v(hw)^{-1}$, and $(vw)^{-1}$ denote virus *per rodent per week*, virus *per human per week*, and *per virus per week*, respectively.

**Parameters**	** Description (unit)**	$95\%$ CI LB	** Value**	$95\%$ CI UB
$\rho _{sh}$	Human LF detection rate ($w^{-1}$)	$1.40\times 10^{-5}$	$1.41\times 10^{-5}$	$3.95\times 10^{-5}$
*q*	Infectious proportion of rodent births (unitless)	$1.38\times 10^{-2}$	$2.23\times 10^{-1}$	$6.69\times 10^{-1}$
$\alpha _{rv}$	Rodent LF shedding rate (v(rw)$^{-1}$)	$0.95\times 10^{-5}$	$1.00\times 10^{-5}$	$1.24\times 10^{-5}$
$\alpha _{sv}$	Human ($I_{sh}$) LF shedding rate (v(hw)$^{-1}$)	$1.00\times 10^{-6}$	$1.104\times 10^{-6}$	$6.03\times 10^{-6}$
$\alpha _{av}$	Human ($I_{ah}$) LF shedding rate (v(hw)$^{-1}$)	$1.00\times 10^{-6}$	$1.01\times 10^{-6}$	$2.86\times 10^{-6}$
$\alpha _{cv}$	Human ($I_{ch}$) LF shedding rate (v(hw)$^{-1}$)	$0.88\times 10^{-7}$	$1.01\times 10^{-7}$	$1.28\times 10^{-3}$
$\mu _v$	Decay rate of the virus in the environment (w$^{-1}$)	$5.50\times 10^{-1}$	$6.11\times 10^{-1}$	$1.45\times 10^{0}$
${\beta _{ah}}$	Human-to-human transmission rate by $I_{ah}$ (w$^{-1}$)	$9.12\times 10^{-2}$	$1.16\times 10^{-1}$	$3.44\times 10^{-1}$
$\beta _{ch}$	Human-to-human transmission rate by $I_{ch}$ (w$^{-1}$)	$8.25\times 10^{-5}$	$3.49\times 10^{-4}$	$9.86\times 10^{-3}$
$\beta _{sh}$	Human-to-human transmission rate by $I_{sh}$ (w$^{-1}$)	$1.00\times 10^{-3}$	$1.55\times 10^{-3}$	$5.00\times 10^{-2}$
$\beta _{rh}$	Rodent-to-human transmission rate (w$^{-1}$)	$4.98\times 10^{-5}$	$7.48\times 10^{-4}$	$1.44\times 10^{-3}$
$\beta _{rr}$	Rodent-to-rodent Transmission rate (w$^{-1}$)	$4.03\times 10^{-3}$	$1.80\times 10^{-1}$	$6.78\times 10^{-1}$
$\beta _{vh}$	Environment-to-human transmission rate ($(vw)^{-1}$)	$1.00\times 10^{-4}$	$2.74\times 10^{-4}$	$3.30\times 10^{-4}$
$\beta _{vr}$	Environment-to-rodent transmission rate ($(vw)^{-1}$)	$1.50\times 10^{-2}$	$1.53\times 10^{-2}$	$1.77\times 10^{-2}$
*K*	Rodent carrying capacity (rodent)	1, 700, 281	3, 625, 971	4, 725, 655

### Uncertainty and sensitivity analysis results

**Fig. 3:**
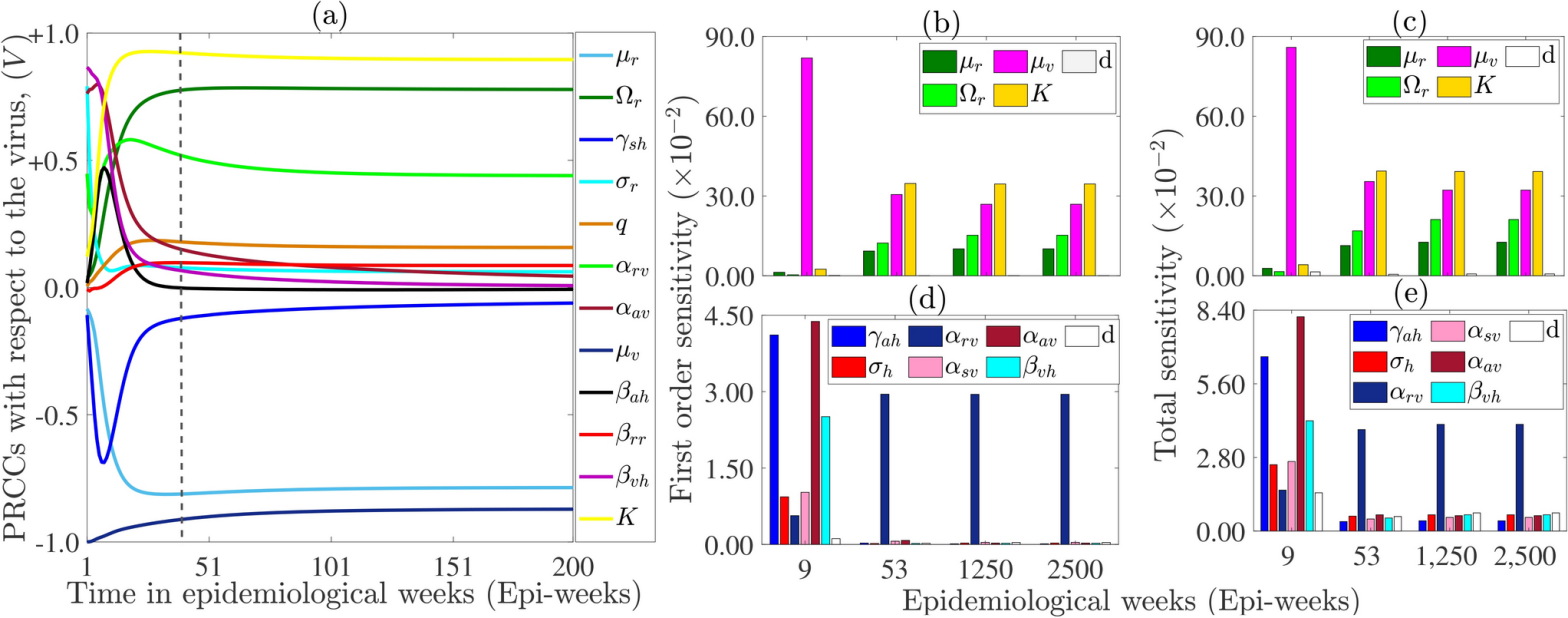
(**a**) Uncertainty and sensitivity analysis of the model (2.1) using LHS-PRCC. Only PRCCs of parameters exhibiting a monotonic relationship with the output are displayed. (**b**)-(**e**) eFAST sensitivity indices of the model parameters with respect to the amount of virus in environment: (**b**) and (**d**) First order sensitivity indices of the virus in the environment (*V*) with respect to model parameters at different times in Epidemiological weeks. (**c**) and (**e**) Total order sensitivity indices of *V* with respect to model parameters. Only the parameters with significant indices (p-value $<0.05$) are displayed. That is the index of the parameter is significantly different (p-value $<0.01$) from the index of the dummy (black white bar). The time $t = 9$ ($t = 2500$) epidemiological weeks correspond to the approximate time when the number of confirmed cases peaks (the time the endemic equilibrium is attained). The parameter values and their corresponding ranges are presented in Tables 2 and 3.

A global uncertainty and sensitivity analysis, using techniques LHS and PRCCs eFAST, is essential for studying complex disease models, such as the Lassa fever virus model in this study, with numerous parameters. This method helps quantify the uncertainty and sensitivity of the model outputs beyond the calibration period, where the bootstrapping technique described in Section 4.1 is not applicable. In addition, these methods are critical for identifying key drivers of infection and mortality, quantifying the impacts of parameters, and ensuring robust predictions despite uncertainties. Additionally, they are useful for refining interventions, guiding data collection, and improving public health efforts to control and mitigate the burden of diseases. Results of a global uncertainty and sensitivity analysis of the full model (2.1) carried out using these approaches are depicted in Figure 3, with

PRCCs of parameters with the most significant impact on the virus in the environment (*V*) depicted in Figure 3 (a). The analysis indicates that uncertainty and variability in parameters such as the rodent carrying capacity (*K*), the intrinsic birth rate of rodents ($\Omega _r$), the rate at which rodents shed virus to the environment ($\alpha _{rv}$), and the vertical transmission probability (*q*) will lead to significant uncertainty and variability in *V*, each showing a positive correlation with *V* near or at the disease endemic equilibrium, DEE, the right hand side of the vertical line). Conversely, variations in parameters like the rodent natural death rate ($\mu _r$),the recovery rate of symptomatic infectious ($\gamma _{sh}$), and decay rate of the LF in the environment ($\mu _v$) will lead to significant variations in *V* and each of these parameters is negatively correlated with *V* near DEE. Increasing the rodent birth rate results in a higher population density, providing more potential hosts for the Lassa fever virus (LF). With more rodents present, the likelihood of LF transmission and persistence in the environment increases, consequently leading to a rise in LF levels in the environment. While *V* is not very sensitive to *K* initially, it becomes highly sensitive to *K* at a later time (i.e., small changes or variations in *K* lead to significant changes or variations in *V* over time). This is because initially, when rodent numbers are low compared to *K*, their influence on Lassa fever virus (LF) transmission may be minimal due to ample resources. However, as rodent populations grow, resource competition escalates, potentially compromising immunity and increasing LF susceptibility. Consequently, minor variations in carrying capacity later become significantly influential, amplifying LF transmission sensitivity to this parameter.

Some parameters, particularly those not shown in Figure 3 (a), display non-monotonic relationships with the outputs, making it difficult to determine their influence on the uncertainty or variability of the response function. To evaluate their sensitivity, we apply the eFAST method^24^. Results of the sensitivity analysis using eFAST (depicted in Figure 3 (b)-(e)) highlight key contributors to virus surface levels, particularly during the peak period (around epidemiological week 9). Among these, the virus decay rate ($\mu _v$) explains approximately $86\%$ of the variance (the magenta bar on Fig. 3(d), followed by the rodent carrying capacity (*K*) at approximately $4\%$. Additionally, the infectious asymptomatic human virus shedding rate ($\alpha _{ah}$) accounts for around $8\%$ of the variance at the peak (dark red bar in Figure 3 (e)), while the recovery rate of the asymptomatic persons ($\gamma _{ah}$) explains approximately $7\%$ (light blue bar in Figure 3 (e)). Although the rodent shedding rate ($\alpha _{rv}$) does not affect the variance of *V* at peak as does the transmission rate from environment to human ($\beta _{vh})$, this parameters affect significantly the variance of *V* on the long time (the variance is $\approx 4\%$ from epidemiological week from 53). A similar trend is observed for the first order sensitivity index (Figure 3 (b) and (d). The epidemiological implication of these results is that the most influential Lassa fever virus control measures for significantly reducing peak magnitudes (of incidence or death) can be assessed through the infectious rodent shedding rate ($\alpha _{rv}$), the virus decay rate ($\mu _v$), the rodent death rate ($\mu _{r}$) and the asymptomatic infectious human virus shedding rate ($\alpha _{av}$). Moreover, the parameters $\Omega _r$, $\alpha _{rv}$,$\mu _v$,$\mu _r$, and *K* are identified as potential drivers for disease extinction. Comparing the *Si* (Figure 3 (b)-(d)) and the STi (Figure 3 (c) and (e)) indicates that the model (2.1) is not additive, since the majority of the parameters have higher STi than Si. According to Marino et al. (2008), if $STi> Si$, the model is additive. This non-additive nature implies that an effective Lassa fever virus control strategy must consider interactions among the system’s parameters that contribute to disease persistence. Additionally, besides the parameters identified previously as contributors to variability in virus quantity on surfaces, the virus shedding rate by confirmed infectious humans ($\alpha _{cv}$) also plays a major role. It should be noted that although uncertainty and variability in the parameters *q* and $\beta _{sv}$ appeared not be able to introduce significant uncertainty and variability in *V* from the computed PRCCs, they exert significant influence on *V* from eFAST sensitivity analysis.

### Lassa fever virus prevalence in Nigeria

**Fig. 4:**
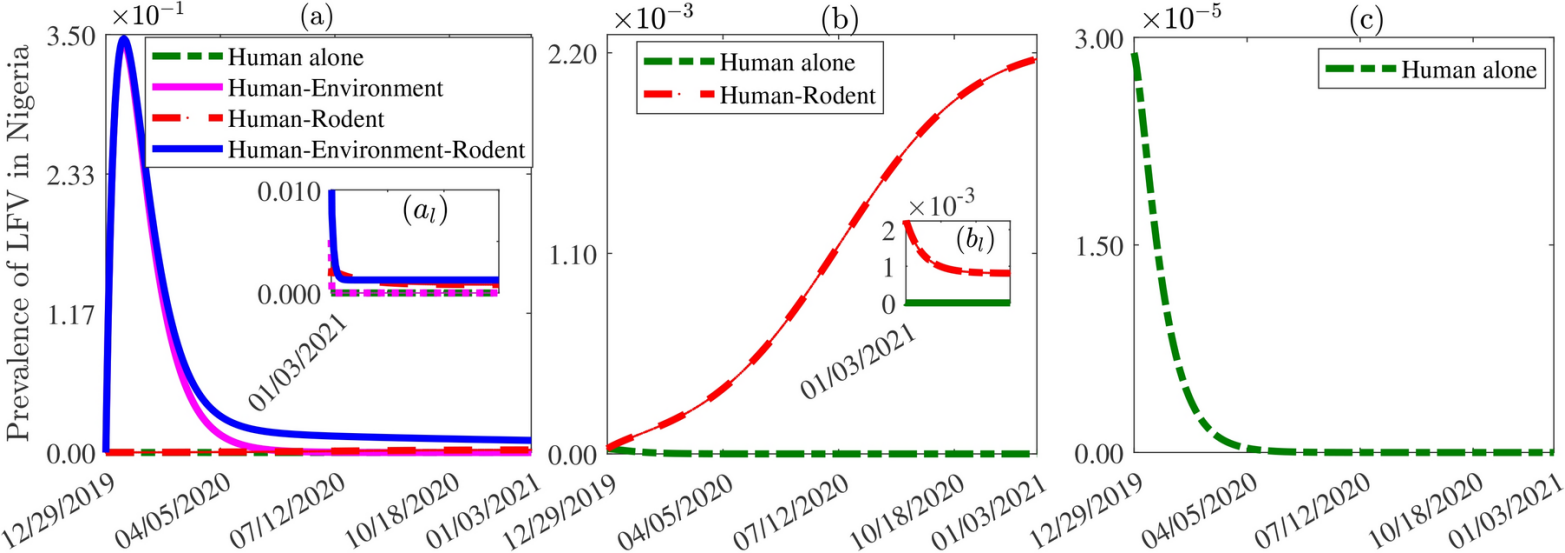
Human prevalence of Lassa fever virus disease in Nigeria for (**a**) the full model (blue curve), the rodent-free or human-virus sub-system (magenta curve), the human-rodent sub-system (dashed red curve), and the rodent- and environmental contamination-free or the human-to-human transmission model (dashed green curve). (**b**) Zoom-in view of the Lassa fever virus prevalence curves for the human-rodent subsystem (dashed red curve) and the human-to-human sub-system (dashed green curve). (**c**) Further zoom-in view of the Lassa fever virus prevalence curve for the human-to-human sub-system. The parameter values used for the simulations are provided in Tables 2 and 3.

We used the fixed and estimated parameters in Tables 2-3 to estimate the prevalence of LF, applying the full model (blue curve), rodent-free or human-virus sub-system (magenta curve), human-rodent sub-system (dashed red curve), and the rodent- and environmental contamination-free or the human-to-human transmission model (dashed green curve). The prevalence of Lassa fever virus in humans is the ratio of the total infected human population to the total human population (i.e., $(E_h+I_{ah}+ I_{ch}+I_{sh})/N_h$). The results obtained and presented in Figure 4 (a) show that the presence of all transmission pathways considered in the full model (2.1) leads to higher disease prevalence (blue curve), while the human-to-human transmission pathway alone leads to the lowest prevalence (dashed green curve). In particular, the prevalence peak size of the human-to-human transmission pathway is $\approx 99.99\%$ less than that of the combined pathways from the full model, while at equilibrium, the human-to-human transmission pathway leads to an $\approx 100\%$ lower prevalence compared to the full model (2.1) (comparing the blue and dashed green curves in Figure 4 (a)). Hence, in the scenario where disease transmission occurs solely within the human population, without rodent or environmental transmission (dashed green curve), the disease dies out within a few days. Similarly, the prevalence peak of the human-virus (human-rodent) sub-system alone is approximately $\approx 1\%$ ($\approx 99.38\%$) lower than the full model, and leading to an $\approx 100\%$ ($\approx 12\%$) reduction in prevalence at equilibrium (comparing the blue with the magenta and the dashed red curves in Figure 4 (a)). As for the case of only human-to-human transmission, the disease eventually dies out for the human-virus sub-system indicating that the disease cannot persist without rodent involvement in its transmission. A detailed view of Lassa fever virus prevalence for the human-rodent subsystem (red curve) and the human-to-human subsystem (green curve) is presented in Figure 4 (b). Additionally, Figure 4 (c) provides an enhanced magnification of the prevalence curve for the human-to-human subsystem.

### Lassa fever interventions measures

Lassa fever control strategies have been studied extensively, focusing primarily on treatments like Ribavirin for hospitalized patients and measures such as contact tracing and quarantine of confirmed cases. However, our research uniquely explores additional measures, including the use of gloves/personal protection, regular home disinfection, control of rodent birth and mortality rates, rodent repellent usage, and rodent-proof containers. These measures, which have not been analyzed in depth previously through a mathematical framework, are likely to be effective in managing and potentially reducing the spread of LF in human populations. By implementing these practices, the disease can be more effectively controlled, offering a comprehensive approach to LF management. Simulations of the model (2.1) will be carried out using the parameter values in Tables 2-3, unless otherwise stated to assess the impact of these control and mitigation measures on LF and to estimate the associated implementation costs. For each control measure studied in this section, three levels of implementation–low ($25\%$), moderate ($50\%$), and high ($75\%$)–are considered.

#### Assessing the impact of surface disinfection on Lassa fever virus dynamics

**Fig. 5:**
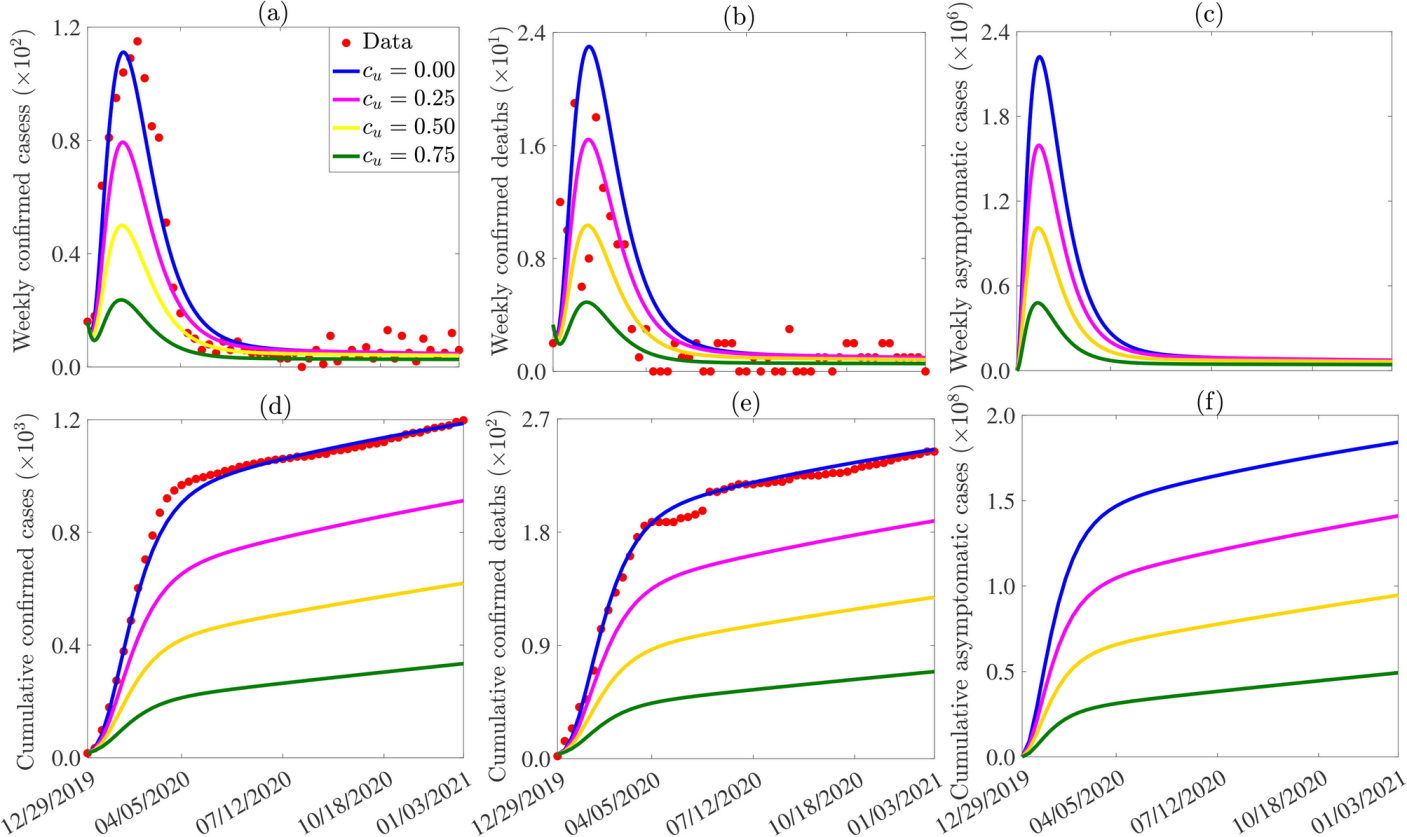
Simulations of the model (2.1) to assess the impact of surface disinfection ($c_u$) on the weekly (**a**) confirmed cases, (**b**) confirmed deaths, (**c**) asymptomatic infectious cases and the cumulative number of (**d**) confirmed cases, (**e**) confirmed deaths, (**f**) asymptomatic infectious cases. The initial conditions used for the simulations are presented in Table S3 of SI and the other parameters used for the simulations are presented in Tables 2-3.

Surface disinfection has been proven to be effective in mitigating the spread of infectious diseases by reducing transmission risk and accelerating virus decay rates. The model (2.1) is simulated to evaluate the impact of disinfectant usage on LF propagation. Disinfection reduces both environment-to-human and environment-to-rodent transmission rates, and increases the virus decay rate in the environment. In the model, disinfection modifies the LF transmission rates from the environment to humans ($\beta _{vh}$) and rodents ($\beta _{vr}$) by multiplying them by $1 - c_u$, and the virus decay rate ($\mu _v$) by $1 + c_u$, where $0 \le c_u \le 1$ represents the percentage reduction in transmission or increase in virus decay. Three scenarios are considered: low disinfection ($c_u = 25\%$, i.e., a $25\%$ reduction in LF transmission or increase in virus mortality), moderate disinfection ($c_u = 50\%$), and high disinfection ($c_u = 75\%$). Results of the simulations depicted in Figure 5 suggest that increasing levels of disinfection, from low ($25\%$) to high ($75\%$), result in progressively greater reductions in weekly peak numbers of confirmed cases, deaths, and asymptomatic infectious individuals compared to the baseline scenario, accompanied by noticeable delays in peak times. In particular, at a low disinfection level, comparative analysis with the baseline scenario reveals a reduction of $28.6\%$ in weekly confirmed cases, $28.8\%$ in deaths, and $27.4\%$ in asymptomatic infectious individuals at their respective peaks, as evidenced by the magenta curve contrasted against the blue curve in Figure 5 (a)-(c). Similarly, under moderate disinfection conditions, a reduction of $55.1\%$, $55.0\%$, and $55.26\%$ in weekly confirmed cases, deaths, and asymptomatic infectious individuals at their peaks is observed compared to the baseline scenario, depicted by the yellow curve in contrast to the blue curve in Figure 5 (a)-(c). Moreover, at high levels of disinfection, there is a notable reduction of $78.69\%$, $78.76\%$, and $78.41\%$ in weekly confirmed cases, deaths, and asymptomatic infectious individuals at their peaks relative to the baseline scenario, as shown by the green curve against the blue curve in Figure 5 (a)-(c). Similar trends are observed for cumulative cases (Figure 5 (d)-(f)). In particular, at high disinfection levels, a $71.85\%$, $71.85\%$, and $71.28\%$ reduction in the cumulative number of confirmed cases, deaths, and asymptomatic infectious cases, respectively, compared to the baseline cumulative number of confirmed cases would have been recorded by January, 2021 (comparing the green and blue curves in Figure 5 (d)-(f)).

#### Assessing the impact of gloves and protective clothing usage on Lassa fever virus dynamics

**Fig. 6:**
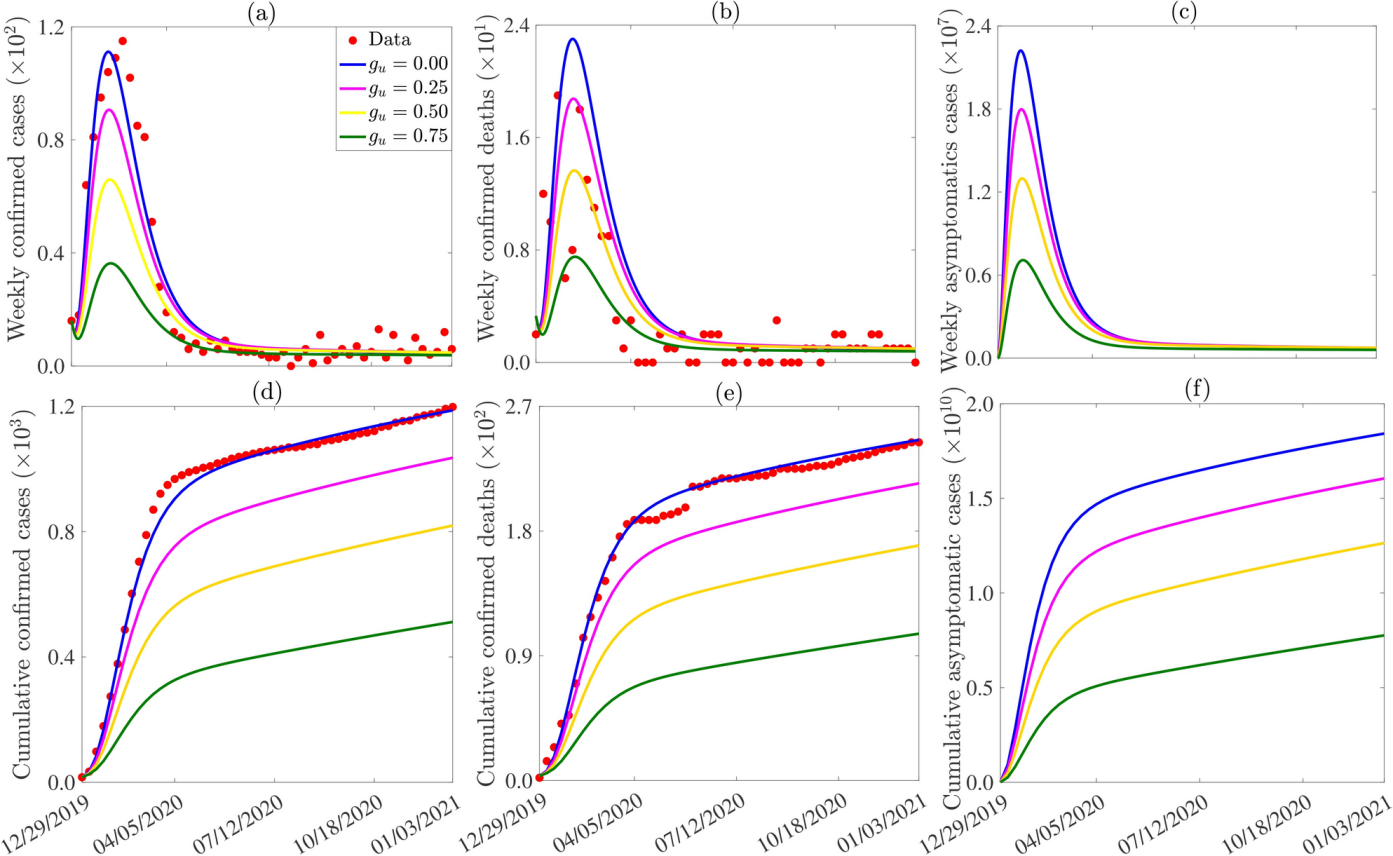
Simulations of the model (2.1) to assess the impact of glove ($g_u$) on the weekly (**a**) confirmed cases, (**b**) deaths, (**c**) asymptomatic infectious cases and the cumulative number of (**d**) confirmed cases, (**e**) deaths, (**f**) asymptomatic infectious cases. The initial conditions used for the simulations are presented in Table 3 of SI and the other parameters used for the simulations are presented in Tables 2-3.

Appropriate glove and protective clothing usage play a crucial role in controlling Lassa fever virus transmission by minimizing direct contact with infectious materials, thus reducing the risk of infection among healthcare workers and individuals in high-risk environments. Beyond mitigating transmission in hospital settings, glove protective clothing usage also serve to prevent human contamination when handling objects that rodents have interacted with, potentially leaving behind bodily fluids such as urine. The model (2.1) is simulated to assess the impact of gloves and protective clothing usage on the number of confirmed cases, deaths, and asymptomatic infectious individuals. This measure is implemented in the model through the rate $(1 - g_u)\beta _{vh}$, where ($0 \le g_u \le 1$) is a reduction in the transmission rate from the environment to humans ($\beta _{vh}$). The results derived from the simulations (illustrated in Figure 6) indicate discernible patterns across varying levels of disinfection. At a low level of glove or protective clothing adoption, a reduction of $18.5\%$, $18.5\%$, and $19.1\%$ in the weekly counts of confirmed cases, deaths, and asymptomatic infectious individuals at their peaks, respectively, is observed compared to the baseline scenario (as evident from the comparison between the magenta and blue curves in Figure 6 (a)-(c)). Concurrently, there is a respective delay of $4.1\%$, $5.1\%$, and $2.7\%$ in the time taken for the weekly counts of confirmed cases, deaths, and asymptomatic infectious individuals to reach their peaks. At a moderate level of glove or protective clothing employment, a reduction of $40.7\%$, $40.7\%$, and $41.6\%$ in the weekly counts of confirmed cases, deaths, and asymptomatic infectious individuals at their peaks is recorded compared to the baseline scenario (as depicted by the yellow curves against the blue curves in Figure 6 (a)-(c)). Correspondingly, there is a delay of $5.8\%$, $8\%$, and $4.8\%$ in the time taken for the weekly counts of confirmed cases, deaths, and asymptomatic infectious individuals to peak. Furthermore, at a high level of glove or protective clothing usage, a reduction of $67.3\%$, $67.3\%$, and $68.1\%$ in the weekly counts of confirmed cases, deaths, and asymptomatic infectious individuals at their peaks is noted compared to the baseline scenario (as illustrated by the green curves against the blue curves in Figure 6 (a)-(c)). Likewise, there is a delay of $8.1\%$, $11\%$, and $7.5\%$ in the time taken for the weekly counts of confirmed cases, deaths, and asymptomatic infectious individuals to peak. Similar results are observed with the cumulative cases (Figure 6 (d)-(f)). It’s worth noting that the reductions achieved through glove or protective clothing usage are less significant compared to those attained through disinfection measures.

### Assessing the impact of measures that reduce infectious human populations

**Fig. 7:**
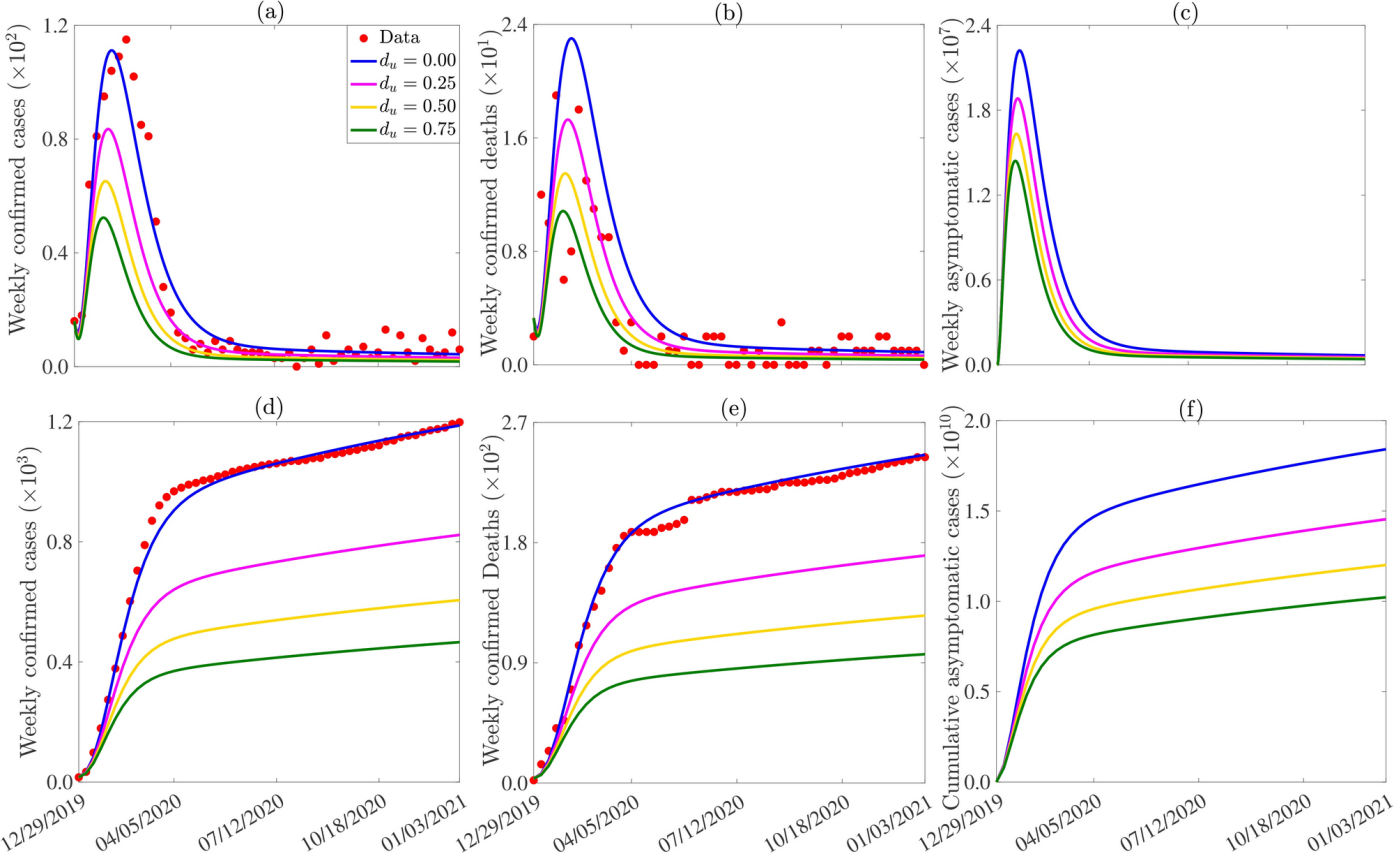
Simulations of the model (2.1) to assess the impact of LF control measures that reduce the infectious human population such as treatment on the weekly (**a**) confirmed LF cases, (**b**) LF deaths, (**c**) asymptomatic infectious LF cases, and the cumulative (**d**) confirmed LF cases, (**e**) LF deaths, (**f**) asymptomatic infectious LF cases. The initial conditions used for the simulations are presented in Table S3 of SI and the other parameters used for the simulations are presented in Tables 2-3.

The model (2.1) is simulated to assess the impact of LF control measures on reducing infectious human populations. Treatment of symptomatic ($I_{sh}$) and confirmed ($I_{ch}$) LF cases with the drug Ribavirin effectively reduces the confirmed infectious human population. Additionally, the hypothetical scenario where asymptomatic infectious humans ($I_{ah}$) are identified and treated is assessed. This assessment involves an increase in the recovery rate of treated individuals, assuming treatment accelerates recovery. Specifically, the recovery rates ($\gamma _{jh}, j \in \{a, c, s\}$) are multiplied by the factor $1 + d_u$, where $d_u$ is a treatment modification factor. Results of the simulations suggest that treating only confirmed cases leads to minor reductions in case numbers. More significant reductions are achieved when symptomatic, asymptomatic, and confirmed infectious humans are treated. For instance, a $25\%$ increase in recovery rates results in a $25.2\%$, $22.1\%$, and $17.9\%$ reduction in the baseline peak number of weekly confirmed cases, deaths, and asymptomatic infectious cases, respectively (comparing the blue and magenta curves in Figure 7 (a)-(c)). A $75\%$ increase in recovery rates leads to $52.9\%$, $51.3\%$, and $35.5\%$ reductions in these same metrics, respectively (comparing the blue and green curves in Figure 7 (a)-(c)). Similar trends are observed for the cumulative cases. Specifically, a $25\%$ increase in recovery rates results in $30.7\%$, $30.7\%$, and $21\%$ reductions in the baseline number of cumulative confirmed cases, deaths, and asymptomatic infectious cases, respectively (comparing the blue and magenta curves in Figure 7 (d)-(f)), while a $75\%$ increase results in $60.8\%$, $60.8\%$, and $44.5\%$ reductions in these metrics, respectively (comparing the blue and green curves in Figure 7 (d)-(f)).

### Assessing the impact of rodent control on Lassa fever virus dynamics

**Fig. 8:**
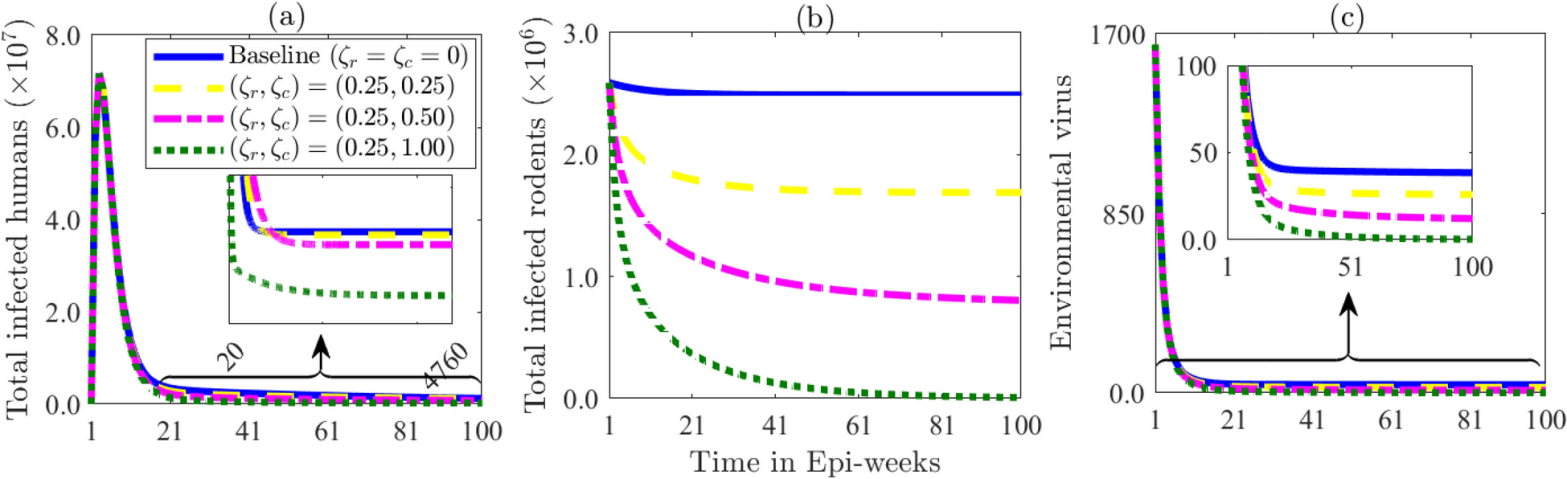
Impact of introducing a superior competitor of the host rodent on the total (**a**) infectious human population, (**b**) infectious rodent population, and (**c**) the virus on surface. The baseline curve is obtained by simulating the full model (in the absence of the competitor). The simulation time span was extended up to 100 epi-weeks to ensure the system reaches steady state, although the model was fitted only to data for the first 52 epidemiological weeks. For this plot, $\omega _c = 0.30$, $K_c = K_r$ and all the other parameters are as presented in Tables 2-3.

The simulation results depicted in Figure 8 show that introducing a competitor will lead to a significant reduction in the total number of infectious humans. Specifically, under equal competition effects (e.g., when $\zeta _c = \zeta _r = 0.25$), there is a $7\%$ reduction in the baseline infectious human population and a $32\%$ reduction in the baseline infectious rodent population by epidemiological week 100 (comparing the blue and yellow curves in Figure 8 (a) and (b)). When the competitor’s coefficient exceeds that of the host rodent (i.e., if $\zeta _c> \zeta _r$), greater reductions occur. For instance, with $\zeta _c = 0.5$ and $\zeta _r = 0.25$, the reductions in both the baseline infectious human and rodent populations are $36\%$ and $68\%$, respectively, by epidemiological week 100 (comparing the blue and magenta curves in Figure 8 (a) and (b)). Additionally, for $\zeta _c = 1.0$ and $\zeta _r = 0.25$, a $81.7\%$ and a $99.7\%$ reduction in the baseline infectious human and rodent populations is observed by epidemiological week 100 (comparing the blue and green curves in Figure 8 (a) and (b)). Comparable, but slightly lower reductions are observed for the virus in the environment (Figure 8 (c)).

### Assessing the impact of important model parameters on the basic reproduction number

**Fig. 9:**
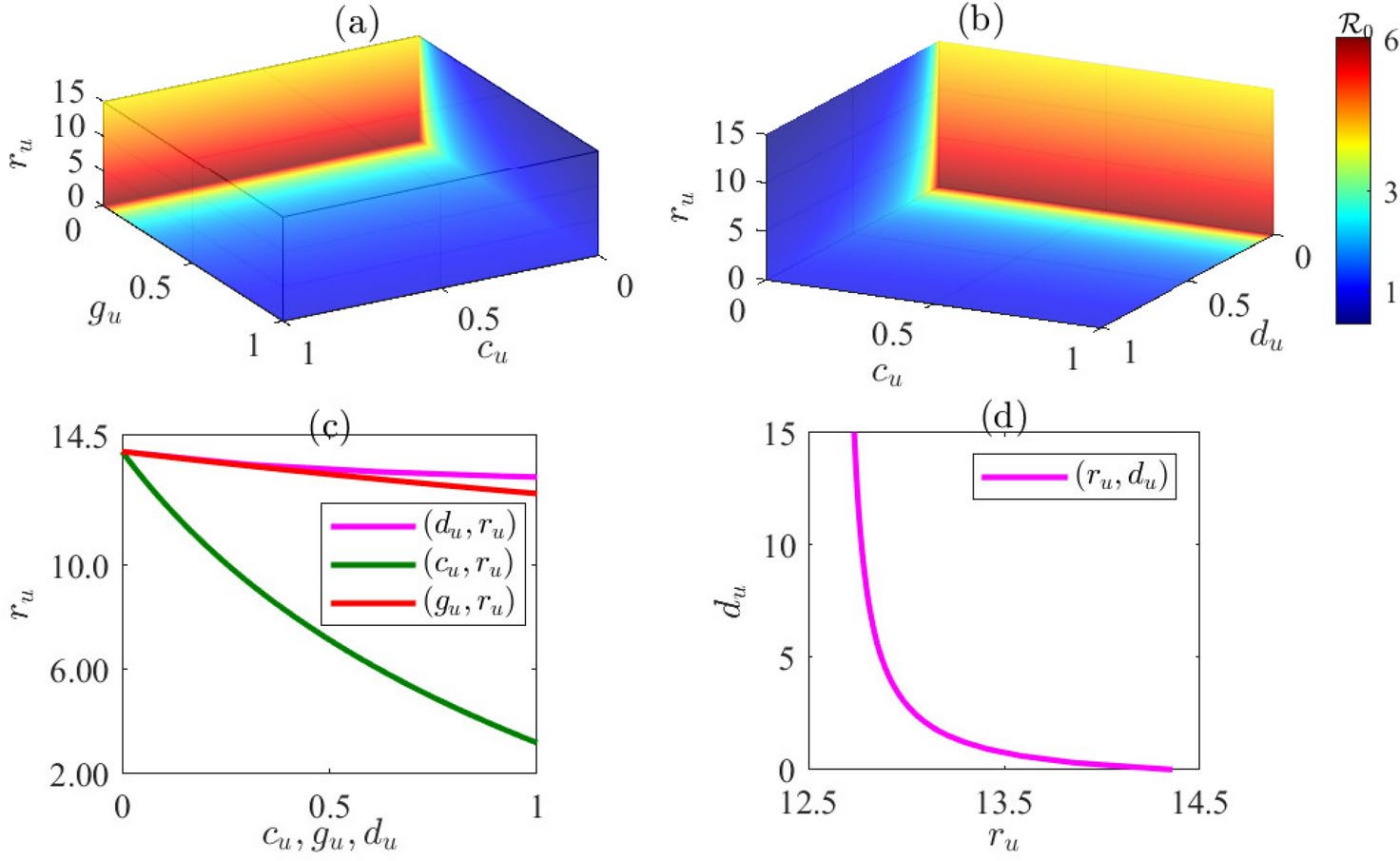
Heatmap of the reproduction number ($\mathcalligra{\scriptstyle R}_0$) of the full model (2.1) as a function of (**a**) the Lassa virus vector control measure ($r_u$), glove or protective cloth usage ($g_u$), and disinfection usage ($c_u$) and (**b**) $r_u$, $c_u$, and the treatment rate of infectious humans ($d_u$). Two-dimensional control strategies (i.e., various combinations of two measures that yield $\mathcalligra{\scriptstyle R}_0<$1) : (**c**) $r_u$ against $c_u$, $d_u$, and $g_u$; and (**d**) $r_u$ against $d_u$ for extended $d_u$. The parameter values used are provided in Tables 2 and 3.

Important LF control and mitigation measures include environmental disinfection, antiviral treatment, the use of personal protective equipment (PPE), e.g., gloves and protective clothing, and rodent control. Each of these measures can be assessed through specific model models. While each measure independently aids in reducing transmission, addressing the multi-faceted transmission pathways of the disease through a combination of two or more control and mitigation measures can enhance the overall effectiveness of the control campaign significantly. Hence, we assess the impact of deploying multiple LF control measures and the combination of various implementation levels required to reduce the reproduction number to or below one. Specifically, two- and three-dimensional heatmaps (Figure 9 (a)-(b) and Figure 9 (c)-(d), respectively) are plotted to assess the combined impact of various model parameters including parameters through which control measures can be assessed such as the rodent reduction factor ($r_u$), glove or protective cloth usage compliance coefficient ($g_u$), treatment of infected individuals ($d_u$) and disinfection usage ($c_u$) on the reproduction number ($R_0$) of the full model (2.1). The results indicate that when $r_u = 7.0$ and $g_u = 0.6$, a $c_u$ value of 0.55 is needed to reduce $R_0$ below one, while increasing $r_u$ and $g_u$ to 9.0 and 0.8, respectively, lowers the required $c_u$ to 0.3 (Figure 9 (a)). However, for $r_u$ and $g_u$ below 3.0 and 0.3, reducing $R_0$ below one is unattainable even with $c_u = 100\%$. Similarly, if $r_u = 7.0$ and $d_u = 5.0$, a $c_u$ value of 0.5 is required, but with higher $r_u$ and $d_u$ values of 9.0 and 7.0 a lower $c_u$ of 0.3 is sufficient (Figure 9 (b)). When $r_u$ and $d_u$ are below 3.0, achieving $\mathcalligra{\scriptstyle R}_0 < 1$ is also infeasible even with maximum $c_u$. Therefore, bringing $\mathcalligra{\scriptstyle R}_0$ below unity is feasible primarily with disinfection, especially at significantly high application levels (e.g., $c_u \approx 98\%$) with $r_u$ level as low as 3.3. However, none of the other single measures considered here leads to a similar result. But combining any other measure with disinfection can effectively lower the reproduction number below one, potentially requiring a lower level of disinfection depending on the type and level of the additional measure. In particular combining a measure that involves reducing the rodent population (implemented through the parameter $r_u$) with disinfection is more effective in controlling the disease as depicted by the dark green curve in Figure 9 (c). However, combining other measures (e.g., $r_u$ and $d_u$ depicted by the magenta curve in Figure 9 (c)) without disinfection, is less effective in controlling the disease. Figure 9 (d) illustrates that treatment alone is insufficient to reduce the reproduction number below one, thereby preventing LF containment. However, when treatment is combined with rodent reduction strategies, such as the use of rodenticides, the reproduction number can be decreased below one. Specifically, with a treatment parameter of $d_u = 5.22$, a rodent reduction factor of $r_u = 12.87$ is necessary to achieve this. Conversely, if $d_u$ is reduced to 1.22 then a rodent reduction factor of at least 13.3 is required to lower the reproduction number below one. Additionally, Figures 9 (c) and (d) indicate that the disease can be effectively contained through measures focused solely on rodent population reduction. Notably, if the rodent population modification factor reaches $\approx 14.4$, rodent reduction alone suffices to contain the disease without the need for supplementary measures.

## Discussion and caveats

### Discussion

In this study, a mathematical model framework for Lassa fever dynamics that incorporates overlooked factors like vertical transmission in rodents, surface contamination, and asymptomatic transmission is developed and used to assess the impact of these factors and various control and mitigation measures on disease spread in Nigeria. The framework consists of a full model Eqs. (2.1) together with sub-systems to identify and quantify the impact of various transmission routes. Analysis of each of the sub-systems, as well as the full system suggests that there is a globally stable disease-free equilibrium when the reproduction number of the system under study is less than one and a unique endemic equilibrium when the reproduction number is greater than one. A globally stable disease-free equilibrium implies that, regardless of initial Lassa fever virus infection levels, the disease will eventually be eliminated from the population as long as the model’s conditions are met. In public health terms, this suggests that the implemented control measures (e.g., environmental disinfection, antiviral treatment, the use of personal protective equipment, rodent control, etc.) are sufficient to stop disease transmission, preventing any long-term presence of the infection in the community. Once the disease is eradicated, no further outbreaks will occur as long as external factors, like reintroduction of the disease, are controlled. Hence, achieving global stability is a crucial goal in disease control strategies, as it guarantees that public health interventions will result in the eventual elimination of the disease for the entire population^40,41^. Conversely, the existence of a unique endemic equilibrium in a disease when $\mathcalligra{\scriptstyle R}_0> 1$ implies that the disease will persist in the population at a constant level, rather than being eliminated, even in the long term. This suggests that, under the given conditions, the implemented control measures are insufficient to completely eradicate the disease. Instead, the infection will stabilize at a certain prevalence, which is determined by factors such as transmission rates, recovery rates, and population structure. This has significant implications for public health, indicating that additional or more effective interventions are necessary to reduce or eliminate the disease^23^.

The reproduction numbers of the sub-systems are computed analytically, while that of the full system is computed numerically using a combination of known parameters and unknown parameters estimated by fitting the full model system to Lassa fever virus data from Nigeria. The model parametrization suggests that asymptomatic individuals are key drivers of Lassa fever virus transmission, responsible for about $98.39\%$ of human-to-human spread and shedding viruses at significantly higher rates than symptomatic and confirmed cases. Also, this process suggests that the basic reproduction number for the rodent-free model is below unity, suggesting the disease can be controlled without rodent transmission. However, when rodent transmission is considered, the reproduction number of the full model (2.1) can rise to 6, greatly increasing the risk of disease spread. This value ($\mathcalligra{\scriptstyle R}_0 = 6$) is about three times greater than estimated reproduction number values in the literature^13,42,43^. This difference arises from our model’s inclusion of multiple additional transmission pathways, such as asymptomatic transmission, vertical transmission in rodents, and environmental contamination compared to previous studies (e.g^14^.,). This, together with a few other studies including that in^44^, which have estimated the basic reproduction number for Lassa fever to be as high as 6 or more, suggests that some previous studies may have underestimated the true burden of the disease. These high values highlight the potential for widespread transmission, particularly in endemic regions where most cases ($80\%$) are asymptomatic. It should be noted that model-based estimates may be lower due to the exclusion of certain transmission routes, such as rodent vertical transmission in rodents, asymptomatic carriers, and environmental transmission, underscoring the complexity of accurately quantifying basic reproduction number.

Global sensitivity analysis results show that variability in parameters like the rodent carrying capacity, birth rate, vertical transmission probability, and virus shedding rate positively correlates with environmental virus levels, leading to greater uncertainty in virus amounts in the environment. In contrast, parameters such as rodent death rate, recovery rate of symptomatic individuals, and virus decay rate negatively correlate with virus levels. An increased rodent birth rate leads to higher rodent populations, raising the risk of Lassa fever transmission and persistence. While environmental virus levels are initially less sensitive to rodent carrying capacity, they become highly sensitive as rodent populations grow, due to increased competition and reduced immunity. As a result, small changes in carrying capacity can significantly affect virus transmission at later stages.

The impact of various control and mitigation measures for LF were explored through heatmaps and simulations of the full model (2.1). These measures include antiviral treatment, environmental disinfection, the use of personal protective equipment and the use of rodenticides. Antiviral treatment targets individuals who are symptomatic or have confirmed infections, helping to reduce the infectious period and overall viral load. However, treating only confirmed cases has a limited impact on the broader transmission dynamics, as asymptomatic carriers and unconfirmed cases continue to spread the virus. Environmental disinfection involves cleaning surfaces and environments where the virus may persist, reducing the risk of transmission from contaminated surfaces to humans. This measure is crucial in areas with high human-animal interaction, such as markets and rural communities where LF is prevalent. Personal protective equipment, including gloves, masks, and protective clothing, minimizes direct contact with the virus, especially for healthcare workers and individuals in high-risk areas. They are essential for preventing transmission during interactions with infected individuals and contaminated environments. Rodenticides either cause internal bleeding by preventing blood clotting (anticoagulants) or target the nervous and respiratory systems through different mechanisms (non-anticoagulants), ensuring the rodent dies gradually after ingestion, minimizing bait aversion. The study suggests that there is a threshold PPE compliance level above which PPE usage becomes the better option. The study indicates that a threshold rodent population level exists, above which controlling LF solely through vector reduction measures is unfeasible, while below this threshold, such control becomes feasible. Furthermore, the study shows that Lassa fever virus control measures are most effective when implemented in combination rather than as single interventions, as combined interventions can address multiple transmission pathways and reduce the overall burden of the disease. For instance, promoting personal hygiene, proper rodent control, safe food storage, and community-wide education on prevention can synergistically reduce the risk of infection^45^. Also, treating both symptomatic and asymptomatic cases, in conjunction with rigorous environmental disinfection, can drastically reduce the reproduction number. Additionally, studies have shown that combining rodent control with environmental sanitation and personal protective measures, like wearing gloves and masks, is more effective at reducing Lassa fever transmission than any single approach alone^45–47^. This combined approach disrupts multiple transmission pathways, thereby reducing the overall infection rate more effectively than any single measure could achieve. Additionally, integrating PPE usage with treatment and disinfection protocols provides comprehensive protection for both healthcare workers and the general population. This multifaceted strategy not only mitigates immediate transmission but also helps in preventing outbreaks from escalating, making it a critical approach in the effective control and eventual eradication of LF. In summary, the integration of antiviral treatment, environmental disinfection, and PPE use forms a robust defense against LF. This strategy addresses various transmission routes and stages of the infection, underscoring the importance of a holistic approach to managing and controlling infectious diseases such as LF.

The results of the study indicate that the full transmission model exhibits the highest prevalence of the disease, while the human-to-human transmission scenario alone results in the lowest prevalence, highlighting significant variability in the transmission dynamics between different subsystems. Additionally, subsystems with a rodent component lead to higher prevalence than those without the rodent component. This underscores the critical importance of addressing rodent involvement to achieve effective control of Lassa fever transmission. Hence, the impact of a hypothetical control measure involving competitor rodents is assessed. Specifically, we introduce a hypothetical non-virus-carrying rodent competitor into the model to reduce the population of the primary Lassa fever reservoir, *Mastomys natalensis*, by competing for shared resources. Simulation results of this extended model system indicate that introducing a competitor rodent species significantly reduces infectious human and rodent populations, with reductions of up to $81.7\%$ and $99.7\%$, respectively, under strong competition by week 100. These findings align with previous studies, which also show that competitive displacement of the primary rodent reservoir can lead to substantial declines in disease transmission^48,49^.

Introducing a competing rodent species to control Mastomys natalensis, the primary reservoir of Lassa fever, is a theoretically viable but complex strategy with ecological and epidemiological risks. This approach, known as biological control, has been explored for vector-borne diseases but requires careful assessment of feasibility and unintended consequences^50^. While introducing competitors could reduce *Mastomys* *natalensis*  populations through resource competition, there is a risk of ecological imbalance, unforeseen interspecies interactions, and potential pathogen spillover^51^. Additionally, the introduced species may fail to establish or could become invasive, causing broader ecosystem disruptions^52^. Given these risks, ecological modeling and field trials are necessary before implementation to ensure safety and effectiveness.

In summary, the study demonstrates that incorporating vertical transmission in rodents, surface contamination, and asymptomatic transmission significantly amplifies Lassa fever spread, with asymptomatic carriers human-to-human transmission and environmental contamination increasing disease persistence. In particular, accounting for vertical transmission of Lassa fever virus in rodents is crucial because it sustains the infection within rodent populations independent of external transmission, increasing disease persistence, amplifying spillover risks to humans, and complicating control efforts by making rodent population reduction alone insufficient for eradication. Control and mitigation strategies, including antiviral treatment, personal protective equipment, environmental disinfection, and rodent population control, effectively reduce transmission, with combined interventions proving more impactful than single measures. Notably, rodent-free models indicate that Lassa fever can be controlled without rodent transmission, but incorporating rodents increases the basic reproduction number significantly. Additionally, introducing a competing rodent species to suppress Mastomys natalensis populations shows promising reductions in disease prevalence, reinforcing the importance of multi-faceted intervention strategies.

### Limitations

This study has several limitations. The seasonality of Lassa fever transmission was not incorporated, as it was outside the article’s scope, but this is unlikely to affect the results since only a single wave was considered. A constant human recruitment rate was assumed, which, although a common practice in mathematical modeling, may limit the findings given the exponential growth of the African and Nigerian populations; a framework is being developed to address this. Although sexual transmission of the Lassa fever virus is suspected, it was excluded due to insufficient evidence. The environmental model is complex due to high inhomogeneity, but our approach captures the key aspects needed for this study. Data uncertainties may affect the results, though the validity tools applied ensure their reliability. For consistency, we fitted the full model to data and applied the estimated parameters uniformly across both the full and submodels to establish a reliable baseline. However, we recognize that alternative model structures could yield different parameter estimates, potentially influencing the dynamics. For example, the transmission dynamics between rodents and humans may differ depending on whether environmental factors are considered. Although introducing a competitor rodent species yields plausible results, to our knowledge, no current efforts use this approach for Lassa fever control. However, this “what if” scenario is theoretical and, like transgenic mosquito research for malaria control^53^, can inspire new investigations. Similarly, high disinfection levels and widespread protective clothing, though unlikely, serve to explore potential interventions that could drive future research. While we used a simple yet reliable bootstrap technique for constructing confidence intervals, a more robust bootstrap scheme such as that in^35^ might provide greater reliability. Finally, the model is deterministic, which prevents it from capturing the stochastic nature of individual interactions in human and rodent populations or the environmental factors that influence transmission.

## Conclusion

The study identifies asymptomatic individuals as the primary drivers of Lassa fever transmission and emphasizes that incorporating additional transmission pathways such as vertical transmission and environmental contamination leads to a higher estimated reproduction number, potentially exposing underestimation of the disease’s burden in prior research. The study’s results reveal that transmission dynamics vary significantly, with the full transmission model showing the highest prevalence and rodent-involved sub-systems leading to greater disease prevalence, underscoring the necessity of addressing rodent involvement for effective Lassa fever control and improved public health outcomes. It suggests that effective control measures can facilitate disease containment and that maintaining rodent populations below a critical threshold is essential for control of LF through vector reduction measures. Moreover, combined strategies-including antiviral treatment, environmental disinfection, and personal protective equipment-outperform single interventions, which yield only minor infection rate reductions. The study shows that vertical transmission in rodents sustains Lassa fever within rodent populations, complicating eradication efforts and amplifying spillover risks. A multifaceted approach, particularly one that addresses symptomatic, asymptomatic, and confirmed cases alongside robust disinfection and protective measures, and rodent population management significantly lowers the reproduction number and enhances containment. Global sensitivity analysis further indicates that rising rodent birth rates increase transmission risk, with the impact of rodent carrying capacity becoming more pronounced as populations grow. Finally, the introduction of a competitor rodent species can lead to substantial reductions in both infectious human and rodent populations, showcasing a promising strategy for controlling Lassa fever transmission. In conclusion, this study highlights the necessity of integrated and multifaceted strategies for effectively managing Lassa fever, including paying attention to asymptomatic cases, comprehensive treatment and disinfection approaches, and competitive displacement measures to minimize transmission and enhance public health outcomes.

## Electronic supplementary material

Below is the link to the electronic supplementary material.

Supplementary Material 1

## Data Availability

All data used in this study is freely accessible in Table S4 of the SI or can be obtained upon request from the corresponding author (Email: hemahobeaugtaboe@ufl.edu).
