# Supplementary material for: Revealing hidden drivers of Lassa fever through a model-informed approach for reproducing and predicting disease dynamics and guiding control strategies
Source: Sci Rep. 2025 Sep 30;15:33786. doi: 10.1038/s41598-025-01176-y (PMC12485196; doi:10.1038/s41598-025-01176-y)
Supplement: Supplementary file 1 — Supplementary Material 1 [file 41598_2025_1176_MOESM1_ESM.pdf]

Revealing hidden drivers of Lassa fever through a model-informed approach for reproducing and predicting disease dynamics and guiding control strategies

Hemaho B. Taboe [1,2,✉], **Sergei S. Pilyugin**[1], **and Calistus N. Ngonghala**[1,3,4,5]

[1]Department of Mathematics, University of Florida, Gainesville, FL 32611, USA.
[2]Laboratoire de Biomathématiques et d'Estimations Forestières, University of Abomey-Calavi, Cotonou, Bénin.
[3]Emerging Pathogens Institute, University of Florida, Gainesville, FL 32610, USA.
[4]Harvard Radcliffe Institute of Advanced Studies, 10 Garden St., Cambridge, MA 02138, USA.
[5]Harvard Medical School, Boston, MA 02115, USA.,
✉Corresponding author, email: hemahobeaugtaboe@ufl.edu

ABSTRACT

See the main paper.

Supplementary Information (SI)

S1 Methods

Table S1. Definitions of the state variables used in the model.

S_h	Susceptible humans: Individuals who have not contracted LFV.
E_h	Exposed humans: Infected individuals, who are not infectious yet.
I_{ah}	Asymptomatic humans: Individuals who do not exhibit clinical symptoms of LFV after the incubation period.
I_{sh}	Symptomatic infectious humans: Individuals who exhibit clinical symptoms of LFV after the incubation period.
I_{ch}	Confirmed or reported cases of LFV, including those under treatment.
R_h	Recovered humans: Infectious humans, who have recovered from infection.
S_r	Susceptible rodents: Rodents that have not contracted LFV yet.
E_r	Exposed rodents: Rodents that have contracted LFV, but are not infectious yet.
I_r	Infectious rodents: Infected rodents that can transmit the disease.
V	Density of the virus in the environment (i.e., on contaminated surfaces, materials, equipment, etc).

Table S2. Brief description of the parameters used in the model. The subsripts s, a, and a denote symptomatic, asymptomatic, and confirmed LFV cases, respectively.

Parameter	Description	Parameter	Description
Λ_h	Human birth rate	Ω_r	Intrinsic growth rate of rodents
μ_h	Natural death rate of humans	μ_r	Natural death rate of rodents
$1/\sigma_h$	LFV incubation period in humans	$1/\sigma_r$	LFV incubation period in rodents
$\alpha_{jv}, j = s,a,c$	Human LFV shedding rate	α_{rv}	Rodent LFV shedding rate
$\delta_{jh}, j = s,c$	Human disease-induced mortality rate	q	Infectious proportion of rodent births
$\beta_{jh}, j = s,a,c$	Human-to-human transmission rate	β_{rr}	Rodent-to-rodent Transmission rate
β_{rh}	Rodent-to-human transmission rate	K	Rodent carrying capacity
ρ_{sh}	Human LFV detection rate	μ_v	Decay rate of the virus in the environment
$1/\gamma_{jh}, j = s,a,c$	Average jth human infectious period	τ	Proportion of asymptomatic humans

Using the model schematics together with the model, variable and parameter descriptions (Tables S1-S2), we obtain the following system of equations for the dynamics of the disease within humans, rodents, and the environment:

S1 The full model

$$
\begin{aligned}
\dot{S_h} &= \Lambda_h - \left(\frac{\beta_{ah}I_{ah}+\beta_{sh}I_{sh}+\beta_{ch}I_{ch}}{N_h} + \frac{\beta_{rh}I_r}{N_r} + \beta_{vh}V\right) S_h - \mu_h S_h \\
\dot{E_h} &= \left(\frac{\beta_{ah}I_{ah}+\beta_{sh}I_{sh}+\beta_{ch}I_{ch}}{N_h} + \frac{\beta_{rh}I_r}{N_r} + \beta_{vh}V\right) S_h - (\mu_h+\sigma_h)E_h, \\
\dot{I_{ah}} &= \tau\,\sigma_h E_h - (\mu_h+\gamma_{ah})\,I_{ah}, \\
\dot{I_{sh}} &= (1-\tau)\,\sigma_h E_h - (\mu_h+\gamma_{sh}+\rho_{sh}+\delta_{sh})I_{sh}, \\
\dot{I_{ch}} &= \rho_{sh}I_{sh} - (\mu_h+\gamma_{ch}+\delta_{ch})I_{ch}, \\
\dot{R_h} &= \gamma_{ch}I_{ch}+\gamma_{sh}I_{sh}+\gamma_{ah}I_{ah} - \mu_h R_h, \\
\dot{S_r} &= \Omega_r(1-\frac{N_r}{K})\,[S_r+E_r+(1-q)I_r] - \left(\frac{\beta_{rr}I_r}{N_r}+\beta_{vr}V\right) S_r - \mu_r S_r, \\
\dot{E_r} &= \left(\frac{\beta_{rr}I_r}{N_r}+\beta_{vr}V\right) S_r - (\sigma_r+\mu_r)E_r, \\
\dot{I_r} &= q\Omega_r\left(1-\frac{N_r}{K}\right) I_r + \sigma_r E_r - \mu_r I_r, \\
\dot{V} &= \alpha_{rv}I_r + \alpha_{av}I_{ah} + \alpha_{sv}I_{sh} + \alpha_{cv}I_{ch} - \mu_v V.
\end{aligned}
\tag{S1.1}
$$

The dynamics of the total human population is governed by the equation $\dot{N_h} = \Lambda_h - \mu_h N_h - \delta_{sh}I_{sh} - \delta_{ch}I_{ch} \leq \Lambda_h - \mu_h N_h$. Hence, the size of the total human population is bounded above by the inequality $N_h(t) \leq \frac{\Lambda_h}{\mu_h} + \left(N_h(0) - \frac{\Lambda_h}{\mu_h}\right) e^{-\mu_h t}$, where $N_h(0)$ is the initial total human population. It should be noted that $\lim_{t\to\infty} N_h(t) \leq \frac{\Lambda_h}{\mu_h}$. The total rodent population is described by the equation $\dot{N_r} = \Omega_r(1-\frac{N_r}{K})N_r - \mu_r N_r = \tilde{\Omega}_r(1-\frac{N_r}{\tilde{K}})N_r$, which has the explicit solution $N_r(t) = \frac{\tilde{K}N_r(0)}{N_r(0)+[\tilde{K}-N_r(0)]e^{-\tilde{\Omega}_r t}}$, where $N_r(0)$ is the initial total rodent population and $N_r(t) \to \tilde{K}$ as $t\to\infty$. Hence, $\mathbb{D} = \mathbb{D}_h \times \mathbb{D}_r \times \mathbb{D}_v \subseteq \mathbb{R}^6_+ \times \mathbb{R}^3_+ \times \mathbb{R}_+$, where $\mathbb{D}_h = \left\{(S_h,E_h,I_{ah},I_{sh},I_{ch},R_h) \in \mathbb{R}^6_+ : 0 \leq S_h,E_h,I_{ah},I_{sh},I_{ch},R_h \leq \frac{\Lambda_h}{\mu_h}\right\}$, $\mathbb{D}_r = \left\{(S_r,E_r,I_r) \in \mathbb{R}^3_+ : 0 \leq S_r,E_r,I_r \leq \tilde{K}\right\}$, and $\mathbb{D}_v = \left\{V \in \mathbb{R}_+ : 0 \leq V \leq \frac{\alpha_{rv}\tilde{K}}{\mu_v} + (\alpha_{av}+\alpha_{sv}+\alpha_{cv})\frac{\Lambda_h}{\mu_h\mu_v}\right\}$ is a positively-invariant region (i.e., solutions that start in $\mathbb{D}$ remain in $\mathbb{D}$, $\forall t > 0$) which attracts all positive solutions of the model. Analysis of the model will be performed within this feasible region.

Disease-free equilibrium and the basic reproduction number of the full model

At a state where there is no disease in both rodent and human population (the Disease Free equilibrium), the solution to the system (S1.1) is denoted by DFE is: $DFE = (S_{h0},E_{h0},I_{ah0},I_{sh0},I_{ch0},R_{h0},S_{r0},E_{r0},I_{r0},V_0) = (\frac{\Lambda_h}{\mu_h},0,0,0,0,0,\frac{K(\Omega_r-\mu_r)}{\Omega_r},0,0,0)$. The basic reproduction number is calculated at DFE using the next generation matrix approach[2]. Here, we let F be the matrix of new infections (at DFE) and let V be the transition matrix. Then from system S1.1 we obtain :

$$
\mathscr{F} = \begin{pmatrix}
0 & \beta_{ah} & \beta_{sh} & \beta_{ch} & 0 & \frac{\beta_{rh}\Lambda_h\Omega_r}{\mu_h(\Omega_r-\mu_r)K} & \frac{\beta_{vh}\Lambda_h}{\mu_h} \\
0 & 0 & 0 & 0 & 0 & 0 & 0 \\
0 & 0 & 0 & 0 & 0 & 0 & 0 \\
0 & 0 & 0 & 0 & 0 & 0 & 0 \\
0 & 0 & 0 & 0 & 0 & \beta_{rr} & \frac{\beta_{vr}(\Omega_r-\mu_r)K}{\Omega_r} \\
0 & 0 & 0 & 0 & 0 & q\mu_r & 0 \\
0 & \alpha_{av} & \alpha_{sv} & \alpha_{cv} & 0 & \alpha_{rv} & 0
\end{pmatrix}, \quad
\mathscr{V} = \begin{pmatrix}
B_e & 0 & 0 & 0 & 0 & 0 & 0 \\
-\tau\sigma_h & B_a & 0 & 0 & 0 & 0 & 0 \\
-T\sigma_h & 0 & B_s & 0 & 0 & 0 & 0 \\
0 & 0 & -\rho_{sh} & B_c & 0 & 0 & 0 \\
0 & 0 & 0 & 0 & B_r & 0 & 0 \\
0 & 0 & 0 & 0 & -\sigma_r & \mu_r & 0 \\
0 & 0 & 0 & 0 & 0 & 0 & \mu_v
\end{pmatrix}
$$

$$\mathscr{V}^{-1} = \begin{pmatrix} \frac{1}{B_e} & 0 & 0 & 0 & 0 & 0 & 0 \\ \frac{\tau\sigma_h}{B_eB_a} & \frac{1}{B_a} & 0 & 0 & 0 & 0 & 0 \\ \frac{T\sigma_h}{B_eB_s} & 0 & \frac{1}{B_s} & 0 & 0 & 0 & 0 \\ \frac{\rho_{sh}T\sigma_h}{B_sB_eB_c} & 0 & \frac{\rho_{sh}}{B_sB_c} & \frac{1}{B_c} & 0 & 0 & 0 \\ 0 & 0 & 0 & 0 & \frac{1}{B_r} & 0 & 0 \\ 0 & 0 & 0 & 0 & \frac{\sigma_r}{B_r\mu_r} & \frac{1}{\mu_r} & 0 \\ 0 & 0 & 0 & 0 & 0 & 0 & \frac{1}{\mu_v} \end{pmatrix}$$

$$K_L = \mathscr{F}\mathscr{V}^{-1} = \begin{pmatrix} \frac{\beta_{ah}\tau\sigma_h}{B_eB_a} + T\sigma_h(\frac{\beta_{sh}\sigma_h}{B_eB_s} + \frac{\beta_{ch}\rho_{sh}}{B_sB_eB_c}) & \frac{\beta_{ah}}{B_a} & \frac{\beta_{sh}}{B_s} + \frac{\beta_{ch}\rho_{sh}}{B_sB_c} & \frac{\beta_{ch}}{B_c} & \frac{\Lambda_h\beta_{rh}\Omega_r\sigma_r}{\mu_h(\Omega_r-\mu_r)KB_r\mu_r} & \frac{\Lambda_h\beta_{rh}\Omega_r}{\mu_h(\Omega_r-\mu_r)K\mu_r} & \frac{\Lambda_h\beta_{vh}}{\mu_h\mu_v} \\ 0 & 0 & 0 & 0 & 0 & 0 & 0 \\ 0 & 0 & 0 & 0 & 0 & 0 & 0 \\ 0 & 0 & 0 & 0 & 0 & 0 & 0 \\ 0 & 0 & 0 & 0 & \frac{\beta_r\sigma_r}{B_r\mu_r} & \frac{\beta_r}{\mu_r} & \frac{\beta_{vr}(\Omega_r-\mu_r)K}{\Omega_r\mu_v} \\ 0 & 0 & 0 & 0 & \frac{q\sigma_r}{B_r} & q & 0 \\ \frac{\alpha_{av}\tau\sigma_h}{B_eB_s} + \frac{\alpha_{sv}T\sigma_h}{B_eB_s} + \frac{\alpha_{cv}\rho_{sh}T\sigma_h}{B_eB_sB_c} & \frac{\alpha_{av}}{B_a} & \frac{\alpha_{sv}B_c+\alpha_{cv}\rho_{sh}}{B_cB_s} & \frac{\alpha_{cv}}{B_c} & \frac{\alpha_{rv}\sigma_r}{B_r\mu_r} & \frac{\alpha_{rv}}{\mu_r} & 0 \end{pmatrix}$$

We use an auxiliary E (defined below) to reduce matrix K_L to a lower dimensional matrix K_S which has the same nonzero eigenvalues as K_L[3]. Specifically, we let $E = \begin{pmatrix} 1 & 0 & 0 & 0 & 0 & 0 & 0 \\ 0 & 0 & 0 & 0 & 1 & 0 & 0 \\ 0 & 0 & 0 & 0 & 0 & 1 & 0 \\ 0 & 0 & 0 & 0 & 0 & 0 & 1 \end{pmatrix}$,

and $K_M = E\mathscr{F}\mathscr{V}^{-1}E^T = \begin{pmatrix} \frac{\beta_{ah}\tau\sigma_h}{B_eB_a} + (1-\tau)\sigma_h(\frac{\beta_{sh}\sigma_h}{B_eB_s} + \frac{\beta_{ch}\rho_{sh}}{B_sB_eB_c}) & \frac{\beta_{rh}\Omega_r\Lambda_h\sigma_r}{(\Omega_r-\mu_r)K\mu_hB_r\mu_r} & \frac{\beta_{rh}\Omega_r\Lambda_h}{(\Omega_r-\mu_r)K\mu_h\mu_r} & \frac{\beta_{vh}\Lambda_h}{\mu_h\mu_v} \\ 0 & \frac{\beta_r\sigma_r}{B_r\mu_r} & \frac{\beta_r}{\mu_r} & \frac{\beta_{vr}(\Omega_r-\mu_r)K}{\Omega_r\mu_v} \\ 0 & \frac{q\sigma_r}{B_r} & q & 0 \\ \frac{\alpha_{av}\tau\sigma_h}{B_eB_s} + \frac{\alpha_{sv}T\sigma_h}{B_eB_s} + \frac{\alpha_{cv}\rho_{sh}T\sigma_h}{B_eB_sB_c} & \frac{\alpha_{rv}\sigma_r}{B_r\mu_r} & \frac{\alpha_{rv}}{\mu_r} & 0 \end{pmatrix}$.

Furthermore, a closer inspection reveals that the two middle columns of K_M are collinear, hence all nonzero eigenvalues of K_M coincide with those of the 3×3 matrix

$$K_S = \begin{pmatrix} \frac{\beta_{ah}\tau\sigma_h}{B_eB_a} + (1-\tau)\sigma_h(\frac{\beta_{sh}\sigma_h}{B_eB_s} + \frac{\beta_{ch}\rho_{sh}}{B_sB_eB_c}) & \frac{\beta_{rh}\Omega_r\Lambda_h}{(\Omega_r-\mu_r)K\mu_h\mu_r} & \frac{\beta_{vh}\Lambda_h}{\mu_h\mu_v} \\ 0 & q + \frac{\beta_r\sigma_r}{\mu_rB_r} & \frac{\beta_{vr}(\Omega_r-\mu_r)K\sigma_r}{\Omega_r\mu_vB_r} \\ \frac{\alpha_{av}\tau\sigma_h}{B_eB_s} + \frac{\alpha_{sv}T\sigma_h}{B_eB_s} + \frac{\alpha_{cv}\rho_{sh}T\sigma_h}{B_eB_sB_c} & \frac{\alpha_{rv}}{\mu_r} & 0 \end{pmatrix}.$$

Since K_S is a positive matrix, its largest eigenvalue coincides with the spectral radius. Thus

$$R_0 = \rho(K_L) = \rho(K_M) = \rho(K_S).$$

The following is the system that we referred as Satellite system (in the proof of thm 2.7 in the main paper) where we express S_h, N_h in function of E_h and S_r in function of E_r and $K_0 = \frac{(\Omega_r-\mu_r)K}{\Omega_r}$ is the total rodent at any time.

$$\begin{aligned} \dot{E}_h &= \left(\frac{\beta_aA_h + \beta_{hh}I_h + \beta_zZ_h}{N_h(E_h)} + \frac{\beta_{rh}I_r}{K_0} + \beta_{vh}V\right)S_h(E_h) - (\mu_h + \sigma_h)E_h, \\ \dot{A}_h &= \tau\sigma_hE_h - (\mu_h + \gamma_a)A_h, \\ \dot{I}_h &= (1-\tau)\sigma_hE_h - (\mu_h + \gamma_i + \rho_i + \delta_h)I_h, \\ \dot{Z}_h &= \rho_iI_h - (\mu_h + \gamma_z + \delta_z)Z_h, \\ \dot{E}_r &= \left(\frac{\beta_{rr}I_r}{K_0} + \beta_{vr}V\right)S_r(E_r) - (\sigma_r + \mu_r)E_r, \\ \dot{I}_r &= q\mu_rI_r + \sigma_rE_r - \mu_rI_r, \\ \dot{V} &= \alpha_rI_r + \alpha_aA_h + \alpha_hI_h + \alpha_zZ_h - \mu_vV. \end{aligned} \tag{S1.2}$$

The Jacobian matrix of the satellite system at the origin

$$\hat{J}(0) = \begin{pmatrix} -B_e & \beta_a & \beta_{hh} & \beta_z & 0 & \frac{\Lambda_h \beta_{rh} \Omega_r}{\mu_h(\Omega_r-\mu_r)K} & \frac{\Lambda_h \beta_{vh}}{\mu_h} & \\ \tau\sigma_h & -B_a & 0 & 0 & 0 & 0 & 0 & \\ (1-\tau)\,\sigma_h & 0 & -B_i & 0 & 0 & 0 & 0 & 0 \\ 0 & \rho_i & -B_z & 0 & 0 & 0 & 0 & \\ 0 & 0 & 0 & 0 & -B_r & \beta_r & \frac{\beta_{vr}(\Omega_r-\mu_r)K}{\Omega_r} & \\ 0 & 0 & 0 & 0 & \sigma_r & (-1+q)\,\mu_r & 0 & \\ 0 & \alpha_a & \alpha_h & \alpha_z & 0 & \alpha_r & -\mu_v & \end{pmatrix}$$

is a submatrix of the Jacobian matrix of the full model at the DFE. In particular, it has the same principal eigenvalue ρ which is positive if and only if $R_0 > 1$.

Lemma S1.1. *Consider the following function with $x \in (0,B)$:*

$$F(x) = \frac{Ax}{B-x} - \frac{Cx}{D-x} - Ex \tag{S1.3}$$

Where $A > 0$, $0 < B < D$ and $C,D \geq 0$ then for all $x \in (0,B)$, the part of the graph of $F(x)$ in the first quadrant is a curve that is monotonically increasing and has a positive second derivative (concave up).

Proof. (of lemma S1.1):
We have, $F'(x) = \frac{AB}{(B-x)^2} - \frac{CD}{(D-x)^2} - E$ and $F''(x) = \frac{2AB}{(B-x)^3} - \frac{2CD}{(D-x)^3} = \frac{2AB}{(D-X)^3}\left[\left(\frac{D-x}{B-x}\right)^3 - \frac{CD}{AB}\right]$.
$\forall x \in (0,B)$, $\frac{D}{B} - \frac{D-x}{B-x} = \frac{(B-d)x}{B(B-x)} < 0 \implies 1 < \frac{D}{B} < \frac{D-x}{B-x}$.
Now, assume $x \in (0,B)$ such that $F(x)$ is positive, then $\frac{A}{B-x} > \frac{C}{D-x} + E > \frac{C}{D-x}$
Thus, $\frac{C}{A} < \frac{D-x}{B-x}$, $\frac{C}{A} < \left(\frac{D-x}{B-x}\right)^2$ and $\frac{CD}{AB} < \left(\frac{D-x}{B-x}\right)^3$. Therefore $F''(x) > 0$.
Furthermore, from the assumption that $F(x) > 0$, we have:
$E < \frac{A}{B-x} - \frac{C}{D-x} \implies F'(x) > \left(\frac{AB}{(B-x)^2} - \frac{CD}{(D-x)^2}\right) - \left(\frac{A}{B-x} - \frac{C}{D-x}\right) = \left(\frac{AB}{(B-x)^2} - \frac{A}{B-x}\right) - \left(\frac{CD}{(D-x)^2} - \frac{C}{D-x}\right)$
$= \frac{Ax}{(B-x)^2} - \frac{Cx}{(D-x)^2} = \frac{Ax}{(D-x)^2}\left[\left(\frac{D-x}{B-x}\right)^2 - \frac{C}{A}\right] > 0$ □

Now, at the steady-state, the left hand-side of the system (S1.1) is equal to zero. Expressing all human components through the variable $E_h := x$ and all the rodent components through the variable $E_r := y$, combining the coefficients, we end up with the system of two equations of the form:

$$\begin{aligned} y = \quad & F_1(x) = \frac{A_1 x}{B_1 - x} - \frac{C_1 x}{D-x} - E_1 x, \\ x = \quad & F_2(y) = \frac{A_2 y}{B_2 - y} - E_2 y. \end{aligned} \tag{S1.4}$$

where the parameters in both functions F_1 and F_2 satisfy the assumptions of the preceding section. The coordinates of the positive (endemic) equilibrium must satisfy the condition $(x,y) \in (0,B_1) \times (0,B_2)$, and by the lemma (S1.1) each of the graphs $y = F_1(x)$ and $x = F_2(y)$ is increasing and concave up for $(x,y) \in (0,B_1) \times (0,B_2)$ in their respective coordinate systems. Since $F_1(0) = F_2(0) = 0$ is one point of intersection of these curves, they can intersect at most once inside $(0,B_1) \times (0,B_2)$. Therefore, there exists at most one endemic equilibrium for the full model.

Theorem S1.2. *The Disease Free Equilibrium (DFE) of the full model is asymptotically globally stable whenever the basic reproduction number is less than one.*

Proof. In (S1.1), the dynamics of the total human population is governed by the equation

$$\dot{N}_h = \Lambda_h - \mu_h N_h - \delta_{sh} I_{sh} - \delta_{ch} I_{ch} \leq \Lambda_h - \mu_h N_h,$$

therefore for any nonnegative solution of (S1.1) we have that

$$\limsup_{t \to +\infty} N_h(t) \leq \frac{\Lambda_h}{\mu_h} := N_h^0.$$

Additionally, the dynamics of the total rodent population is governed by the equation

$$\dot{N}_r = \Omega_r(1-\frac{N_r}{K})N_r - \mu_r N_r = \tilde{\Omega}_r(1-\frac{N_r}{\tilde{K}})N_r,$$

where $\tilde{\Omega}_r = \Omega_r - \mu_r$ and $\tilde{K} = \frac{K(\Omega_r-\mu_r)}{\Omega_r}$. We assume that both quantities $\tilde{\Omega}_r$ and $\tilde{K}$ are positive, otherwise, the rodent population becomes extinct in the long run. It readily follows that

$$N_r(0) > 0 \Rightarrow \lim_{t\to+\infty} N_r(t) = \tilde{K}.$$

Consequently, we may further restrict (S1.1) to the forward invariant set $\tilde{\mathbb{D}} = \{N_h(t) \leq N_h^0,\ N_r(t) = \tilde{K}\} \subset \mathbb{R}_+^9$ which attracts all positive solutions of (S1.1) and obtain the following limiting system

$$\begin{aligned}
\dot{S}_h &= \Lambda_h - \left(\frac{\beta_{ah}I_{ah}+\beta_{sh}I_{sh}+\beta_{ch}I_{ch}}{N_h} + \tilde{\beta}_{rh}I_r + \beta_{vh}V\right)S_h - \mu_h S_h,\\
\dot{E}_h &= \left(\frac{\beta_{ah}I_{ah}+\beta_{sh}I_{sh}+\beta_{ch}I_{ch}}{N_h} + \tilde{\beta}_{rh}I_r + \beta_{vh}V\right)S_h - B_e E_h,\\
\dot{I}_{ah} &= \tau\sigma_h E_h - B_a I_{ah},\\
\dot{I}_{sh} &= (1-\tau)\sigma_h E_h - B_s I_{sh},\\
\dot{I}_{ch} &= \rho_{sh}I_{sh} - B_c I_{ch},\\
\dot{R}_h &= \gamma_{ch}I_{ch} + \gamma_{sh}I_{sh} + \gamma_{ah}I_{ah} - \mu_h R_h,\\
\dot{E}_r &= \left(\tilde{\beta}_{rr}I_r + \beta_{vr}V\right)(\tilde{K} - E_r - I_r) - B_r E_r,\\
\dot{I}_r &= \sigma_r E_r - (1-q)\mu_r I_r,\\
\dot{V} &= \alpha_{rv}I_r + \alpha_{av}I_{ah} + \alpha_{sv}I_{sh} + \alpha_{cv}I_{ch} - \mu_v V.
\end{aligned} \tag{S1.5}$$

where

$$\tilde{\beta}_{rh} = \frac{\beta_{rh}}{\tilde{K}},\ \tilde{\beta}_{rr} = \frac{\beta_{rr}}{\tilde{K}}.$$

The DFE in the limiting system (S1.5) is given by

$$S_h = N_h^0, \quad E_h = I_{ah} = I_{sh} = I_{ch} = R_h = E_r = I_r = V = 0.$$

At the DFE, the submatrix of the Jacobian that corresponds to the infectious compartments $X := (E_h, I_{ah}, I_{sh}, I_{ch}, E_r, I_r, V)^T$ is given by

$$\tilde{J} = \begin{bmatrix}
-B_e & \beta_{ah} & \beta_{sh} & \beta_{ch} & 0 & \tilde{\beta}_{rh}N_h^0 & \beta_{vh}N_h^0\\
\tau\sigma_h & -B_a & 0 & 0 & 0 & 0 & 0\\
(1-\tau)\sigma_h & 0 & -B_s & 0 & 0 & 0 & 0\\
0 & 0 & \rho_{sh} & -B_c & 0 & 0 & 0\\
0 & 0 & 0 & 0 & -B_r & \tilde{\beta}_{rr}\tilde{K} & \beta_{vr}\tilde{K}\\
0 & 0 & 0 & 0 & \sigma_r & -(1-q)\mu_r & 0\\
0 & \alpha_{av} & \alpha_{sv} & \alpha_{cv} & 0 & \alpha_{rv} & -\mu_v
\end{bmatrix}.$$

If we assume that $R_0 < 1$, then the DFE is LAS and the principal eigenvalue of $\tilde{J}$ is negative. Since $\tilde{J}$ is irreducible and quasi-positiive, Perron-Frobenius Theorem guarantees the existence of $\lambda > 0$ and $W > 0$ such that $W^T\tilde{J} = -\lambda W^T$. Letting $F(X) = W^T X$, we observe that due to (S1.5),

$$X \geq 0 \Rightarrow \dot{F}(X(t)) = W^T\dot{X}(t) \leq \tilde{J}X(t) = -\lambda W^T X(t) = -\lambda F(X(t)),$$

which readily implies that $F(X(t)) \to 0$ and consequently $X(t) \to 0$ as $t \to +\infty$. A standard limiting argument then shows that $S_h(t) \to N_h^0$, hence the DFE attracts all solutions of (S1.5) in the set $\tilde{\mathbb{D}}$. This proves that the DFE is GAS. □

Theorem S1.3. *The DFE of the full model (S1.1) is globally asymptotically stable whenever $\mathscr{R}_0 < 1$.*

Proof. From the method section in the main paper, for any non-negative solutions of the model (S1.1), we have that $\limsup_{t\to+\infty} N_h(t) \leq \dfrac{\Lambda_h}{\mu_h} := N_h^0$. Additionally, the dynamics of the total rodent population is governed by the equation $\dot{N}_r =$

$\tilde{\Omega}_r(1-\frac{N_r}{\tilde{K}})N_r$, where $\tilde{\Omega}_r$ and $\tilde{K}$ are defined in Eqs. (2.2) of the main paper. We assume that the quantities $\tilde{\Omega}_r$ and $\tilde{K}$ are positive, otherwise, the rodent population becomes extinct in the long run. It follows that $N_r(0)>0 \Rightarrow \lim_{t\to+\infty} N_r(t)=\tilde{K}$. Consequently, we can further restrict the model (S1.1) to the forward invariant set $\tilde{\mathbb{D}} := \{N_h(t)\le N_h^0,\ N_r(t)=\tilde{K}\} \subset \mathbb{R}_+^9$ which attracts all positive solutions of the model (S1.1) and obtain a limiting system by setting $\tilde{\beta}_{rh}=\frac{\beta_{rh}}{\tilde{K}}$ and $\tilde{\beta}_{rr}=\frac{\beta_{rr}}{\tilde{K}}$ in Eqs. (S1.1) (see Eqs.(S1.5) in the SI).

The DFE of the limiting system (Eqs. (S1.5) in the SI) is given by $S_h=N_h^0, \quad E_h=I_{ah}=I_{sh}=I_{ch}=R_h=E_r=I_r=V=0$. At the DFE, the submatrix of the Jacobian that corresponds to the infectious compartments $X:=(E_h,I_{ah},I_{sh},I_{ch},E_r,I_r,V)^T$ is given by

$$\tilde{J}=\begin{pmatrix} -B_e & \beta_{ah} & \beta_{sh} & \beta_{ch} & 0 & \tilde{\beta}_{rh}N_h^0 & \beta_{vh}N_h^0 \\ \tau\sigma_h & -B_a & 0 & 0 & 0 & 0 & 0 \\ (1-\tau)\,\sigma_h & 0 & -B_s & 0 & 0 & 0 & 0 \\ 0 & 0 & \rho_{sh} & -B_c & 0 & 0 & 0 \\ 0 & 0 & 0 & 0 & -B_r & \tilde{\beta}_{rr}\tilde{K} & \beta_{vr}\tilde{K} \\ 0 & 0 & 0 & 0 & \sigma_r & -(1-q)\mu_r & 0 \\ 0 & \alpha_{av} & \alpha_{sv} & \alpha_{cv} & 0 & \alpha_{rv} & -\mu_v \end{pmatrix}.$$

If we assume that $\mathscr{R}_0<1$, then the DFE is LAS and the principal eigenvalue of $\tilde{J}$ is negative. Since $\tilde{J}$ is irreducible and quasi-positive, Perron-Frobenius Theorem guarantees the existence of $\lambda>0$ and $W>0$ such that $W^T\tilde{J}=-\lambda W^T$. Letting $F(X)=W^TX$, we observe that due to Eqs. (S1.5) in the SI,

$$X\ge 0 \Rightarrow \dot{F}(X(t))=W^T\dot{X}(t)\le W^T\tilde{J}X(t)=-\lambda W^TX(t)=-\lambda F(X(t)),$$

which readily implies that $F(X(t))\to 0$ and consequently $X(t)\to 0$ as $t\to+\infty$. A standard limiting argument then shows that $S_h(t)\to N_h^0$, hence the DFE attracts all solutions of Eqs. (S1.5) in the set $\tilde{\mathbb{D}}$. This proves that the DFE is GAS. □

Theorem S1.4. *If $\mathscr{R}_0>1$, the full model (S1.1) admits a unique endemic equilibrium.*

Proof. We prove the existence and uniqueness in two separate steps.

(i) Let $X=(E_h,I_{ah},I_{sh},I_{ch},E_r,I_r)^T$ and $\dot{X}=f(X)$ be a sub-system (satellite system) of the model (S1.1) consisting of equations for infected and infectious classes. It should be mentioned that S_h,N_h,S_r, and N_r in this subsystem have been replaced by $S_h(E_h)=\frac{\Lambda_h}{\mu_h}-Q_2E_h$, $N_h(E_h)=\frac{\Lambda_h}{\mu_h}-Q_1E_h$, $S_r(E_r)=\tilde{K}-Q_3E_r$, and $N_r(E_r)=\tilde{K}$ with $Q_3=\frac{(1-q)\mu_r+\sigma_r}{(1-q)\mu_r}$, obtained by expressing S_h and N_h as functions of E_h and S_r as a function of E_r. The following properties of the satellite system are immediate from its construction: the trivial equilibrium of the satellite system corresponds to the DFE of the model (S1.1) and any positive equilibrium of the satellite system corresponds to an endemic equilibrium of (S1.1). Also, the Jacobian matrix of the satellite system evaluated at the origin (Section 1.1 of the SI) is a submatrix of the Jacobian matrix of the full model at the DFE. In particular, it has the same principal eigenvalue (ρ), which is positive if and only if $\mathscr{R}_0>1$. Additionally, the satellite system is cooperative in the positive orthant (i.e., all off-diagonal entries of $\hat{J}$ are nonnegative). Furthermore, any positive solution of the satellite system starting near the origin, remains bounded. Indeed, $\limsup_{t\to+\infty}E_h(t)<\frac{\Lambda_h}{\mu_hQ_2}$ and $\limsup_{t\to+\infty}E_r(t)<\frac{\tilde{K}}{Q_3}$, which implies that the remaining state variables are bounded as well (since they can be expressed as functions of E_h or E_r). Suppose that $\mathscr{R}_0>1$. Then the principal eigenvalue ρ is positive and since $\hat{J}(0)$ is irreducible, the associated eigenvector w is strictly positive. Hence, any solution $X(t)$ of the satellite system with $X(0)=bv>0$ with $0<b\ll 1$ will have a positive initial velocity $\dot{X}(0)\sim\rho bv+o(b)>0$. Since the satellite system is cooperative, it follows that $\dot{X}>0$ for all $t\ge 0$, which means that all components of $X(t)$ are monotonically increasing. Combining this property with the boundedness of $X(t)$, we conclude that any such solution $x(t)$ must converge to a positive equilibrium of the satellite system. Since the positive equilibria of the satellite system coincide with the endemic equilibria of the model (S1.1), there exists at least one endemic equilibrium whenever $\mathscr{R}_0>1$.

(ii) After proving that the full model (S1.1) admits at least one endemic equilibrium in part (i) of the Theorem, we now show that the full model (S1.1) admits at most one endemic equilibrium. The proof follows directly from Lemma S1.1 (and its consequence under the same proof) , by setting:
$A_1=\frac{B_eK_0\mu_r\mu_v(1-q)}{(\beta_{vh}\tilde{K}\alpha_{rv}\sigma_r+\mu_v\beta_{rh}\sigma_r)Q_2}$, $B_1=\frac{\Lambda_h}{\mu_hQ_2}$, $C_1=\frac{\tilde{K}\mu_r\mu_v(1-q)\sigma_h[\tau B_sB_c+(1-\tau)B_a(B_c+\rho_{sh})]}{(\beta_{vh}\tilde{K}\alpha_{rv}\sigma_r+\mu_v\beta_{rh}\sigma_r)B_aB_cB_sQ_2}$, $D_1=\frac{\Lambda_h}{\mu_hQ_1}$,
$E_1=\frac{\beta_{vh}\tilde{K}\mu_r(1-q)\sigma_h[\tau B_cB_s\alpha_{av}+(1-\tau)B_a(B_c\alpha_{sv}+\alpha_{cv}\rho_{sh})]}{B_aB_cB_s(\beta_{vh}\tilde{K}\alpha_{rv}\sigma_r+\mu_v\beta_{rh}\sigma_r)}$ in F_1 and $A_2=\frac{B_rB_aB_cB_s}{\beta_{vr}Q_3\sigma_h[\tau B_cB_s\alpha_{av}+(1-\tau)B_a(B_c\alpha_{sv}+\alpha_{cv}\rho_{sh})]}$, $B_2=\frac{\tilde{K}}{Q_3}$, $C_2=D_2=0$, and $E_2=\frac{B_aB_cB_s(\beta_{rr}\sigma_r\mu_v+\tilde{K}\beta_{vr}\alpha_{rv}\sigma_r)}{\beta_{vr}\tilde{K}\mu_r\mu_v(1-q)\sigma_h(\tau B_cB_s+(1-\tau)(B_aB_c+\rho_{sh}B_a)}$ in F_2.

$\square$

Theorem S1.5. *If $\mathscr{R}_0 > 1$, the disease is uniformly strongly persistent.*

Proof. For any non-negative solution of (S1.1), there exists $T > 0$ such that $N_h(t) < M, \quad N_r(t) \le M, \quad M := \max(N_h^0, \tilde{K}) + 1$, for all $t \ge T$. Consequently, $\dot{V} < (\alpha_{rv} + \alpha_{av} + \alpha_{sv} + \alpha_{cv})M - \mu_v V$, which implies that $\exists T_1 > T : \; \forall t > T_1, \quad V(t) < V^0 := \frac{(\alpha_{rv}+\alpha_{av}+\alpha_{sv}+\alpha_{cv})M}{\mu_v}$. As a result, we have that $\dot{S}_h \ge \Lambda_h - \left(\beta_{ah} + \beta_{sh} + \beta_{ch} + \beta_{rh} + \beta_{vh}V^0 + \mu_h\right) S_h$. Hence, $\liminf_{t\to\infty} S_h(t) \ge S_h^0 := \frac{\Lambda_h}{\beta_{ah}+\beta_{sh}+\beta_{ch}+\beta_{rh}+\beta_{vh}V^0+\mu_h}$. Similarly, we have that $\dot{S}_r \ge \mu_r(1-q)\tilde{K} - (\beta_{rr} + \beta_{vr}V^0 + \mu_r)S_r$, hence $\liminf_{t\to\infty} S_r(t) \ge S_r^0 := \frac{\mu_r(1-q)\tilde{K}}{\beta_{rr}+\beta_{vr}V^0+\mu_r}$.

For the remainder of this section, we simply assume that $S_h(t), S_r(t) \in [m, M]$ for all $t \ge 0$. Here, $m := \min(S_h^0, S_r^0)$. Substituting these bounds into (S1.1), we obtain the following inequalities:

$$\begin{aligned}
\dot{E}_h &\ge \left(\tilde{\beta}_{rh} I_r + \beta_{vh} V\right) m - B_e E_h, \quad \dot{I}_{ah} \ge \tau\sigma_h E_h - B_a I_{ah}, \quad \dot{E}_r \ge \beta_{vr} m V - B_r E_r, \\
\dot{I}_r &\ge \sigma_r E_r - (1-q)\mu_r I_r, \quad \dot{V} \ge \alpha_{rv} I_r + \alpha_{av} I_{ah} - \mu_v V.
\end{aligned} \tag{S1.6}$$

Suppose that $E_h(t)$ is uniformly weakly persistent (UWP), that is, there exist $\varepsilon > 0$ and a sequence $t_n \uparrow \infty$ such that $E_h(t_n) \ge \varepsilon$. Since $\dot{E}_h \ge -B_e E_h$, $E_h(\tau) \ge \varepsilon e^{-B_e}$ for all $s \in [t_n, t_n + 1]$, which implies that

$$I_{ah}(t_n + 1) \ge \varepsilon\tau\sigma_h \exp(-(B_e + B_a)),$$

therefore, $I_{ah}(t)$ is UWP. Using other inequalities in (S1.6), we conclude that the remaining state variables are all UWP.

Now suppose that $E_h(t)$ is uniformly strongly persistent (USP), that is, there exist $\delta > 0$ and a $T > 0$ such that $E_h(t) \ge \delta$ for all $t > T$. Then the inequality

$$\dot{I}_{ah} \ge \tau\sigma_h\delta - B_a I_{ah}, \; t > T,$$

implies that $I_{ah}(t)$ is USP. Using other inequalities in (S1.6), we conclude that the remaining state variables are all USP.

It is easy to see from (S1.6) that we have the cyclic graph $E_h \to I_{ah} \to V \to E_r \to I_r \to E_h$ where weak/strong persistence of any given node is passed to all downstream nodes. Therefore, we conclude that all infected classes (the humans, the rodents, and the environment) persist together or become extinct together, and proving a weak/strong persistence of any one of them is sufficient to prove similar persistence for all of them. If we assume that $\mathscr{R}_0 > 1$, then the DFE is unstable and the principal eigenvalue of $\tilde{J}$ is positive. Since $\tilde{J}$ is irreducible and quasi-positive, Perron-Frobenius Theorem guarantees the existence of $\lambda > 0$ and $W > 0$ such that $W^T\tilde{J} = \lambda W^T$. Now we consider a positive solution of Eqs. (S1.5) in SI and let $F(X) = W^T X$. Furthermore, for sake of contradiction we suppose that $F(X(t)) \to 0$ as $t \to \infty$. Then our solution must converge to the DFE, consequently, there exist a sufficiently small $\eta \in (0, \lambda)$ and $T \ge 0$ such that

$$\dot{F}(X(t)) = W^T\dot{X}(t) \ge \tilde{J}X(t) - \eta F(X(t)) = \lambda W^T X(t) - \eta F(X(t)) = (\lambda - \eta)F(X(t)),$$

for all $t > T$, which implies that $F(X(t)) \to +\infty$ as $t \to +\infty$ in contradiction to our assumption. This proves that our solution is uniformly weakly F-persistent. By Theorem 4.5 in[1], this UWP implies that all positive solutions are uniformly strongly F-persistent. To check that the assumptions of Theorem 4.5 in[1] are satisfied, consider the set $\tilde{\mathbb{D}}$ which attracts all non-negative solutions of the system (S1.1). Then, the third assumption in[1] holds since $\tilde{\mathbb{D}}$ attracts non-negative solution of (S1.1) if for $y \in Y = \mathbb{R}_+^{10}$ we have $d(\Phi(t,y), \tilde{\mathbb{D}}) \to 0$ at $t \to \infty$. Now, using $Y = \mathbb{R}_+^{10}$, $J = \mathbb{R}_+$, $\rho(y(t)) = F(X(t)) = W^T X(t)$, $y(t) = (S_h(t), X(t), S_r(t), V(t))$ and $\sigma(t,y) = \rho(\Phi(t,y))$ and $\Phi : J \times Y \to Y$ (i.e:,$\Phi(t, y(0)) = y(t)$) is the semiflow defined with state space Y and time-set J. With this setting, the first assumption (continuity of $\rho \circ \Phi$) is verified because ρ and Φ are both continuous. Finally, the second assumption (if $s, t \in J$ there is no $y \in \tilde{\mathbb{D}}$ that satisfies $\rho(y) > 0, \sigma(s,y) = 0$ & $\sigma(s+t, y) > 0$) holds because the set with $\rho(y) = 0$ is invariant. Combining the F-persistence with the previous remarks, we conclude that $\mathscr{R}_0 > 1$ implies the uniform strong persistence of all of the infectious variables. $\square$

S2 The rodent-free model

The rodent-free model is obtained by setting all rodent related variables in the model (S1.1) to zero. That is, $S_r = E_r = I_r = 0$. The equations of the model are given by:

$$
\begin{aligned}
\dot{S_h} &= \Lambda_h - \left(\frac{\beta_{ah}I_{ah} + \beta_{sh}I_{sh} + \beta_{ch}I_{ch}}{N_h} + \beta_{vh}V\right) S_h - \mu_h S_h \\
\dot{E_h} &= \left(\frac{\beta_{ah}I_{ah} + \beta_{sh}I_{sh} + \beta_{ch}I_{ch}}{N_h} + \beta_{vh}V\right) S_h - (\mu_h + \sigma_h)E_h, \\
\dot{I_{ah}} &= \tau\,\sigma_h E_h - (\mu_h + \gamma_{ah})\,I_{ah}, \\
\dot{I_{sh}} &= (1-\tau)\,\sigma_h E_h - (\mu_h + \gamma_{sh} + \rho_{sh} + \delta_{sh})I_{sh}, \\
\dot{I_{ch}} &= \rho_{sh}I_{sh} - (\mu_h + \gamma_{ch} + \delta_{ch})I_{ch}, \\
\dot{R_h} &= \gamma_{ch}\,I_{ch} + \gamma_{sh}I_{sh} + \gamma_{ah}I_{ah} - \mu_h R_h, \\
\dot{V} &= \alpha_{av}I_{ah} + \alpha_{sv}I_{sh} + \alpha_{cv}I_{ch} - \mu_v V.
\end{aligned}
\tag{S1.7}
$$

We use the next generation operator approach[2,3] to compute the basic reproduction number of the model (S1.7). Specifically, the matrices of new infections ($\mathscr{F}$) and transitions ($\mathscr{V}$) are given by

$$
\mathscr{F} = \begin{pmatrix}
0 & \beta_{ah} & \beta_{sh} & \beta_{ch} & \dfrac{\beta_{vh}\Lambda_h}{\mu_h} \\
0 & 0 & 0 & 0 & 0 \\
0 & 0 & 0 & 0 & 0 \\
0 & 0 & 0 & 0 & 0 \\
0 & \alpha_{av} & \alpha_{sv} & \alpha_{cv} & 0
\end{pmatrix}
\quad \text{and} \quad
\mathscr{V} = \begin{pmatrix}
B_e & 0 & 0 & 0 & 0 \\
-\tau\sigma_h & B_a & 0 & 0 & 0 \\
-(1-\tau)\sigma_h & 0 & B_s & 0 & 0 \\
0 & 0 & -\rho_{sh} & B_c & 0 \\
0 & 0 & 0 & 0 & \mu_v
\end{pmatrix},
$$

while the next generation matrix $\mathscr{F}\mathscr{V}^{-1}$ is given by

$$
\mathscr{F}\mathscr{V}^{-1} = \begin{pmatrix}
\dfrac{\beta_{ah}\tau\sigma_h}{B_eB_a} + \dfrac{\beta_{sh}\sigma_h(1-\tau)}{B_sB_e} + \dfrac{\beta_{ch}\rho_{sh}\sigma_h(1-\tau)}{B_cB_sB_e} & \dfrac{\beta_{ah}}{B_a} & \dfrac{\beta_{sh}}{B_s} + \dfrac{\beta_{ch}\rho_{sh}}{B_cB_s} & \dfrac{\beta_{ch}}{B_c} & \dfrac{\beta_{vh}\Lambda_h}{\mu_h\mu_v} \\
0 & 0 & 0 & 0 & 0 \\
0 & 0 & 0 & 0 & 0 \\
0 & 0 & 0 & 0 & 0 \\
\dfrac{\alpha_{av}\tau\sigma_h}{B_eB_a} + \dfrac{\alpha_{sv}\sigma_h(1-\tau)}{B_sB_e} + \dfrac{\alpha_{cv}\rho_{sh}\sigma_h(1-\tau)}{B_cB_sB_e} & \dfrac{\alpha_{av}}{B_a} & \dfrac{\alpha_{sv}B_c + \alpha_{cv}\rho_{sh}}{B_cB_s} & \dfrac{\alpha_{cv}}{B_c} & 0
\end{pmatrix}
$$

Lemma S1.6. $R_{0h} + R_{0v} \le 1 \Leftrightarrow R_{0hv} \le 1$.

Proof. The quantity R_{0hv} satisfies

$$
R_{0hv}^2 = R_{0h}R_{0hv} + R_{0v} \Leftrightarrow 1 = \frac{R_{0h}}{R_{0hv}} + \frac{R_{0v}}{R_{0hv}^2}.
$$

Since the function $G(x) = \frac{R_{0h}}{x} + \frac{R_{0v}}{x^2}$ is decreasing in x, it follows that

$$
\operatorname{sign}(R_{0hv} - 1) = \operatorname{sign}(G(1) - 1) = \operatorname{sign}(R_{0h} + R_{0v} - 1).
$$

□

Proof.

$$
\begin{aligned}
2R_{0h} + R_{0v} \le 1 \quad &\Leftrightarrow \quad R_{0v} \le 1 - 2R_{0h} \\
&\Leftrightarrow \quad R_{0h}^2 + R_{0v} \le R_{0h}^2 + 1 - 2R_{0h} \\
&\Leftrightarrow \quad R_{0h}^2 + R_{0v} \le (1 - R_{0h})^2 \\
&\Leftrightarrow \quad \sqrt{R_{0h}^2 + R_{0v}} \le 1 - R_{0h} \text{ or } \sqrt{R_{0h}^2 + R_{0v}} \le R_{0h} - 1 \\
&\Leftrightarrow \quad R_{0h} + \sqrt{R_{0h}^2 + R_{0v}} \le 1 \text{ or } \sqrt{R_{0h}^2 + R_{0v}} - R_{0h} \le -1 \\
&\Leftrightarrow \quad R_{0hv} \le 1 \text{ or } \sqrt{R_{0h}^2 + R_{0v}} - R_{0h} \le -1.
\end{aligned}
$$

The second inequality ($\sqrt{R_{0h}^2 + R_{0v}} - R_{0h} \leq -1$), is not possible since the left hand side is positive but the right hand side is negative. Hence, $2R_{0h} + R_{0v} \leq 1 \Leftrightarrow R_{0hv} \leq 1$. □

The next two results establish the global stability of the disease-free equilibrium of the rodent-free model.

Lemma S1.7. $\text{sign}(R_{0hv} - 1) = \text{sign}(\mathscr{R}_{0h} + \mathscr{R}_{0v} - 1)$.

Proof. Since, $\mathscr{R}_{0hv} = \frac{\mathscr{R}_{0h} + \sqrt{\mathscr{R}_{0h}^2 + 4\mathscr{R}_{0v}}}{2}$, $\mathscr{R}_{0hv}^2 = \frac{\mathscr{R}_{0h}^2 + 2\mathscr{R}_{0h}\sqrt{\mathscr{R}_{0h}^2 + 4\mathscr{R}_{0v}} + \mathscr{R}_{0h}^2 + 4\mathscr{R}_{0v}}{4} = \frac{2\mathscr{R}_{0h}(\mathscr{R}_{0h} + \sqrt{\mathscr{R}_{0h}^2 + 4\mathscr{R}_{0v}}) + 4\mathscr{R}_{0v}}{4}$. Hence, the quantity R_{0hv} satisfies

$$\mathscr{R}_{0hv}^2 = \mathscr{R}_{0h}\mathscr{R}_{0hv} + \mathscr{R}_{0v} \Leftrightarrow 1 = \frac{\mathscr{R}_{0h}}{\mathscr{R}_{0hv}} + \frac{\mathscr{R}_{0v}}{\mathscr{R}_{0hv}^2}.$$

Since the function $G(\mathscr{R}_{0hv}) = \frac{\mathscr{R}_{0h}}{\mathscr{R}_{0hv}} + \frac{\mathscr{R}_{0v}}{\mathscr{R}_{0hv}^2}$ is decreasing in $\mathscr{R}_{0hv}$, it follows that

$$\text{sign}(\mathscr{R}_{0hv} - 1) = \text{sign}(G(1) - 1) = \text{sign}(\mathscr{R}_{0h} + \mathscr{R}_{0v} - 1).$$

□

Remark S1.8. *Since* $\text{sign}(\mathscr{R}_{0hv} - 1) = \text{sign}(\mathscr{R}_{0h} + \mathscr{R}_{0v} - 1)$ *from Lemma S1.7,* $\mathscr{R}_{0hv} < 1$*, if* $\mathscr{R}_{0h} + \mathscr{R}_{0v} < 1$.

Theorem S1.9. *The disease-free equilibrium (DFE_{hv}), of the rodent-free model given by Eqs. (S2.1) is globally asymptotically stable whenever* $\mathscr{R}_{0hv} < 1$ *and unstable when* $\mathscr{R}_{0hv} > 1$.

Proof. Consider the Lyapunov function $\mathscr{L} = E_h + w_1 I_{ah} + w_2 I_{sh} + w_3 I_{ch} + w_4 V$, where $w_1 = \frac{\beta_{ah}\mu_h\mu_v + \beta_{vh}\Lambda_{sv}\alpha_{av}}{B_a\mu_h\mu_v}$, $w_2 = \frac{\beta_s}{B_s} + \frac{\rho_{sh}\beta_{ch}}{B_s B_c} + \frac{\beta_{vh}\Lambda_h\alpha_{cv}\rho_{sh}}{B_s B_c\mu_h\mu_v} + \frac{\beta_{vh}\Lambda_h\alpha_{sv}\rho_{sh}}{B_s\mu_h\mu_v}$ $w_3 = \frac{\beta_{cv}}{B_c} + \frac{\beta_{vh}\Lambda_h\alpha_{cv}}{B_c\mu_h\mu_v}$. Clearly, $\mathscr{L} > 0$ as long as the infectious variables are not all zero.

$$\begin{aligned}
\dot{\mathscr{L}} &= w_1\dot{E}_h + w_2\dot{I_{ah}} + w_3\dot{I_{sh}} + w_4\dot{I_{ch}} + w_6\dot{V} \\
&\leq (\beta_{ah}I_{ah} + \beta_{sh}I_{sh} + \beta_{ch}I_{ch} + \frac{\beta_{vh}V\Lambda_h}{\mu_h} - B_e E_h) + w_1(\tau\sigma_h E_h - B_a I_{ah}) + w_2((1-\tau)\sigma_h E_h - B_s I_{sh}) \\
&\quad + w_3(\rho_{sh}I_{sh} - B_c I_{ch}) + w_4(\alpha_{av}I_{ah} + \alpha_{sv}I_{sh} + \alpha_{cv}I_{ch} - \mu_v V), \text{ since } 0 \leq \frac{S_h}{N_h} \leq 1, \text{ and } 0 \leq S_h \leq \frac{\Lambda_h}{\mu_h}. \\
&= B_e(\mathscr{R}_{0h} + \mathscr{R}_{0v} - 1)E_h.
\end{aligned}$$

By Lemma Lemma 3.3 in the main paper, $\mathscr{R}_{0h} + \mathscr{R}_{0v} \leq 1 \Leftrightarrow \mathscr{R}_{0hv} \leq 1$. Hence, $\dot{\mathscr{L}} \leq B_e(\mathscr{R}_{0h} + \mathscr{R}_{0v} - 1)E_h \leq 0 \Leftrightarrow \mathscr{R}_{0hv} < 1$. Moreover, the largest compact invariant set in $\{(S_h, E_h, I_{ah}, I_{sh}, I_{ch}, R_h, V) \in \mathbb{D} : \dot{\mathscr{L}} = 0\}$ is the singleton $\left\{\left(\frac{\Lambda_h}{\mu_h}, 0, 0, 0, 0, 0, 0\right)\right\}$. According to LaSalle's Invariance Principle, every solution of (S2.1), with initial conditions in $\mathbb{D}$ converges to $\left\{\left(\frac{\Lambda_h}{\mu_h}, 0, 0, 0, 0, 0, 0\right)\right\}$ as $t \to \infty$. Hence, the disease-free equilibrium solution (DFE_{hv}) of the rodent-free model (S2.1) is globally asymptotically stable in $\mathbb{D}$ when $\mathscr{R}_{0hv} < 1$. □

Theorem S1.10. *The rodent-free sub-system (S2.1) admits a single endemic equilibrium whenever* $\mathscr{R}_{0hv} > 1$.

Proof. We first prove that the rodent-free sub-system (S2.1), has at least one endemic equilibrium. To do this, it is convenient to express N_h and S_h as functions of E_h (i.e., $N_h(E_h) = \frac{\Lambda_h}{\mu_h} - Q_1 E_h$ and $S_h(E_h) = \frac{\Lambda_h}{\mu_h} - Q_2 E_h$, where $Q_1 = (1-\tau)\left(\frac{\delta_{sh}\sigma_h}{B_s\mu_h} + \frac{\delta_{ch}\rho_{sh}\sigma_h}{B_s B_c\mu_h}\right)$ and $Q_2 = Q_1 + 1 + \frac{\tau\sigma_h}{B_a} + \frac{T\sigma_h}{B_s} + \frac{\rho_{sh}T\sigma_h}{B_s B_c} + \frac{\gamma_{ah}\tau\sigma_h}{B_a\mu_h} + \frac{\gamma_{sh}T\sigma_h}{B_s\mu_h} + \frac{\gamma_{ch}\rho_{sh}T\sigma_h}{B_s B_c\mu_h}$ in the resulting system referred to as a "Satellite" system ((S1.2) in SI). The following properties of the satellite system are immediate from its construction. First, the trivial equilibrium of the satellite system corresponds to the disease-free equilibrium (DFE_{hv}) of the rodent-free model (S2.1). Second, any positive equilibrium of the satellite system corresponds to an endemic equilibrium of the rodent-free model. Third, the Jacobian matrix of the satellite system at the origin

$$\hat{J}(0) = \begin{pmatrix}
-B_e & \beta_a & \beta_{sh} & \beta_{ch} & \frac{\Lambda_h\beta_{vh}}{\mu_h} \\
\tau\sigma_h & -B_a & 0 & 0 & 0 \\
(1-\tau)\sigma_h & 0 & -B_s & 0 & 0 \\
0 & 0 & \rho_{sh} & -B_c & 0 \\
0 & \alpha_{av} & \alpha_{sv} & \alpha_{cv} & -\mu_v
\end{pmatrix}$$

is a submatrix of the Jacobian matrix of the rodent-free model at the DFE_{hv}. In particular, it has the same principal eigenvalue ρ which is positive if and only if $\mathscr{R}_{0hv} > 1$. Fourth, the satellite system is cooperative in the positive orthant (i.e., off-diagonal entries of $\hat{J}$ are all nonnegative). Lastly, any positive solution of the satellite system starting near the origin, remains bounded. Indeed, we have that $\limsup_{t\to+\infty} E_h(t) < \dfrac{\Lambda_h}{\mu_h Q_2}$, which implies that all of the remaining state variables are bounded as well. Suppose that $R_{0hv} > 1$. Then the principal eigenvalue ρ is positive and since $\hat{J}(0)$ is irreducible, the associated eigenvector w is strictly positive. Hence, any solution $X(t) = (E_h(t), I_{ah}(t), I_{sh}(t), I_{ch}(t), V(t))^T$ of the satellite system with $X(0) = bv > 0$, where $0 < b \ll 1$ will have a positive initial velocity $\dot{X}(0) \sim \rho bv + o(b) > 0$. Since the satellite system is cooperative, it follows that $\dot{X} > 0$ for all $t \geq 0$, which means that all components of $X(t)$ are monotonically increasing. Combining this property with the boundedness of $X(t)$, we conclude that any such solution $X(t)$ must converge to a positive equilibrium of the satellite system. Since the positive equilibria of the satellite system coincide with the endemic equilibria, we find that there exists at least one endemic equilibrium whenever $\mathscr{R}_{0hv} > 1$.

Next, we prove that if $\mathscr{R}_{0hv} > 1$, there exists a unique endemic equilibrium of the rodent-free sub-system (S2.1). Let $E^*_{hv} = (S^*_h, E^*_h, I^*_{ah}, I^*_{sh}, I^*_{ch}, R^*_h, V^*)$ be an endemic equilibrium of Model (S2.1) obtained by setting the right hand-sides of the model to zero, and solving the first, third, fourth, and fifth equations for the other states in terms of E^*_h. Then,

$$
\begin{aligned}
S^*_h &= \frac{\Lambda_h}{\mu_h + \lambda^*_h}, \quad I^*_{ah} = \frac{\tau\sigma_h}{B_a}E^*_h, \quad I^*_{sh} = \frac{(1-\tau)\sigma_h}{B_s}E^*_h, \quad I^*_{ch} = \frac{\rho_{sh}(1-\tau)\sigma_h}{B_sB_c}E^*_h,\\
R^*_h &= \frac{\sigma_h[\tau\gamma_{ah}B_sB_c + (1-\tau)B_a(\gamma_{ch}\rho_{sh} + \gamma_{sh}B_c)]}{\mu_h B_a B_s B_c}E^*_h, \quad N_h = \frac{\Lambda_h}{\mu_h} - \frac{(1-\tau)\sigma_h(\delta_s B_c + \delta_{ch}\rho_{sh})}{B_s B_c \mu_h}E^*_h,\\
V^* &= \frac{\sigma_h[\tau\alpha_{ah}B_sB_c + (1-\tau)B_a(\alpha_{ch}\rho_{sh} + \alpha_{sh}B_c)]}{\mu_v B_a B_s B_c}E^*_h, \quad \lambda^*_h = \frac{\beta_{ah}I^*_{ah} + \beta_{sh}I^*_{sh} + \beta_{ch}I^*_{ch}}{N^*_h} + \beta_{vh}V^*.
\end{aligned}
$$

Substituting these equilibria in the second equation of the sub-system and simplifying leads to the equation

$$E^{*2}_h = A_1 E^*_h + A_0, \tag{S1.8}$$

where $A_1 = \frac{\Lambda_h}{\mathscr{R}_{0v}}\left[\left(\mathscr{R}_{0v} + \frac{B_sB_c(\mathscr{R}_{0h}+\mathscr{R}_{0v})}{(1-\tau)\sigma_h(\delta_sB_c+\delta_{ch}\rho_{sh})}\right) - 1\right]$, and $A_0 = \frac{B_sB_c\Lambda_h^2[R_{0h}+R_{0v}-1]}{R_{0v}(1-\tau)\sigma_h(\delta_sB_c+\delta_{ch}\rho_{sh})}$. By lemma (S1.7), sign$(\mathscr{R}_{0hv} - 1) =$ sign$(\mathscr{R}_{0h} + \mathscr{R}_{0v} - 1)$. Therefore, $\mathscr{R}_0 > 1$ if $\mathscr{R}_{0h} + \mathscr{R}_{0v} - 1$. Hence, $A_0 > 0$ if $\mathscr{R}_0 > 1$. If $F(E^*_h) = E^{*2}_h$ and $G(E^*_h) = A_1E^*_h + A_0$, then the straight line (G) and the parabola (F) intersect exactly once in the first quadrant when $A_0 > 0$ (i.e., when $R_{0hv} > 1$), since both F and G are monotonically increasing in the first quadrant. Therefore, the rodent-free sub-system (S2.1) admits a unique a endemic equilibrium when $\mathscr{R}_{0hv} > 1$.

□

S3 The Rodent-virus (human free) model

For this model all human variables were clean out from the full model and we have:

$$
\begin{aligned}
\dot{S}_r &= \Omega_r(1 - \frac{N_r}{K})\,[S_r + E_r + (1-q)I_r] - \left(\frac{\beta_{rr}I_r}{N_r} + \beta_{vr}V\right)S_r - \mu_r S_r,\\
\dot{E}_r &= \left(\frac{\beta_{rr}I_r}{N_r} + \beta_{vr}V\right)S_r - (\sigma_r + \mu_r)E_r,\\
\dot{I}_r &= q\Omega_r(1 - \frac{N_r}{K})I_r + \sigma_r E_r - \mu_r I_r,\\
\dot{V} &= \alpha_{rv}I_r - \mu_v V.
\end{aligned}
\tag{S1.9}
$$

S3.1 Disease-free equilibrium and the basic reproduction number

At a state where there is no disease in rodent population (the Disease Free equilibrium), the solution to the system (S1.9) is denoted by DFE_{rv} is: $DFE_{rv} = (S_{r0}, E_{r0}, I_{r0}, V_0) = (\frac{K(\Omega_r - \mu_r)}{\Omega_r}, 0, 0, 0)$. The basic reproduction number is calculated at DFE using the next generation matrix approach[4]. Here, we let F be the matrix of new infections (at

DFE) and let V be the transition matrix. Then from system (S1.9) we obtain :

$$F = \begin{bmatrix} 0 & \beta_r & \frac{\beta_{vr}(\Omega_r-\mu_r)K}{\Omega_r} \\ 0 & q\mu_r & 0 \\ 0 & \alpha_{rv} & 0 \end{bmatrix}, \quad V = \begin{bmatrix} B_r & 0 & 0 \\ -\sigma_r & \mu_r & 0 \\ 0 & 0 & \mu_v \end{bmatrix}, \quad F \times V^{-1} = \begin{bmatrix} \frac{\beta_r\sigma_r}{B_r\mu_r} & \frac{\beta_r}{\mu_r} & \frac{\beta_{vr}(\Omega_r-\mu_r)K}{\Omega_r\mu_v} \\ \frac{q\sigma_r}{B_r} & q & 0 \\ \frac{\alpha_{rv}\sigma_r}{B_r\mu_r} & \frac{\alpha_{rv}}{\mu_r} & 0 \end{bmatrix}$$

$$R_{0vr} = \frac{R_{0r} + \sqrt{R_{0r}^2 + 4R_{0v}}}{2},$$

where $R_{0r} = \dfrac{B_r\mu_r q + \beta_{rr}\sigma_r}{B_r\mu_r}$ and $R_{0v} = \dfrac{K\beta_{vr}\alpha_r\sigma_r(\Omega_r-\mu_r)}{B_r\mu_r\mu_v\Omega_r}$.

Theorem S1.11. *The disease-free equilibrium* $DFE_{rv} = (S_{r0}, E_{r0}, I_{r0}, V_0) = (\frac{K(\Omega_r-\mu_r)}{\Omega_r}, 0, 0, 0)$*, of the rodent-virus subsystem (1.2) is globally asymptotically stable when* $R_{0vr} < 1$.

Proof. (of (S1.11)
Similar to the proof of thm 2.2 we use Lyapunov function. Define $L = E_r + m_1 I_r + m_2 V$ then,

$$\dot{L} = \dot{E}_r + m_1\dot{I}_r + m_2\dot{V} \le \left(\beta_{rr}I_r + \beta_{vr}V\tfrac{(\Omega_r-\mu_r)K}{\Omega_r} - B_rE_r\right) + (q\mu_rI_r + \sigma_rE_r - \mu_rI_r) + (\alpha_{rv}I_r - \mu_vV)$$
$$= (\beta_{rr} + m_1q\mu_r + m_2\alpha_{rv} - m_1\mu_r)I_r + (m_1\sigma_r - B_r)E_r + (\tfrac{\beta_{vr}(\Omega_r-\mu_r)K}{\Omega_r} - \mu_vm_2)V.$$ Set $m_1 = \frac{B_r}{\sigma_r}, m_2 = \frac{\beta_{vr}(\Omega_r-\mu_r)K}{\Omega_r\mu_v}$ yields:
$$\dot{L} \le \tfrac{\mu_rB_r}{\sigma_r}\left[\tfrac{\beta_{rr}\sigma_r}{B_r\mu_r} + q + \tfrac{\beta_{vr}(\Omega_r-\mu_r)K\alpha_{rv}\sigma_r}{B_r\mu_r\mu_v\Omega_r} - 1\right]I_r = \tfrac{\mu_rB_r}{\sigma_r}\left[R_{0r} + R_{0v} - 1\right]I_r.$$

Notice that $R_{0vr} \le 1 \implies R_{0r} + R_{0v} \le 1$. Therefore $\dot{L} < 0$ if $R_{0vr} \le 1$

The next theorem establishes the existence, uniqueness of the endemic equilibrium of the rodent-virus sub-system (S1.9).

Theorem S1.12. *The rodent-virus sub-system* (S1.9) *admits at most one endemic equilibrium whenever* $R_{0rv} > 1$.

Proof. Let $E_{rv}^* = (S_r^*, E_r^*, I_r^*, V^*)$ be an endemic equilibrium of the rodent-virus sub-system (S1.9). Specifically, let E_{rv}^* be an equilibrium for the limiting system of the model (S1.9) obtained by replacing N_r in the model system by $\tilde{N}_r = \lim_{t\to+\infty} N_r(t) = \frac{K(\Omega_r-\mu_r)}{\Omega_r}$ and by setting $S_r = \tilde{N}_r - E_r - I_r$. This yields the reduced system:

$$\begin{aligned} \dot{E}_r &= \left(\frac{\beta_{rr}I_r}{\tilde{N}_r} + \beta_{vr}V\right)(\tilde{N}_r - E_r - I_r) - B_rE_r, \\ \dot{I}_r &= q\Omega_r(1 - \frac{\tilde{N}_r}{K})I_r + \sigma_rE_r - \mu_rI_r, \\ \dot{V} &= \alpha_{rv}I_r - \mu_vV. \end{aligned} \tag{S1.10}$$

Setting the right hand-sides of this sub-system to zero, and solving for V and E_r in terms of I_r yields the endemic equilibrium:

$$I_r^* = \frac{\tilde{N}_r\sigma_r\left(1 - \frac{(1-q)}{R_{0rv}}\right)}{\mu_r(1-q) + \sigma_r}, \quad V^* = \frac{\alpha_{rv}I_r^*}{\mu_v}, \quad E_r^* = \frac{\mu_r(1-q)I_r^*}{\sigma_r}, \quad S_r^* = \tilde{N}_r - E_r^* - I_r^*,$$

where $B_r = \sigma_r + \mu_r$ and I_r^*, which exists (i.e., is positive) when $R_{0rv} > 1$.

□

S4 The model with no environmental contamination or transmission

The simplified model with no environmental contamination or transmission is obtained by setting the state variable V to zero in the model system (S1.1) :

$$
\begin{aligned}
\dot{S_h} &= \Lambda_h - \left(\frac{\beta_{ah}I_{ah}+\beta_{sh}I_{sh}+\beta_{ch}I_{ch}}{N_h}+\frac{\beta_{rh}I_r}{N_r}\right)S_h - \mu_h S_h\\
\dot{E_h} &= \left(\frac{\beta_{ah}I_{ah}+\beta_{sh}I_{sh}+\beta_{ch}I_{ch}}{N_h}+\frac{\beta_{rh}I_r}{N_r}\right)S_h - (\mu_h+\sigma_h)E_h,\\
\dot{I_{ah}} &= \tau\,\sigma_h E_h - (\mu_h+\gamma_{ah})\,I_{ah},\\
\dot{I_{sh}} &= (1-\tau)\,\sigma_h E_h - (\mu_h+\gamma_{sh}+\rho_{sh}+\delta_{sh})I_{sh},\\
\dot{I_{ch}} &= \rho_{sh}I_{sh} - (\mu_h+\gamma_{ch}+\delta_{ch})I_{ch},\\
\dot{R_h} &= \gamma_{ch}I_{ch}+\gamma_{sh}I_{sh}+\gamma_{ah}I_{ah}-\mu_h R_h,\\
\dot{S_r} &= \Omega_r(1-\frac{N_r}{K})\,[S_r+E_r+(1-q)I_r] - \frac{\beta_{rr}I_r}{N_r}S_r - \mu_r S_r,\\
\dot{E_r} &= \frac{\beta_{rr}I_r}{N_r}S_r - (\sigma_r+\mu_r)E_r,\\
\dot{I_r} &= q\Omega_r\left(1-\frac{N_r}{K}\right)I_r + \sigma_r E_r - \mu_r I_r,
\end{aligned}
\tag{S1.11}
$$

This is used with the next generation operator approach to compute the basic reproduction number of the model.
Specifically, the matrices of new infections ($\mathscr{F}$) and transitions ($\mathscr{V}$) are given by

$$
\mathscr{F} = \begin{pmatrix}
0 & \beta_{sh} & \beta_{ah} & \beta_{ch} & 0 & \frac{\beta_{rh}\Lambda_h\Omega_r}{K(\Omega_r-\mu_r)\mu_h}\\
0 & 0 & 0 & 0 & 0 & 0\\
0 & 0 & 0 & 0 & 0 & 0\\
0 & 0 & 0 & 0 & 0 & 0\\
0 & 0 & 0 & 0 & 0 & \beta_{rr}\\
0 & 0 & 0 & 0 & 0 & q\mu_r
\end{pmatrix}
\text{ and } \mathscr{V} = \begin{pmatrix}
B_e & 0 & 0 & 0 & 0 & 0\\
(1-\tau)\sigma_h & B_s & 0 & 0 & 0 & 0\\
-\tau\sigma_h & 0 & B_a & 0 & 0 & 0\\
0 & -\rho_{sh} & 0 & B_c & 0 & 0\\
0 & 0 & 0 & 0 & B_r & 0\\
0 & 0 & 0 & 0 & -\sigma_r & \mu_r
\end{pmatrix},
$$

$$
\mathscr{F}\mathscr{V}^{-1} = \begin{pmatrix}
\frac{\sigma_h[\tau\beta_{ah}B_cB_s+(1-\tau)B_a(\beta_{sh}B_c+\beta_{ch}\rho_{sh})]}{B_aB_cB_eB_s} & \frac{\beta_{sh}B_c+\beta_{ch}\rho_{sh}}{B_cB_s} & \frac{\beta_{ah}}{B_a} & \frac{\beta_{ch}}{B_c} & \frac{\beta_{rh}S_h\Omega_r\sigma_r}{K(\Omega_r-\mu_r)B_r\mu_r} & \frac{\beta_{rh}S_h\Omega_r}{K(\Omega_r-\mu_r)\mu_r}\\
0 & 0 & 0 & 0 & 0 & 0\\
0 & 0 & 0 & 0 & 0 & 0\\
0 & 0 & 0 & 0 & 0 & 0\\
0 & 0 & 0 & 0 & \frac{\beta_{rr}\sigma_r}{B_r\mu_r} & \frac{\beta_{rr}}{\mu_r}\\
0 & 0 & 0 & 0 & \frac{q\sigma_r}{B_r} & q
\end{pmatrix}.
$$

The basic reproduction number of model is

$$
\mathscr{R}_0 = \max(R_{0h}, R_0^r), \tag{S1.12}
$$

where $R_{0h} = \dfrac{\sigma_h\,[(1-\tau)\,B_a\,(\beta_{sh}B_c+\beta_{ch}\rho_{sh})+\tau\beta_{ah}B_cB_s]}{B_sB_eB_aB_c}$ and $R_0^r = \dfrac{B_r\mu_r q+\beta_{rr}\sigma_r}{B_r\mu_r}$. It should be noted that that R_{0h} and R_0^r have been written to emphasize the contributions of symptomatic, confirmed, and asymptomatic infectious human transmission, as well as regular rodent and vertical rodent transmission on the reproduction number.

Theorem S1.13. *The DFE of the model with no environment contamination is globally stable whenever its reproduction number, $\mathscr{R}_0$ is less than one.*

Proof. We proved this by using Lyapunov function construction method. To do so, we considered the following Lyapunov function: $L = E_h + n_1 I_{ah} + n_2 I_{sh} + n_3 I_{ch} + n_4 I_r + n_5 E_r$ with n_1, n_2, n_3 and n_4 where n_1, n_2, n_3 and n_4 are to be determined for the function L defined on $\mathscr{R}$ to be decreasing. We have:

$$\dot{L} = \dot{E_h} + n_1 \dot{I_{ah}} + n_2 \dot{I_{sh}} + n_3 \dot{I_{ch}} + n_4 \dot{I_r} + n_5 \dot{E_r} \tag{S1.13}$$

By replacing the expression of $\dot{E_h}, \dot{I_{ah}}, \dot{I_{sh}}, \dot{I_{ch}}, \dot{E_r}$, and $\dot{I_r}$ from no environmental transmission model in (S1.13) and knowing that $\frac{S_h}{N_h} \leq 1$, $\frac{S_r}{N_r} \leq 1$ and $N_r = S_r^0 = \frac{(\Omega_r - \mu_r)K}{\Omega_r}$ (at the DFE). Then,
$\dot{L} = \dot{E_h} + n_1 \dot{I_{ah}} + n_2 \dot{I_{sh}} + n_3 \dot{I_{ch}} + n_5 \dot{E_r} + n_4 \dot{I_r}$
$\leq (\beta_{sh} I_{sh} + \beta_{ch} I_{ch} + \frac{\Lambda_h \beta_{rh} \Omega_r I_r}{\mu_h(\Omega_r - \mu_r)K} - B_e E_h) + n_1(\tau \sigma_h E_h - B_a I_{ah}) + n_2((1-\tau)\sigma_h E_h - B_s I_{sh}) + n_3(\rho_{sh} I_{sh} - B_c I_{ch}) + n_5(\beta_{rr} I_r - B_r E_r) + n_4(q\mu_r I_r + \sigma_r E_r - \mu_r I_r)$
$= (\beta_{ah} - n_1 B_a) I_{ah} + (\beta_{ch} - n_3 B_c) I_{ch} + (\beta_{sh} - n_2 B_s + n_3 \rho_{sh}) I_{sh} + (n_2 \tau \sigma_h + n_3 (1-\tau)\sigma_h) E_h$
$+ [\frac{\Lambda_h \beta_{rh} \Omega_r}{\mu_h(\Omega_r - \mu_r)K} + n_5 \beta_{rr} - n_4 \mu_r (1-q)] I_r + (n_4 \sigma_r - n_5 B_r) E_r$ Now, since we want to choose the n_i's so that $\dot{L} < 0$, we eliminated the first three terms in the last equality by setting $n_1 = \frac{\beta ah}{B_a}, n_3 = \frac{\beta ch}{B_c}$ and $n_2 = \frac{\beta sh B_c + \beta ch \rho_{ch}}{B_c B_s}$. Setting the 5th term in the same equality to zero and the coefficient in front of I_r in the 5th term equal to $R_0^r - 1$ yields a system of two equations with unknown n_4 and n_5. The solution of this system of two equations are: $n_4 = \frac{(a + n_5)\beta_{rr}}{(1-q)\mu_r}$ and $n_5 = \frac{R_0^r - 1 - ab}{b - B_r}$ where $a = \frac{\Lambda_h \beta_{rh} \Omega_r \sigma_r}{B_r \mu_r \mu_h (\Omega_r - \mu_r)K}$, $b = \frac{\beta_{rr} \sigma_r}{(1-q)\mu_r}$. Hence, $n_4, n_5 > 0$ whenever $R_0^r < 1$ and $B_r > b$ By replacing the n_i's, only the 4th and 6th terms of the later equality survived and we have :

$$\dot{L} \leq B_e \left[\frac{\sigma_h [(1-\tau) B_a (\beta_{sh} B_c + \beta_{ch} \rho_{sh}) + \tau \beta_{ah} B_c B_s]}{B_s B_e B_a B_c} - 1 \right] E_h + [R_{0r} - 1] E_r = Be(R_{0h} - 1) E_h + (R_0^r - 1) I_r.$$

Since $\mathscr{R}_0$=max(R_{0h}, R_0^r) it follows that whenever $\mathscr{R}_0 < 1$ we have $\dot{L} < 0$.

□

S5 The model with rodent competitor

Let N_c be the total rodent competitor population. Then the extended system that accounts for the competitor dynamics is obtained by replacing the rodent subsystem of the full model (S1.1) with the subsystem:

$$\begin{aligned}
\dot{S_r} &= \Omega_r \left(1 - \frac{N_r + \zeta_c N_c}{K_r}\right) [S_r + E_r + (1-q) I_r] - \left(\frac{\beta_{rr} I_r}{N_r} + \beta_{vr} V\right) S_r - \mu_r S_r, \\
\dot{E_r} &= \left(\frac{\beta_{rr} I_r}{N_r} + \beta_{vr} V\right) S_r - (\sigma_r + \mu_r) E_r, \\
\dot{I_r} &= q\Omega_r \left(1 - \frac{N_r + \zeta_c N_c}{K_r}\right) I_r + \sigma_r E_r - \mu_r I_r, \\
\dot{N_c} &= \Omega_c \left(1 - \frac{N_c + \zeta_r N_r}{K_c}\right) N_c - \mu_m N_c,
\end{aligned} \tag{S1.14}$$

where K_c is the carrying capacity of the rodent competitor, ζ_r (ζ_c) competition coefficient depicting the impact of rodents (competitors) on competitors (rodents), Ω_c is the intrinsic growth rate of the rodent competitor, and μ_c is the natural mortality rate of the rodent competitor. Since implementing this strategy can offer a sustainable and long-term reduction in Lassa fever incidence, the extended model (i.e., the full model (S1.1) with the rodent sub-system replaced by the subsystem (S1.14)) is simulated to assess the impact of introducing a superior competitor on the dynamics of LF.

S6 Numerical results

Table S3. Initial condition (at 12/29/2019) values of the state variables and the values at the disease endemic equilibrium (DEE).

State variables	S_h	E_h	I_{ah}	I_{sh}	I_{ch}	R_h
Initial condition	208,248,484	500	800	200	16	50,000
$^*DEE$	6,463,626	137,729	81,576	32,331	1	193,213,725

State variables	S_r	E_r	I_r	V	
Initial condition	2,489,126	1,000	100,000	1,650	
$^*DEE$	116,275	82,660	2,290,192	38	

$^*DEE$:=Disease Endemic Equilibrium

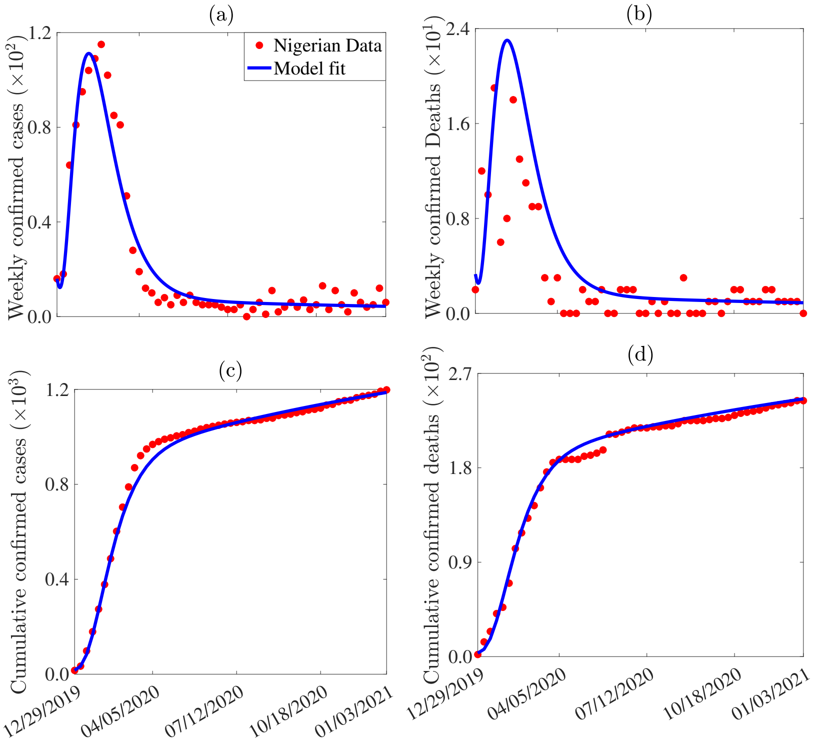

Figure 1. Model fitting to data and validation. (a) Model fitting to observed weekly Lassa fever virus data (red dots) for the epidemic episode from December 15, 2019 to March 07, 2020. (b) Simulations of Model S1.1 using the fixed and estimated parameters from Tables 2 & 3 in the main paper demonstrate a strong alignment between observed cumulative case data (red dots) and model output (blue curve). Additionally, the simulations in (c)-(d) depict a close correspondence between observed weekly and cumulative LF mortality data (red dots) and the corresponding model-derived deaths (blue curves), further validating the model's efficacy.

Table S4. LF data per Epi-weeks

Year	2019	2020	2020	2020	2020	2020	2020	2020	2020	2020	2020
Epi-Week	52	1	2	3	4	5	6	7	8	9	10
Total Confirmed	16	18	64	81	95	104	109	115	102	85	81
Death	2	12	10	19	6	8	18	13	11	9	9
Year	2020	2020	2020	2020	2020	2020	2020	2020	2020	2020	2020
Epi-Week	11	12	13	14	15	16	17	18	19	20	21
Total Confirmed	51	28	19	12	10	6	8	5	9	6	9
Death	3	1	3	0	0	0	2	1	1	2	0
Year	2020	2020	2020	2020	2020	2020	2020	2020	2020	2020	2020
Epi-Week	22	23	24	25	26	27	28	29	30	31	32
Total Confirmed	6	5	5	5	4	3	3	5	0	3	6
Death	0	2	2	2	0	0	1	0	1	0	0
Year	2020	2020	2020	2020	2020	2020	2020	2020	2020	2020	2020
Epi-Week	33	34	35	36	37	38	39	40	41	42	43
Total Confirmed	1	11	2	4	6	4	7	3	5	13	3
Death	3	0	0	0	1	1	0	1	2	2	1
Year	2020	2020	2020	2020	2020	2020	2020	2020	2020		
Epi-Week	44	45	46	47	48	49	50	51	52		
Total Confirmed	11	5	2	10	6	4	5	12	6		
Death	1	1	2	2	1	1	1	1	0		

Source: LF situation reports from Nigeria Centre for Disease Control and Prevention (NCDC).

□

References

1. H. L. Smith, H. R. Thieme, Dynamical systems and population persistence, Vol. 118, American Mathematical Soc., 2011.
2. Dieckmann, U. (2002). Adaptive dynamics of pathogen-host interactions. IR-02-007
3. O. Diekmann, J. Heesterbeek, M. G. Roberts, The construction of next-generation matrices for compartmental epidemic models, Journal of the royal society interface 7 (47) (2010) 873–885.
4. Van den Driessche, P., & Watmough, J. (2002). Reproduction numbers and sub-threshold endemic equilibria for compartmental models of disease transmission. Mathematical biosciences, 180(1-2), 29-48.
